# An Interpretable Classification Model Using Gluten-Specific TCR Sequences Shows Diagnostic Potential in Coeliac Disease

**DOI:** 10.3390/biom13121707

**Published:** 2023-11-25

**Authors:** Anna Fowler, Michael FitzPatrick, Aberami Shanmugarasa, Amro Sayed Fadel Ibrahim, Hannah Kockelbergh, Han-Chieh Yang, Amelia Williams-Walker, Kim Ngan Luu Hoang, Shelley Evans, Nicholas Provine, Paul Klenerman, Elizabeth J. Soilleux

**Affiliations:** 1 Department of Health Data Science, Institute of Population Health, University of Liverpool, Liverpool L69 3GF, UK; 2 Translational Gastroenterology Unit, Nuffield Department of Medicine, University of Oxford, Oxford OX3 9DU, UK; michael.fitzpatrick@ndm.ox.ac.uk (M.F.); paul.klenerman@medawar.ox.ac.uk (P.K.); 3 School of Clinical Medicine, University of Cambridge, Cambridge CB2 0SP, UK; as2750@cam.ac.uk; 4 Department of Pathology, University of Cambridge, Cambridge CB2 1QP, UK; asfi2@cam.ac.uk (A.S.F.I.); hcy23@cam.ac.uk (H.-C.Y.); abw208@exeter.ac.uk (A.W.-W.); knl25@cam.ac.uk (K.N.L.H.); sce30@cam.ac.uk (S.E.); ejs17@cam.ac.uk (E.J.S.); 5 Peter Medawar Building for Pathogen Research, University of Oxford, Oxford OX1 3SY, UK

**Keywords:** coeliac disease, T-cell repertoire, machine learning, next generation sequencing, gluten-free diet

## Abstract

Coeliac disease (CeD) is a T-cell mediated enteropathy triggered by dietary gluten which remains substantially under-diagnosed around the world. The diagnostic gold-standard requires histological assessment of intestinal biopsies taken at endoscopy while consuming a gluten-containing diet. However, there is a lack of concordance between pathologists in histological assessment, and both endoscopy and gluten challenge are burdensome and unpleasant for patients. Identification of gluten-specific T-cell receptors (TCRs) in the TCR repertoire could provide a less subjective diagnostic test, and potentially remove the need to consume gluten. We review published gluten-specific TCR sequences, and develop an interpretable machine learning model to investigate their diagnostic potential. To investigate this, we sequenced the TCR repertoires of mucosal CD4^+^ T cells from 20 patients with and without CeD. These data were used as a training dataset to develop the model, then an independently published dataset of 20 patients was used as the testing dataset. We determined that this model has a training accuracy of 100% and testing accuracy of 80% for the diagnosis of CeD, including in patients on a gluten-free diet (GFD). We identified 20 CD4^+^ TCR sequences with the highest diagnostic potential for CeD. The sequences identified here have the potential to provide an objective diagnostic test for CeD, which does not require the consumption of gluten.

## 1. Introduction

Coeliac disease (CeD) is a chronic inflammatory disorder of the small intestine that develops when a genetically susceptible individual is exposed to gluten proteins found in certain cereal grains including wheat, rye and barley. Gluten is the collective term for a number of proteins, including gliadin and glutenin in wheat, hordein in barley, and secalin in rye [1,2]. In the gastrointestinal tract, gliadin is hydrolysed into gliadin peptide fragments, which are deamidated by the autoantigen tissue transglutaminase, tTG, producing highly immunogenic deamidated gliadin peptides. These deamidated gliadin peptides, just like antigens from invading pathogens, are endocytosed and presented on the major histocompatibility complex (MHC; also known as HLA) on the surface of the intestinal epithelial cells. The subsequent interaction between MHC-bound gliadin peptide antigens and CD4^+^ T cells is thought to drive the inflammation, which damages the small intestine, causing atrophy (shortening and broadening) of the villi, leading to an array of symptoms, including malabsorption and diarrhea [3].

Each T cell has a T cell receptor (TCR) on its surface which determines antigen recognition; a set of TCRs is known as a T cell receptor repertoire and is necessarily diverse to allow for the recognition of a wide range of antigens. This diversity is generated through somatic TCR rearrangement, as illustrated in Supplementary Figure S1, with the most variable region known as the complementarity determining region 3 (CDR3). T cell receptors have two chains, according to which T cells can be categorised. The TCRs of αβ T cells are encoded by TRA and TRB genes, respectively, while the TCRs of γδ T cells are encoded by TRG and TRD genes [4]. The diversity of CDR3 sequences in a TCR repertoire can be assessed by using bulk or single-cell sequencing, and the starting material can be either DNA or RNA. The utility and advantages of these methods are reviewed in [5].

αβ T cells can also be categorised further as helper (CD4^+^) or cytotoxic (CD8+) T cells based on the expression of the co-receptor, either CD4 or CD8, in the TCR complex [6]. αβ T cells can recognise intracellularly processed antigens bound to MHC complexes found on the cell membrane, forming a complex with class II MHC molecules (MHCII) (in the case of CD4^+^ T cells) or class I MHC molecules (MHC I) (in the case of CD8+ T cells) [7]. In contrast to αβ T cells, γδ T cells do not require interaction with an MHC complex for antigen binding [8]. However, the precise mechanism for antigen recognition in γδ T cells is not yet fully understood.

**Figure 1:**
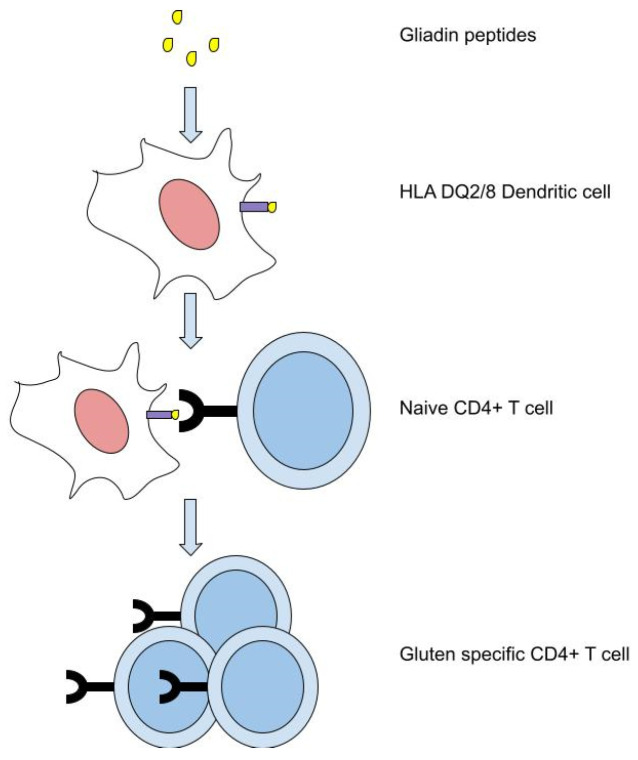
DQ2/8 HLA molecules present gliadin peptides which subsequently activate naive CD4^+^ T cells, which proliferate as gluten specific CD4^+^ T cells.

The genetic susceptibility of developing CeD is most strongly associated with the HLA haplotypes, with the majority (around 80 to 95%) of CeD patients possessing HLA-DQ2 (DQA1*05/DQB1*02) and a minority possessing DQ8 (DQA1*03/DQB1*0302) [9]. These HLA molecules are capable of presenting an array of gliadin peptides which subsequently interact with gluten-reactive CD4^+^ T cells; see Figure 1. Among these T cell epitopes of the gluten protein (consisting of α-, γ-, and ω-gliadin and glutenin components), some appear to be more important than others in CeD development. For instance, T cells specific for αI and αII epitopes of α-gliadin are found in almost all CeD patients, while the epitopes of γ-gliadin are less often recognised [10]. Therefore, based on these CeD-specific cognate interactions, a synthetic antigen presenting an MHC-tetramer complex loaded with gluten epitopes can be designed to selectively label the gluten reactive CD4^+^ T cells from a T cell repertoire, using a recombinant MHC tetramer assay [11]. However, this approach requires a large blood sample and is labour intensive [12]. DNA sequencing can then be performed on these gluten reactive CD4^+^ T cells to determine their αβ-TCR sequences, and these gluten-specific TCR sequences may provide diagnostic potential for CeD. Despite the diversity of the TCR repertoire, the observed degree of sharing between individuals is higher than expected at random [13]. One explanation for this is their immune functionality, and that individuals responding to the same immune stimulus are more likely to share TCRs [14]. In the case of CeD patients, they may share TCRs capable of responding to gluten. However, it is also possible that different TCRs bind the same gluten epitope due to shared genetic motifs or shared amino acid properties [15]; therefore, multiple gluten-specific TCR sequences need to be considered.

While the biological mechanisms of CeD have been widely studied, diagnosis remains a challenge. In many countries, blood testing for both anti-TTG and anti-EMA antibodies are undertaken, followed by endoscopic duodenal biopsy, with patients being required to eat a significant amount of gluten for 2–6 weeks prior to biopsy (NICE, ESPGAN, [16]). Many patients struggle to tolerate gluten challenge due to the severity of their gluten-induced symptoms, meaning that antibody levels may be close to or below the upper limit of normal and biopsy changes may be subtle and non-diagnostic, resulting in either inconclusive tests and patients requiring further investigation, or incorrect diagnosis as non-coeliac [17]. This results in a low sensitivity, with many cases of CeD remaining undiagnosed. While histopathological examination of a duodenal biopsy remains the “gold standard test”, studies indicate that concordance between pathologists for the diagnosis of CeD is around 80%, mainly due to the subjective interpretation of subtle biopsy change [18,19,20]. There is a clear need for a novel method of diagnosis which does not require the consumption of gluten, and therefore is more sensitive, and more objective. A test which could also be performed on a blood sample, rather than requiring a duodenal biopsy, would provide further improvements on the current “gold standard”.

Our recent work indicates that patterns of T-cell receptor repertoire γ and δ sequences can be used to separate duodenal biopsy samples from patients with CeD from controls, even if the patient is on a gluten-free diet (GFD), suggesting that TCR repertoire sequencing could form the basis of a more reliable diagnostic test, regardless of dietary gluten status [21]. However, this method lacks interpretability and transforms the data, making it difficult to determine which sequences are driving the classification. In view of this, we review here currently published gluten-specific TCR sequences and determine their frequency in a training dataset of TCR repertories to assess their diagnostic potential. These sequences are then validated in an independent testing dataset, and a short list of TCR sequences which are likely coeliac-specific is generated.

## 2. Materials and Methods

### 2.1. Identification of Published Coeliac-Specific TCR Sequences

We performed a literature search (February 2023) to identify all published TCR sequences capable of binding HLA-DQ2.2, HLA-DQ2.5, and HLA-DQ8 gluten peptides in CeD patients. This was conducted using a MedLine and Scopus search of all papers containing both TCR (or T cell receptor or synonyms) and coeliac disease (or celiac disease). These papers were then manually filtered for those which published sequences identified using tetramers. All loci were considered, as well as either paired or un-paired sequences. Only full CDR3 sequences published within the papers or Supplementary Materials were included.

### 2.2. Cohort 1: Training Dataset

#### 2.2.1. Study Subjects

Study subjects with CeD undergoing upper GI endoscopy were identified via the Oxford University Hospitals NHS Foundation Trust (OUHFT) coeliac disease service (Oxford, UK). Control subjects were identified through the OUHFT endoscopy service; these were patients undergoing endoscopy with gastrointestinal symptoms who were not diagnosed with CeD. Intestinal biopsies were taken at endoscopy under the study Oxford Gastrointestinal Illnesses Biobank (REC Ref: 16/YH/0247). Patient characteristics are described in Supplementary Table S1.

#### 2.2.2. Processing Intestinal Biopsies

Duodenal biopsies were collected at endoscopy and transported in R10 (RPMI-1640 [Lonza, Basel, Switzerland] with 10% FCS [Sigma-Aldrich, St. Louis, MO, USA] plus 1% penicillin/streptomycin [Sigma-Aldrich]). Lymphocytes were isolated from intestinal mucosal biopsies as previously described in [22]. Briefly, duodenal samples were incubated in R10 with 1 mg/mL Collagenase D (Roche, Basel, Switzerland) and 100 mg/mL DNase (Thermo Fisher Scientific, Waltham, MA, USA) for 1 h at 37 °C while agitated in a shaking incubator. Biopsies were then dissociated using a GentleMACS Dissociator (Miltenyi Biotec, Bergisch Gladbach, Germany), and strained through a 70 μm filter. Cells were washed twice with R10 media.

#### 2.2.3. Cell Sorting and Sequencing

For surface marker staining, intestinal lymphocytes were stained in FACS buffer (PBS + 1 mM EDTA + 0.05% BSA) for 20–30 min at 4 °C, with the following antibodies, then stored at 4 °C protected from light before cell sorting: anti-CD45 (HI30, Biolegend, San Diego, CA, USA), anti-CD3 (UCHT1, Biolegend), anti-αβTCR (IP26, BD Biosciences, Franklin Lakes, NJ, USA), anti-γδTCR (11F2, Miltenyi Biotec), anti-CD4 (OKT4, Biolegend), anti-CD8 (SK1, Biolegend). For fluorescence-activated cell sorting (FACS), samples were surface stained, with DAPI (Thermo Fisher Scientific) used as a viability dye. FACS was performed on an AriaIII (BD Biosciences; 70 μm nozzle). Intestinal CD4^+^ T cells (CD3+, αβ+, γδ−, CD4^+^, CD8−, live lymphocytes) were sorted; an example of the T cell sorting gate is provided in Supplementary Figure S6. Sort purity was assessed for each sort, and was >98% in all cases. Sorted cells were collected in PBS with 2% BSA before Trizol nucleic acid extraction.

A Trizol RNA extraction was used for low numbers of lymphocytes, as previously described [23,24], except that 1.5 mL Eppendorf tubes replaced phase-lock gel tubes. Briefly, after sorting, cells were centrifuged (500 g, 5 min), resuspended in 1 mL Trizol, then frozen at −80 °C. For RNA extraction, samples were brought to room temperature, mixed with 200 μL chloroform, and centrifuged at 12,500 rpm for 5 min. Then, 500 μL of the aqueous phase was taken and RNA extracted using the Agencourt RNAdvance Tissue Isolation kit (Beckman Coulter, Indianapolis, IN, USA). RNA concentration and purity for samples with larger cell numbers were assessed using a 2100 Bioanalyzer instrument (Agilent Technologies, Santa Clara, CA, USA), with RIN values between 7.6 and 9.4.

Bulk TCR repertoire sequencing was performed using the amplicon-rescued multiplex (ARM)-PCR method (iRepertoire, Inc., Huntsville, AL, USA). This method performs an initial first-round RT-PCR with TCR V and C gene-specific primers for the relevant TCR chain, followed by further amplification steps with universal primers for the exponential phase of amplification. This method is designed to provide quantitative, deep sequencing of the TCR repertoire, with minimal bias.

Library generation was performed in-house following manufacturer’s instructions, except for performing the library generation in 96-well plates. The quality, size distribution, concentration, and presence of contaminating primer dimers was assessed using gel electrophoresis, spectral photometry (Nanodrop, Thermo Fisher Scientific, Waltham, MA, USA), and a 2100 Bioanalyzer (Agilent Technologies, Santa Clara, CA, USA). Libraries were quantified using the KAPA Library Quantification Kit (Roche) before equimolar pooling. A PhiX library 10% spike-in was added, before 300 bp paired-end sequencing on an Illumina MiSeq instrument at the Oxford Genomics Centre (WTCHG, University of Oxford).

### 2.3. Cohort 2: Testing Dataset

An independent dataset was used as a testing dataset; this has been previously described in [25] and is publicly available (SRA project ID: PRJNA678347). Briefly, duodenal biopsies were collected from 9 untreated CeD patients, and 6 non-CeD controls. The epithelial layer was removed, prior to collagenase digestion and homogenisation of the lamina propria layer. Filtration through a cell strainer permitted recovery of a lamina propria cell suspension, from which cDNA was synthesised. Following cDNA synthesis, two semi-nested TCRα- and TCRβ-specific PCR reactions were carried out as in [26]. Note that in the original publication [25], CeD patients with HLA-DQ8 were treated as controls as the training data did not contain any patients with HLA-DQ8; however, here they are treated as CeD patients as the training data here does contain patients with HLA-DQ8.

### 2.4. Data Processing

Both datasets were processed using the MiXCR v.3.0.7 [27] ‘analyze amplicon’ command, which identifies the CDR3 region of reads and performs clonotype assembly in order to correct for sequencing and PCR errors.

Abundance, richness, Shannon diversity, Simpson diversity, and V and J segment usage were determined for all datasets using Python 3.10.8 [28] with Pandas 1.5.1 [29] and Numpy 1.23.4 [30]. A clone was defined as a collection of receptors with the same V segment, J segment, and CDR3 sequence at the amino acid level. These measures were plotted using Matplotlib 3.6.0 [31]. V and J segment usage heatmaps additionally used Seaborn 0.12.1 [32].

Further, Student’s *t*-test was performed to test the hypothesis that CeD and non-CeD samples have different means for the 3 diversity measures and for each V and J segment. To account for multiple tests, the Bonferroni correction [33] was applied. A significance threshold was set at 0.05.

### 2.5. Evaluation Measures

Sensitivity, defined as the fraction of CeD samples correctly classified by the algorithm, and specificity, defined as the fraction of non-CeD controls correctly classified by the algorithm, were used to evaluate performance. These two measures were also combined to give an overall accuracy, defined as the fraction of all samples correctly classified by the algorithm. In training the algorithm, we sought to identify parameters which optimised accuracy on the training dataset. Then the algorithm was tested by calculating the accuracy, using the identified parameters, on an independent testing dataset.

### 2.6. Training, Cross-Validation, and Testing Classification Model with Feature Selection

We use the set of all published gluten-specific sequences as features, and aim to select those which are predictive of CeD and therefore have diagnostic potential. In order to do this, we developed a classification model which seeks to label patients as either CeD or control based on the frequency of a subset of the published gluten-specific sequences. This model is a simple decision rule which classifies samples as CeD based on whether or not they contain at least one of a set of coeliac-predictive sequences. This decision rule allows us not only to classify the samples, but also to identify the coeliac-predictive sequences upon which classification is based. The parameters of this model are the subset of the published gluten-specific sequences on which classification is based (which we refer to as coeliac-predictive sequences) and a threshold value to determine the presence in a sample. These parameters are optimised and validated on cohort 1, the training dataset. Leave-one-out cross-validation is then used on cohort 1 to determine the stability of these parameters and their sensitivity to changes in the training set. Finally, the trained model is applied to an independent testing dataset, cohort 2, to test whether the results apply beyond a single study.

Firstly, we calculated the ranked clonotype frequency of all sequences in a repertoire; when the clonotypes are ordered from most to least frequent, the ranked clonotype frequency for the *i*^th^ clonotype is defined as *r_i_* = *i*/*N*, where *N* is the total number of clonotypes. This measure accounts for different sequencing depths between samples, as well as differing clone sizes. The most frequent clonotypes, the ones which are highly ranked, will have a low ranked-frequency close to 0, and the smallest clonotypes will have a large ranked-frequency, close to 1. This is performed independently for each sample.

Next, we trained a classification model on the ranked clonotype frequencies of cohort 1, the training dataset, using the known status of patients as CeD or controls. Clonotypes were considered coeliac-predictive if their ranked-frequency was below some threshold in at least one CeD patient and in none of the controls. The threshold parameter was optimised to achieve the highest accuracy. Bootstrap replicates were used to provide 95% confidence intervals for the evaluation measures. Leave-one-out cross-validation was then applied to cohort 1 to test the robustness of the model and the sensitivity of parameter estimates to changes in the training data.

This model, containing the threshold parameter and a set of coeliac-specific sequences, was then applied to cohort 2, the testing dataset, blinded to the test set diagnostic labels. Once predictions had been made on cohort 2, the known CeD status was used to assess model performance, using measures of classification accuracy, sensitivity, and specificity. The number of CeD samples in which the coeliac-specific sequences had ranked-frequency below the ranked-frequency threshold (i.e., were ranked highly) was used in order to identify those sequences with the most diagnostic potential.

## 3. Results

### 3.1. CeD Patients Cannot Be Identified through Repertoire Characteristics Alone

The training dataset includes RNA-derived TCR repertoires from sorted mucosal CD4^+^ T cells from duodenal mucosal biopsies from 12 CeD patients, five of whom were on a gluten-free diet, and from eight controls without CeD. Patient characteristics are provided in Supplementary Table S1. The testing dataset consists of pan-CD3+ TCR repertoire samples from cDNA (i.e., RNA) from nine untreated CeD disease patients and six non-CeD individuals. Further information is available in the original publication [25]. A summary of the total number of reads and the number of clones identified in each dataset is given in Table 1.

**Figure 2:**
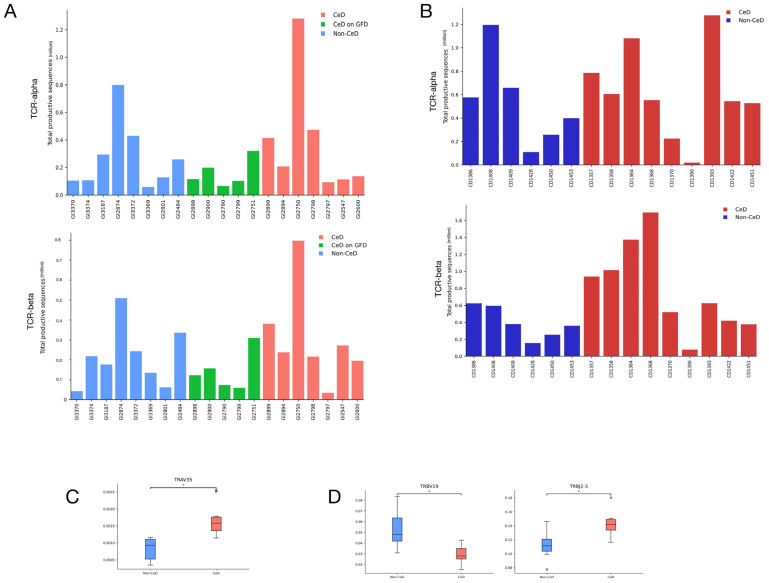
Training and testing dataset summaries. (**A**) Cohort 1, training dataset, productive TCR-α and TCR-β sequence counts for each individual. Red indicates patients with CeD on gluten-containing diets, green indicates patients with CeD on gluten-free diets, and blue indicates controls. (**B**) Cohort 2, testing dataset, productive TCR-α and TCR-β sequence counts for each individual. Red indicates patients with CeD and blue indicates controls. (**C**) Boxplot showing significantly higher TRAV35 segment usage in patients with CeD compared to controls (adjusted *p* = 0.0149, unadjusted *p* = 0.000338), difference below significance level of 0.05 indicated by *, in cohort 1, training dataset. (**D**) Boxplots showing significantly lower TRBV19 (adjusted *p* = 0.0340, unadjusted *p* = 0.000724) and higher TRBJ2-3 (adjusted *p* = 0.0422, unadjusted *p* = 0.00325) segment usage in patients with CeD compared to controls, difference below significance level of 0.05 indicated by *, in cohort 1, training dataset. In (**C**,**D**), patients with CeD on gluten-containing diets and patients with CeD on gluten-free diets were both included in the CeD group as no significant differences were observed between the two subgroups.

In the TCR-α training dataset, significantly higher TRAV35 segment usage was observed in CeD patient samples using Student’s *t*-test, shown in Figure 2C and Figure S2d. In the TCR-β training dataset, we observe a significant difference, lower and higher, respectively, in the TRBV19 and TRBJ2-3 segments in CeD samples compared to non-CeD samples, shown in Figure 2D and Figure S3d,e, using the same methodology. No other V or J segment usage differed significantly between the groups for the training set, nor did any repertoire diversity measures, shown in Figures S2 and S3. None of the V and J segment usage or diversity measures were significantly different between the CeD and non-CeD groups in either testing datasets, shown in Figures S4 and S5. Despite the identification of segments used more or less frequently in CeD patient TCR sequences within the training dataset, we did not observe these findings within the testing dataset. This lack of generalisability might be attributable to differences between library preparation methodology, where sequences with particular V and J gene segments can be preferentially amplified.

In light of the inability of these standard approaches to TCR analysis to distinguish between CeD and non-CeD patient duodenal biopsy samples, we developed a machine-learning model using TCR sequences published as gluten-specific, and trained it using the duodenal CD4^+^ TCR repertoires of the cohort 1 training set. We then applied this trained machine-learning model to the pan-CD3+ TCR repertoires of the cohort 2 testing set for validation. 

### 3.2. Published Gluten-Specific TCRs Are Predominantly TCR-α and TCR-β 

Sixteen studies were identified which contained gluten-specific TCR CDR3 sequences from CeD patients (Table 2 and Table S2). All studies focused on CD4^+^ T cells, but used a range of different gluten epitopes to determine TCR specificity; different epitopes are likely to be bound by TCRs with different CDR3 sequences [34]. In addition to using known gluten epitopes, Christophersen et al. [35] identify gluten-specific cells based on distinct phenotypic properties. This has the advantage of not requiring any prior knowledge of the gluten epitopes. The number of sequences published in each paper varies greatly; those reporting paired sequences publish a smaller number of sequences (with a maximum of 29), whilst those reporting single chains generally publish far more (with a maximum of 437). All papers report sequences from either TCR-α, TCR-β, or both, with the exception of [36], which reports TCR-β and TCR-δ. Therefore, we chose to consider only TCR-α and TCR-β sequences in our analysis. In total, 357 TCR-α and 799 TCR-β sequences were identified from the published literature (Supplementary Table S2), which form the features to potentially be included in the model.

### 3.3. TCR-α Alone Provides 100% Accuracy on Training Dataset

**Figure 3:**
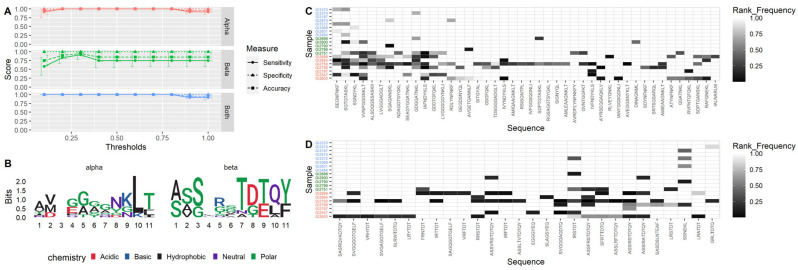
Cohort 1, training dataset. (**A**) Sensitivity, specificity, and accuracy obtained at varying thresholds of ranked-frequency for TCR-α, TCR-β, and both TCR-α and TCR-β in cohort 1, training dataset. (**B**) Logo plots of the sequences TCR-α (left) and TCR-β (right) sequences of length 11 that are included in the model. Logo plots for sequences of all length are included in the Supplementary Figures S7 and S8. (**C**) Heatmap showing ranked-frequency for the sequences included in the TCR-α model with a threshold of 0.6 ranked-frequency, and (**D**) heatmap showing ranked-frequency for the sequences included in the TCR-β model with a threshold of 0.2 ranked-frequency. Red labels indicate patients with CeD on gluten-containing diets, green labels indicate patients with CeD on gluten-free diets, and blue labels indicate controls. Light colours represent absence of the sequence in that individual, whilst dark colours represent the presence of the sequence at high ranked-frequency.

By applying our machine-learning model to TCR sequences published as gluten-specific, we optimized the sensitivity for the identification of CeD by varying the model’s ranked-frequency thresholds (Figure 3A). A threshold of 1 is equivalent to a simple presence/absence test. Using TCR-α alone, 100% accuracy could be achieved across thresholds ranging from 0.1 to 0.7, whereas the highest accuracy achieved using TCR-β was 95% with a threshold of 0.3. Combining both TCR-α and TCR-β sequences also provided 100% accuracy across thresholds ranging from 0.1 to 0.7. The optimal ranked-frequency threshold value was chosen to be 0.6 for TCR-α and combined TCR-α and TCR-β, since this is a central value and therefore robust to variation, and 0.3 for TCR-β, since this had the highest accuracy. The confidence intervals for these optimal parameters were also the tightest, indicating that the results are robust.

Far more TCR-α sequences were included in the model, and therefore considered as more coeliac-predictive than TCR-β, with 44 TCR-α sequences included in comparison to 28 TCR-β. However, the TCR-β sequences included in the model show more evidence of conserved motifs, demonstrated in the logo plots in Figure 3B and Supplementary Figures S7 and S8. The ranked-frequencies of all sequences included in the model are shown in Figure 3C (TCR-α) and Figure 3D (TCR-β). Only six of the coeliac-specific TCR-α sequences, and three of the coeliac-specific TCR-β sequences, were present in any of the normal samples, and these were present at ranked-frequencies below the threshold of 0.6 and 0.3, respectively. The number of coeliac-specific sequences found in a single CeD sample varies greatly, ranging from 2 to 29 for TCR-α and 0 to 17 for TCR-β.

### 3.4. CeD Predicted with 100% Accuracy for Patients on a Gluten-Free Diet

Patients on a gluten-free diet were identified with 100% accuracy using TCR-α, and 80% accuracy using TCR-β, despite them not having consumed any gluten for at least 6 months. However, Figure 3C,D show that those patients on gluten-free diets typically contain fewer unique coeliac-specific sequences than those on gluten-containing diets. The median number of unique TCR-α sequences contained in patients on a gluten-free diet is 2 (IQR 1, 4) in comparison to patients on a gluten-containing diet with a median of 14 (IQR 9, 15.5). The median number of unique TCR-β sequences contained in patients on a gluten-free diet is 1 (IQR 1, 2), in comparison to patients on a gluten-containing diet with a median of 9 (IQR 3.5, 12.5).

### 3.5. Cross-Validation Shows Model Robustness

Leave-one-out cross-validation was applied to cohort 1 to determine the sensitivity of the parameter estimates to changes in the training dataset, and the accuracy results are given in Table 3. TCR-α and combined TCR-α and TCR-β provide the best accuracy results, and the optimal threshold values are consistent with those identified using the whole training dataset. The set of coeliac-predictive sequences is also robust to changes in the training dataset. For TCR-α, 69% of the coeliac-predictive sequences were selected in at least 18 of the cross-validation folds. While for TCR-β, 94% of the coeliac-predictive sequences were selected in at least 18 of the cross-validation folds. The number of folds each of the sequences of interest is present in is also included in Table 4 and Table 5.

### 3.6. Using TCR-α Alone Provides 80% Testing Accuracy

**Figure 4:**
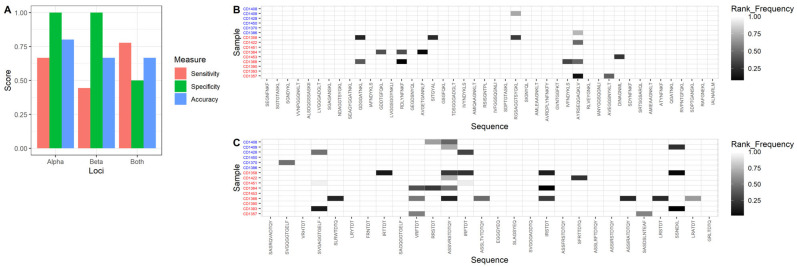
Cohort 2: testing dataset. (**A**) Sensitivity, specificity, and accuracy for TCR-α, TCR-β, and both TCR-α and TCR-β combined for cohort 2, testing dataset. Optimal threshold and coeliac-predictive sequences determined using the training dataset were used. (**B**) Heatmap showing ranked-frequency for the sequences included in the TCR-α model, and (**C**) heatmap showing ranked-frequency for the sequences included in the TCR-β model. Red labels indicate patients with CeD, blue labels indicate controls. Light colours represent absence of the sequence in that individual, whilst dark colours represent the presence of the sequence at high ranked-frequency.

The performance of the coeliac-predictive sequences selected by our model from the set of all previously published sequences and the ranked-frequency threshold value were assessed using cohort 2, the testing dataset. TCR-α was found to have the highest predictive accuracy, of 80%, with 66% sensitivity and 100% specificity (Figure 4A). Combining both TCR-α and TCR-β increased the sensitivity to 78%; however, it led to a large reduction in specificity (50%), and therefore to a lower overall accuracy of 66%. Although TCR-β had a very high specificity (100%), the low sensitivity (44%) resulted in a lower overall accuracy of 50%.

Patients in the testing dataset with HLA-DQ8 are not distinguished from those with HLA-DQ2 since some of the gluten-specific sequences were identified using DQ8-gluten epitopes, and the training dataset contains a sample with HLA-DQ8. Of the two CeD patients in the testing dataset with HLA-DQ8, one was correctly identified and one was not.

### 3.7. Coeliac-Predictive TCR Sequences with Highest Diagnostic Potential Identified

In the testing dataset, there were 10 coeliac-predictive TCR-α sequences and 10 coeliac-predictive TCR-β sequences present in at least one CeD sample and not present in any controls. These are listed in Table 4 and Table 5, respectively, and ranked according to the number of CeD samples from the testing dataset in which they were identified, followed by the number of CeD samples from the training dataset in which they were identified. All of these sequences were identified using DQ2.5 T cell gluten epitopes, except one (AVGETGANNLF) which was identified using a DQ8 T cell gluten epitope. By cataloguing all published gluten-specific sequences, and developing an interpretable machine learning method, we have been able to identify this small number of sequences which we consider to have the highest potential for the diagnosis of CeD.

## 4. Discussion

We developed an interpretable machine-learning model using TCR sequences published as gluten-specific and applied this to intestinal CD4^+^ T cell TCR repertoires and bulk biopsy TCR repertoires. Our model shows diagnostic potential, distinguishing between CeD and controls (with suspected coeliac disease) with 100% training accuracy and 80% testing accuracy. Moreover, the approach shows diagnostic potential for patients on a gluten-free diet, who might be classified as non-coeliac on the basis of serology and biopsy histopathology.

The training dataset contains five patients on a gluten-free diet. Although their samples contain fewer of the gluten-specific sequences, sufficient numbers of unique CeD-associated sequences were present for all patients to be correctly classified. The potential to remove the requirement of gluten challenge, which many patients find unacceptable due to the symptoms it induces, would have considerable benefits and likely increase patient uptake of testing.

The classification model was chosen to not only identify patients with CeD, but also to identify the sequences which are potentially coeliac-predictive. However, the challenge of also identifying all coeliac-predictive sequences complexifies the problem of overfitting. Overfitting of this model would consist of identifying a set of coeliac-predictive sequences which are only predictive of single samples which happen to be from CeD patients, rather than being coeliac-predictive in general. It is possible that there is some redundancy in the classification model, whereby samples could be correctly classified using a smaller number of sequences. Removing these would reduce the problem of overfitting; however, it also potentially removes sequences which are coeliac-predictive. Since all sequences considered have been experimentally shown to bind gluten epitopes, it is likely that some samples will contain multiple sequences. In an attempt to mitigate this problem, we have used completely independent training and testing datasets, and demonstrated that the results observed in training generalise to the testing data. By identifying the sequences which are most of interest (in Table 4 and Table 5), we attempt to provide further insight into the sequences which we consider to be highly likely to be coeliac-predictive. However, further validation using larger datasets is required to demonstrate that the sequences selected are truly coeliac-predictive.

The training and testing set use different methods of sequencing and amplification. However, the congruence in accuracy of the results, despite the relatively small sample size, illustrates that the sequences identified are important, with broad application. A greater number of gluten-specific sequences are seen in RNA, suggesting that this approach may be superior. Larger datasets, with HLA-typed samples, will be required in order to validate these results further and confirm their diagnostic use or potential as biomarkers. When considering larger datasets, we expect to be able to refine the model parameters further, obtaining more robust estimates of sensitivity and specificity, and to determine the specifications, such as the sequencing depth, required in order to achieve this accuracy. Diagnostic approaches for CeD which use MHC tetramer-based assays [50,51] have shown early promise; however, they can be technically challenging. The techniques we used to collect repertoire data are less complex and the bioinformatic steps can be automated, making it possible to incorporate them into existing clinical pathways. An emerging diagnostic approach, using whole blood cytokine release assays [52], has shown potential in a small number of samples. However, the approach proposed here has the advantage that it is able to identify the TCR sequences on which a diagnosis could be based. This improves our understanding of the immune response involved in CeD, and therefore has the potential to provide the basis for novel treatments or disease monitoring in the future.

We have only considered full length CDR3 amino acid sequences here. However, some shorter motifs that are constituent parts of CDR3 sequences, such as those illustrated using logo plots, have been shown to be associated with coeliac disease [36,53], and these may provide additional biomarkers and will form the basis of future analyses. The logo plots in Figure 3B show that TCR-β has some conserved motifs which are consistent with those previously published [25]. This study considers only the CDR3; inclusion of the CDR1 and CDR2 may help improve specificity in cases where a particular CDR3 is seen in both CeD patients and controls. Additionally, this study is limited to exact matches of CDR3s. Extending this to allow for more complex relationships, such as single base mismatches or partial string matches, could improve sensitivity. However, there are a large number of ways in which two CDR3 sequences could be considered similar, and exploring all these options for a large number of sequences is computationally expensive. To explore these possibilities fully will require large computing resources and efficient algorithms, but may produce greater accuracy.

Both training and testing datasets are samples from duodenal biopsies; however, collecting these biopsies is an invasive procedure. Identification of these coeliac-specific sequences in blood samples has the potential to provide a non-invasive test, particularly if there is also no requirement for the patients to consume gluten. Further experiments are required in order to demonstrate the utility of this method in blood samples. It is likely that the sequences identified here would be present at lower frequencies in blood samples. Therefore, it will be important to establish the sequencing depth and the number of cells from blood that would need to be sequenced in order to obtain an accurate diagnosis. However, there is also the potential for the development of assays which test specifically for the sequences identified here at very low frequencies in blood samples.

Single cell sequencing would provide invaluable validation and would determine whether any of the TCR-α and TCR-β sequences identified here are present in paired chains. It would also provide insight into whether the gluten-specific TCRs occur predominantly within specific T-cell populations, and provide sufficient information for gluten-specific T cells to be produced and tested experimentally.

## 5. Conclusions

In summary, we have developed the basis of a novel diagnostic testing method for coeliac disease, by cataloguing all the previously published gluten-specific TCR-α and TCR-β CDR3 sequences, then by developing and optimising an interpretable machine learning method, with empirically derived thresholds for candidate CDR3 sequence frequency. This approach has produced a list of 10 TCR-α and 10 TCR-β sequences that are present in CeD patients and not in non-CeD controls, in both the training and testing datasets. We consider these sequences to be those which are of most interest for future research, having the most potential for diagnosis or use as biomarkers for CeD.

The use of this model has the potential to remove the subjectivity from the diagnosis of coeliac disease and also remove the requirement to consume gluten prior to diagnosis. This is an important contribution, since consuming gluten is painful for coeliac patients to the extent that some will chose to forego diagnosis rather than consume gluten. In order to explore the full diagnostic potential of this model, further validation on larger cohorts is required. Additional future work of particular interest is to determine whether these TCR sequences can be detected in peripheral blood samples of CeD patients. This will require further sample collection and TCR sequencing experiments, but has the potential to remove the invasive aspect CeD diagnosis.

## Figures and Tables

**Table 1:** Summaries of number of productive sequences, clones, and starting cell numbers within each dataset: TCR-α training and testing, and TCR-β training and testing.

	TCR-α		TCR-β	
	Cohort 1: Training	Cohort 2: Testing	Cohort 1: Training	Cohort 2: Testing
Number of samples	20 (12 CeD, 8 controls)	15 (9 CeD, 6 controls)	20 (12 CeD, 8 controls)	15 (9 CeD, 6 controls)
Mean number of productive sequences per sample (min–max)	284,888 (57,629–1,280,631)	587,482 (19,649–1,276,321)	228,422 (33,421–797,688)	627,303 (787,765–1,693,681)
Mean number of clones per sample (min–max)	5203 (1404–16,376)	1104 (217–3747)	4076 (830–12,048)	2231 (362–8613)
Mean number of CD4^+^ T cells sorted per sample (min–max)	25,603 (6054–104,187)	8000 (estimated)	25,603 (6054–104,187)	8000 (estimated)

**Table 2:** Papers which publish gluten-specific TCR CDR3 sequences and the peptides used to identify them.

Publication	Chains (Number of Sequences)	Paired	T-Cell Gluten Epitopes								
			DQ8-glia-α1	DQ8-glia-γ1b	DQ2.2-glut-L1	DQ2.5-glia-α1a	DQ2.5-glia-α2	DQ2.5-glia-ω1	DQ2.5-glia-ω2	DQ2.5-glia-γ2	DQ2.5-hor-3
Broughton [37]	TCR-α (9) TCR-β (9)	Yes	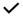								
Dahal-Koirala [38]	TCR-α (11) TCR-β (11)	Yes									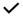
Dahal-Koirala [39]	TCR-α (10) TCR-β (10)	Yes					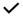		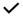		
Petersen [40]	TCR-α (18) TCR-β (18)	Yes	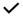								
Risnes [26]	TCR-α (14) TCR-β (8)	No	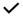	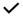		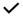	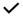	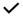	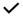		
Hardy [41]	TCR-α (211) TCR-β (216)	No						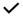	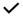		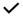
Gunnarsen [42]	TCR-α (9) TCR-β (9)	Yes				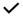	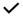	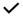	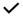		
Cook [43]	TCR-β (26)	No				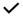	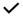				
Ting [44]	TCR-α (23) TCR-β (23)	Yes			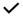						
Qiao [45]	TCR-α (29) TCR-β (29)	Yes					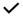				
Qiao [46]	TCR-β (6)	No				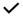	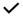			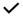	
Dahal-Koirala [47]	TCR-α (7) TCR-β (7)	Yes				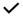		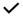			
Hardy [48]	TCR-β (21)	No				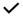	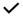				
Petersen [49]	TCR-α (20) TCR-β (20)	Yes				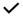	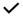				
Han [36]	TCR-β (437) TCR-δ (194)	No				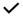	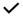				
Christophersen [35]	TCR-α (10) TCR-β (7)	Yes				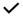	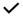	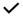	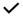		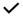
	Total TCR-α: 357 Total TCR-β: 799										

**Table 3:** Leave-one-out cross-validation results.

Loci	Sensitivity	Specificity	Balanced Accuracy	Optimal Threshold Values
TCR-α	1.0	0.75	0.875	0.2–0.8
TCR-β	0.92	0.25	0.585	0.3
TCR-α and TCR-β	1.0	0.75	0.875	0.1–0.8

**Table 4:** TCR-α sequences present at ranked-frequency higher than the threshold in both training and testing datasets, prioritised by the number of coeliac samples in which they were observed in the testing and training datasets. Both training and testing datasets include CeD patients on gluten-containing and gluten-free diets. None of these sequences were present in any control samples. = indicates sequences with equal rankings.

Rank	Sequence	Training: Number of CeD Samples	Validation: Number of Cross-Validation Folds	Testing: Number of CeD Samples
1	AYRSEQGAQKLV	2	20	3
2	GDGGATNKL	6	19	2
3	RDLYNFNKF	2	19	2
4	GDDTGFQKL	4	20	1
5	IVFNDYKLS	3	20	1
6=	AVGETGANNLF	2	19	1
6=	SITGYAL	2	19	1
8=	DINAGNML	1	19	1
8=	RGSAGGTSYGKL	1	19	1
8=	AVEGGSNYKLT	1	19	1

**Table 5:** TCR-β sequences present at ranked-frequency higher than the threshold in both training and testing datasets, prioritised by the number of coeliac samples in which they were observed in the testing and training datasets. Both training and testing datasets include CeD patients on gluten-containing and gluten-free diets. None of these sequences were present in any control samples. = indicates sequences with equal rankings.

Rank	Sequence	Training: Number of CeD Samples	Validation: Number of Cross-Validation Folds	Testing: Number of CeD Samples
1	IRSTDT	4	20	3
2	VRFTDT	1	19	3
3	SFRTTDTQ	4	20	1
4	ASSIRATDTQY	3	20	1
5=	IRTTDT	2	20	1
5=	LRSTDT	2	19	1
5=	LRATDT	2	19	1
5=	SASDSLNTEAF	2	19	1
5=	SLRWTDTQ	2	20	1
10	ASSLTVTDTQY	1	19	1

## Data Availability

Cohort 1 data are original data and are available at ImmPort (https://www.immport.org, accessed on 26 October 2023) under study accession SDY2369. Cohort 2 data are a publicly available dataset which can be found in SRA (https://www.ncbi.nlm.nih.gov/sra, accessed on 1 June 2022), project ID: PRJNA678347.

## Supplementary Materials

The following supporting information can be downloaded at: https://www.mdpi.com/article/10.3390/biom13121707/s1, Figure S1: T-cell receptor structure, Figure S2: Training TCR- α summary, Figure S3: Training TCR-β summary, Figure S4: Testing TCR-α summary, Figure S5: Testing TCR-β summary, Figure S6: CD4^+^ T cell sorting gate, Figure S7: Logo plots of TCR-α sequences included in the model, Figure S8: Logo plots of TCR-β sequences included in the model; Table S1: Patient characteristics for cohort 1, Table S2: All previously published sequences.
