# Supplementary material for: An Interpretable Classification Model Using Gluten-Specific TCR Sequences Shows Diagnostic Potential in Coeliac Disease
Source: Biomolecules. 2023 Nov 25;13(12):1707. doi: 10.3390/biom13121707 (PMC10742135; doi:10.3390/biom13121707)
Supplement: Supplementary file 1 [file biomolecules-13-01707-s001.zip › biomolecules-2671386-supplementary.pdf]

biomolecules

Supplementary Material

1 Supplementary Figures

1.1 **Supplementary Figure S1:** T cell receptors have 2 chains, according to which T cells can be categorized. Each TCR chain has a constant region and a variable region made up of V (D) and J segments

During T cell maturation/selection, T cells undergo somatic recombination, where the TCR gene segments are rearranged, leaving a few select gene segments, while the rest are deleted. During gene rearrangement the V-J segments are joined in TRA genes. In TRB genes, the D-J segments are joined first, followed by the joining of the selected V gene to the rearranged DJ segments. In the case of TRA and TRB genes, additional junctional diversity is generated at each joining step through the addition of random, non-templated nucleotides.

The complementarity-determining region 3 (CDR3) is crucial in antigen recognition. CDR3 of T cells is highly variable, and is located at the V(D)J junction for each TCR chain.

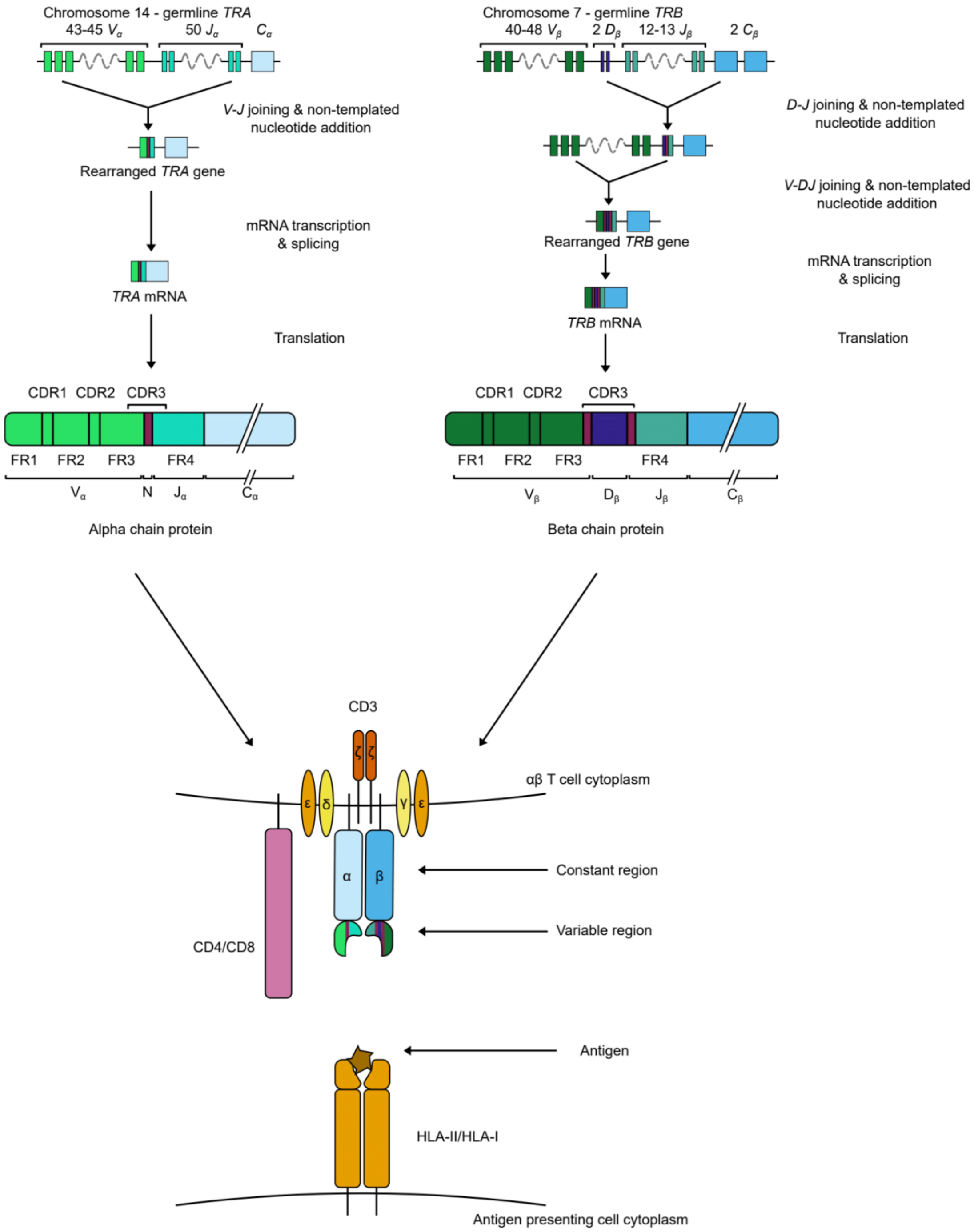
Chromosome 14 - germline *TRA*
43-45 V_α
50 J_α
C_α
V-J joining & non-templated nucleotide addition
Rearranged *TRA* gene
mRNA transcription & splicing
TRA mRNA
Translation
CDR1 CDR2 CDR3
FR1 FR2 FR3 FR4
V_α N J_α C_α
Alpha chain protein
Chromosome 7 - germline *TRB*
40-48 V_β
2 D_β
12-13 J_β
2 C_β
D-J joining & non-templated nucleotide addition
V-DJ joining & non-templated nucleotide addition
Rearranged *TRB* gene
mRNA transcription & splicing
TRB mRNA
Translation
CDR1 CDR2 CDR3
FR1 FR2 FR3 FR4
V_β D_β J_β C_β
Beta chain protein
CD3
ζ ζ
ε δ γ ε
αβ T cell cytoplasm
α β
Constant region
Variable region
CD4/CD8
Antigen
HLA-II/HLA-I
Antigen presenting cell cytoplasm

1.2 Supplementary Figure S2: Training TCR-α dataset summary

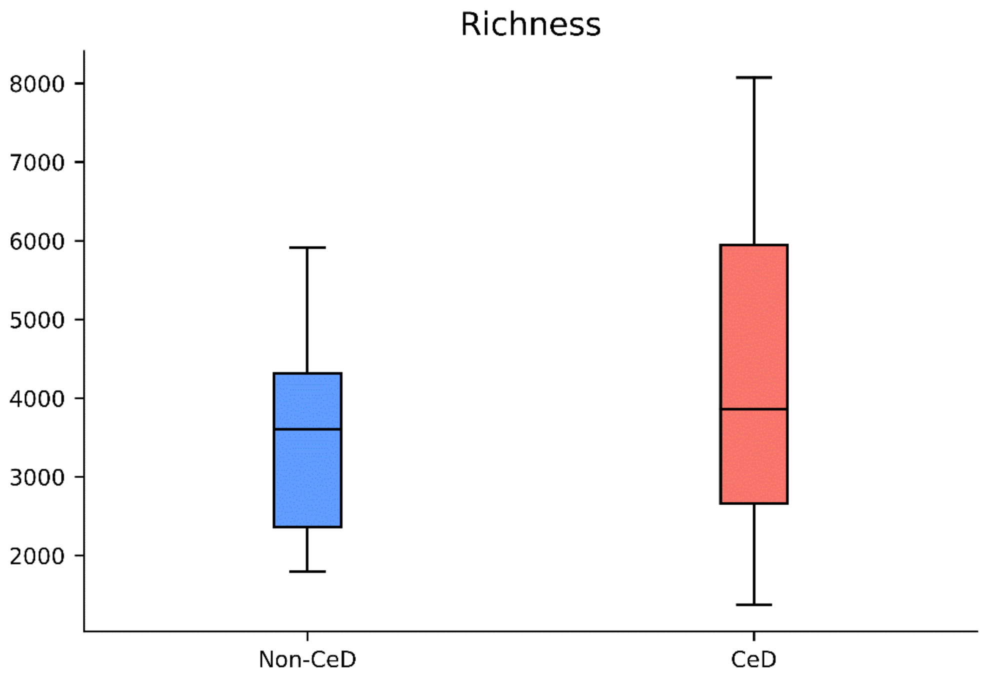

a. Boxplot indicating richness (number of unique sequences) in TCR-α repertoire samples from CeD patients and non-CeD individuals. No significant difference in mean between the two groups was found (adjusted p = 1.00, unadjusted p = 0.402).

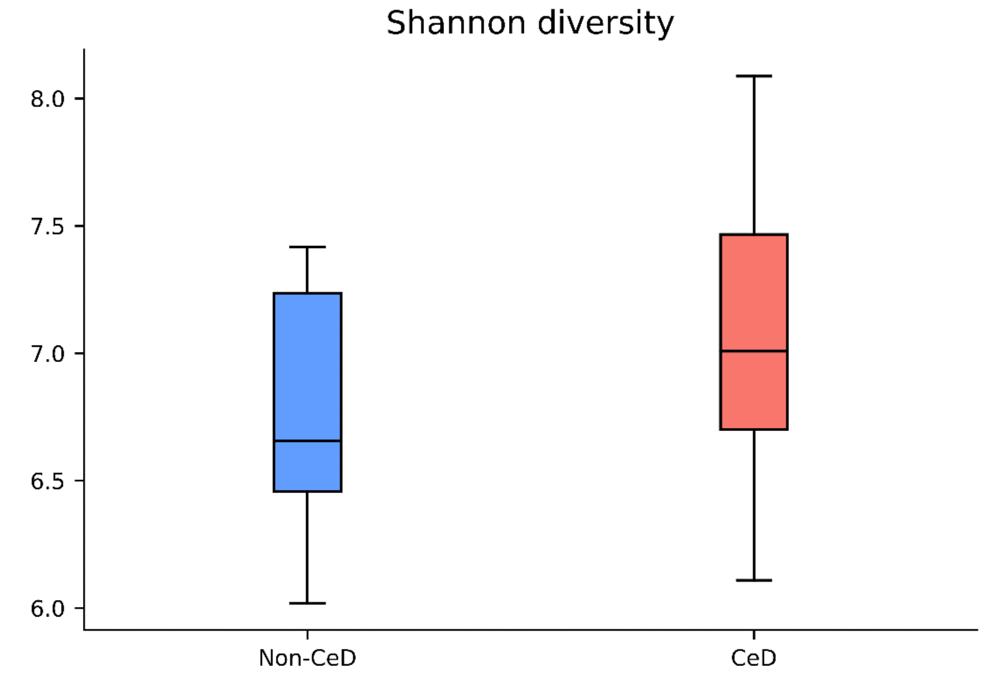

b. Boxplot indicating Shannon diversity in TCR-α repertoire samples from CeD patients and non-CeD individuals. No significant difference in mean between the two groups was found (adjusted p = 0.663, unadjusted p = 0.221).

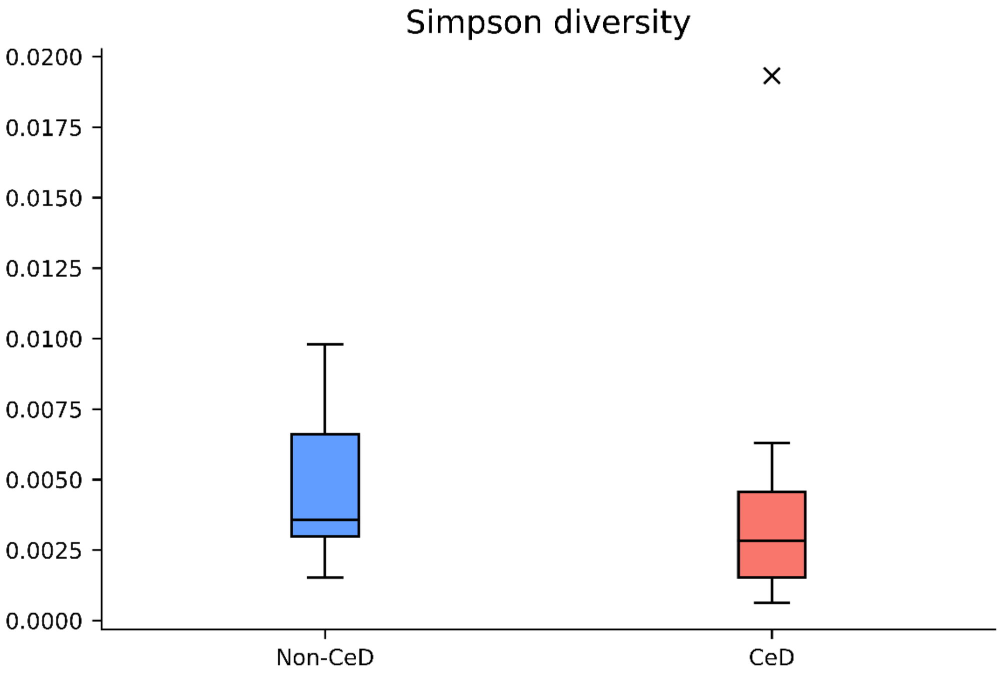

c. Boxplot indicating Simpson diversity in TCR-α repertoire samples from CeD patients and non-CeD individuals. No significant difference in mean between the two groups was found (adjusted p = 1.00, unadjusted p = 0.776).

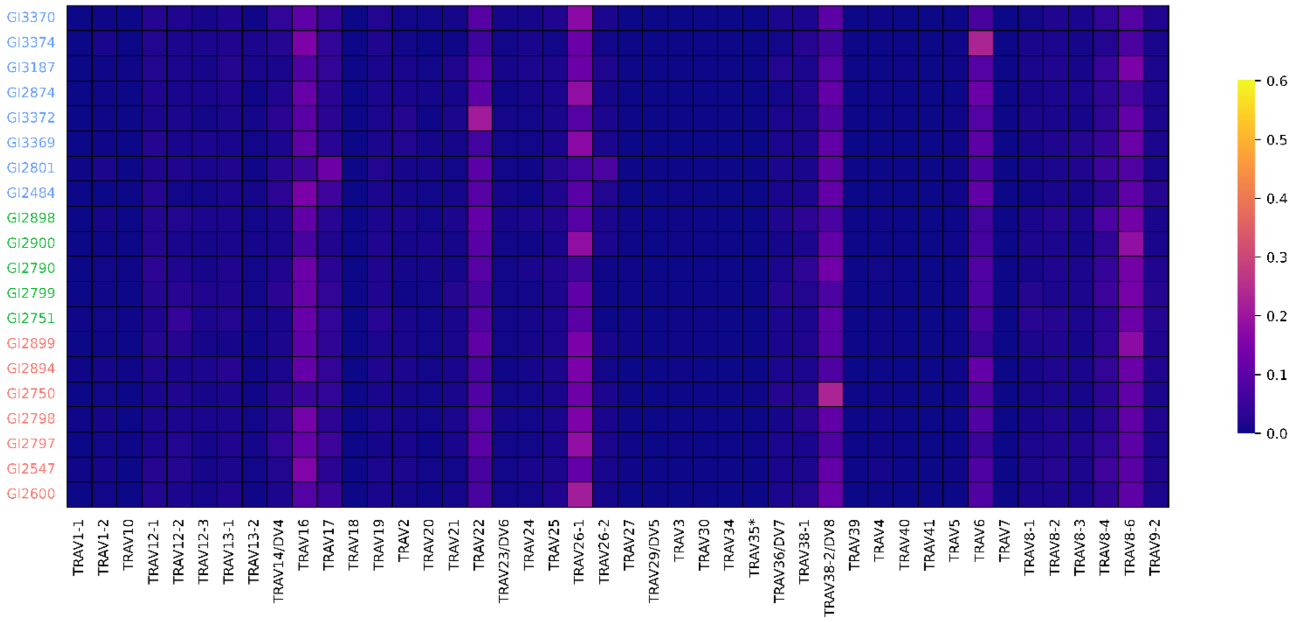

d. Heatmap showing proportions of TCR-α repertoire samples that use each V segment. TRAV35 shows significantly different mean usage between repertoire samples from CeD patients and non-CeD individuals, indicated by * (adjusted p = 0.0149, unadjusted p = 0.000338).

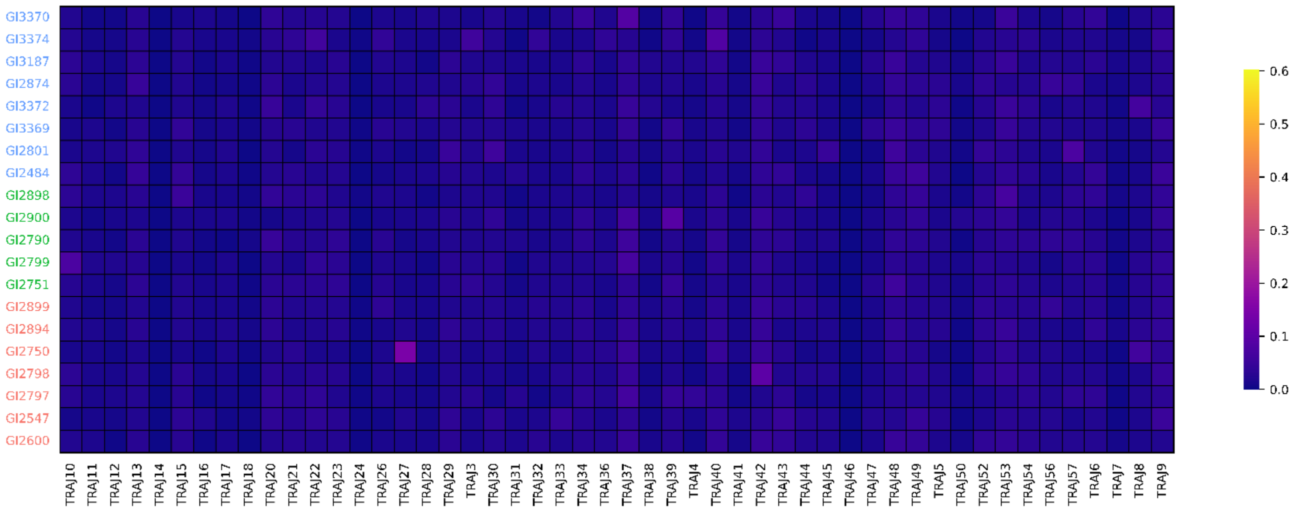

e. Heatmap showing proportions of TCR-α repertoire samples that use each J segment. No J segment shows significantly different mean usage between repertoire samples from CeD patients and non-CeD individuals.

1.3 Supplementary Figure S3: Training TCR-β dataset summary.

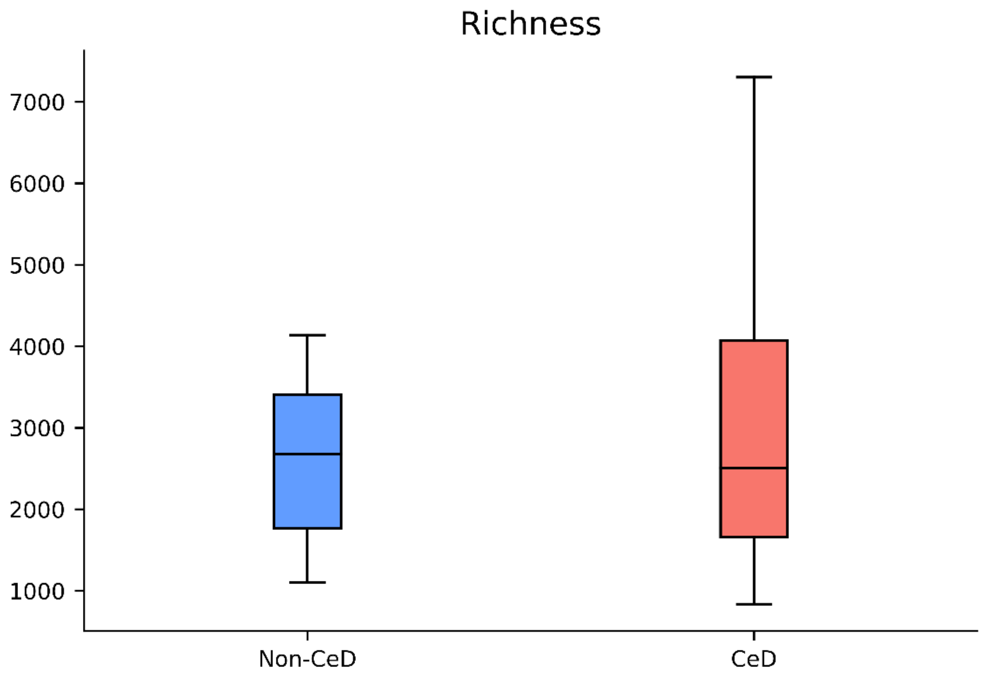

a. Boxplot indicating richness (number of unique sequences) in TCR-β repertoire samples from CeD patients and non-CeD individuals. No significant difference in mean between the two groups was found (adjusted p =1.00, unadjusted p =0.483).

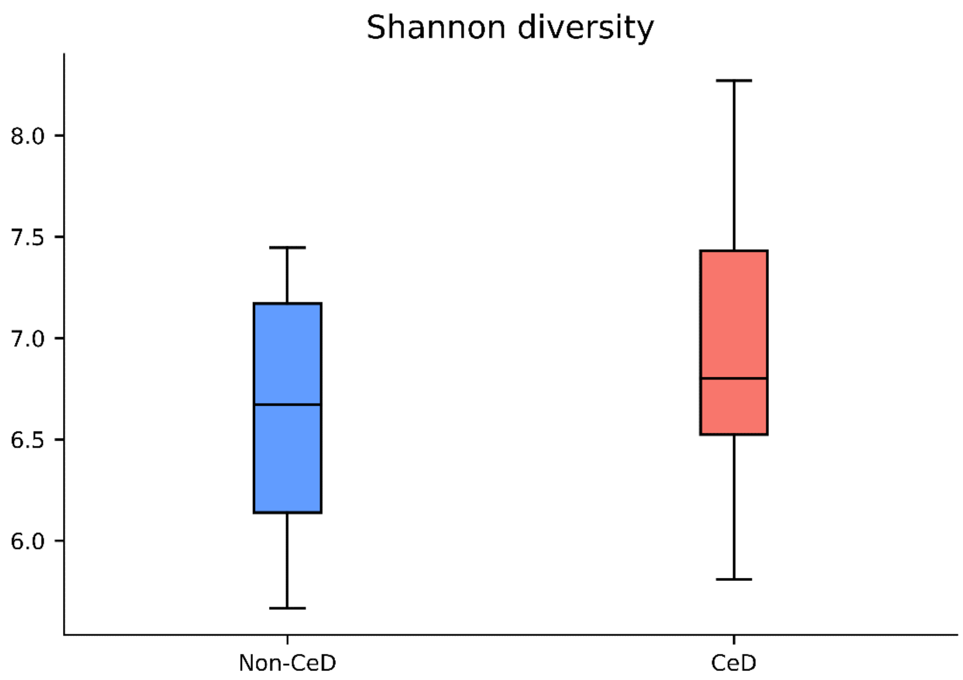

b. Boxplot indicating Shannon diversity in TCR-β repertoire samples from CeD patients and non-CeD individuals. No significant difference in mean between the two groups was found (adjusted p = 1.00, unadjusted p = 0.382).

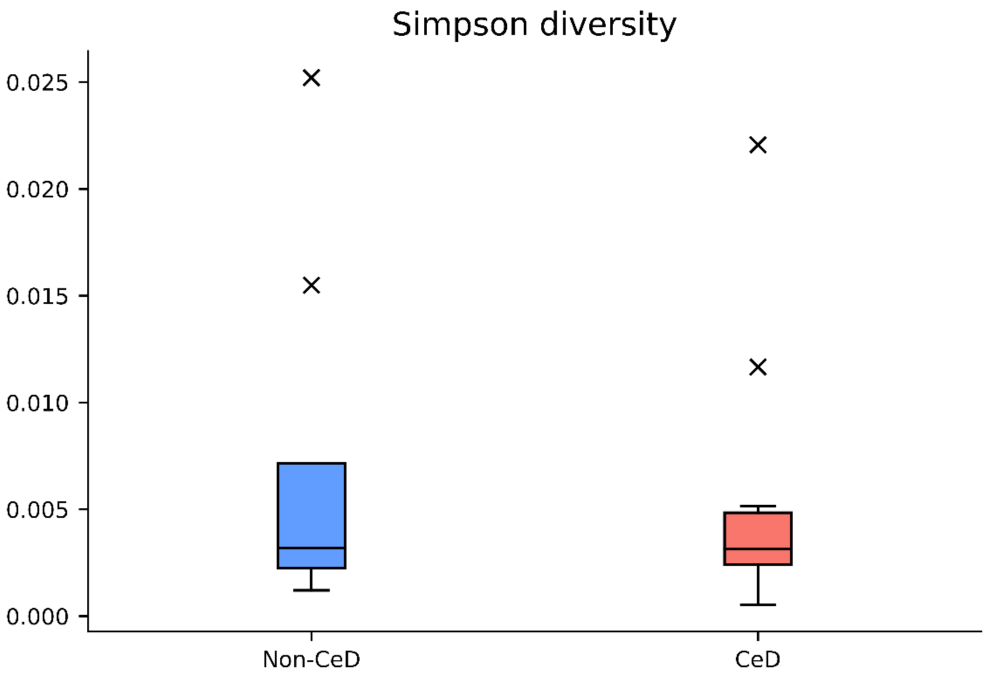

c. Boxplot indicating Simpson diversity in TCR-β repertoire samples from CeD patients and non-CeD individuals. No significant difference in mean between the two groups was found (adjusted p =1.00, unadjusted p = 0.568).

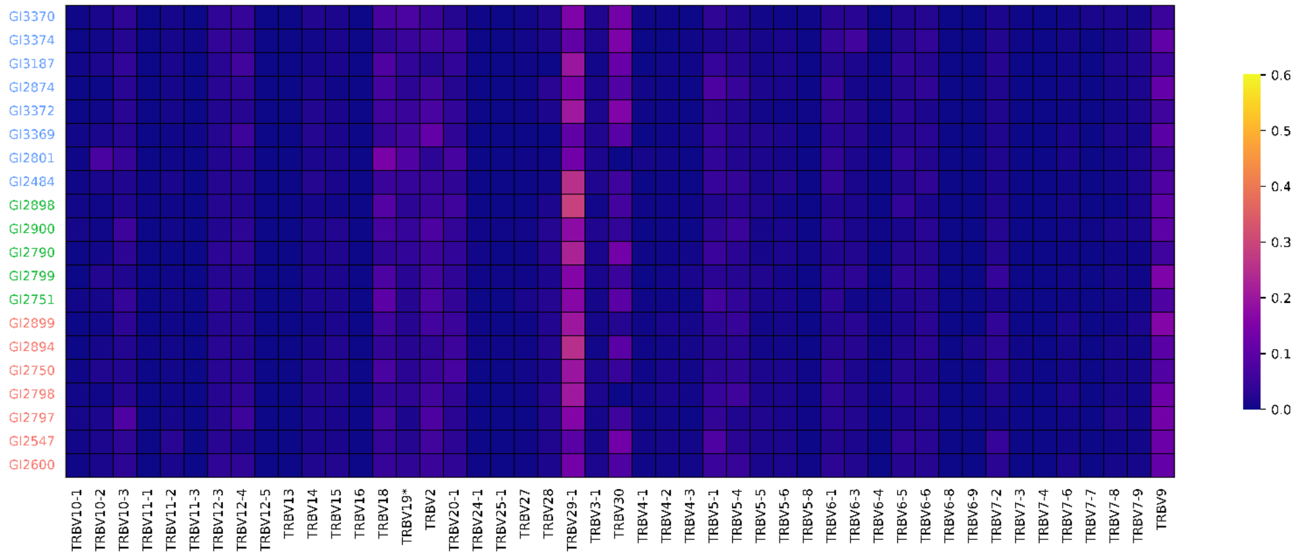

d. Heatmap showing proportions of TCR-β repertoire samples that use each V segment. We observed significantly lower usage of segment TRBV19 (adjusted p = 0.0340, unadjusted p = 0.000724), indicated by *, in CeD patient samples compared to non-CeD samples.

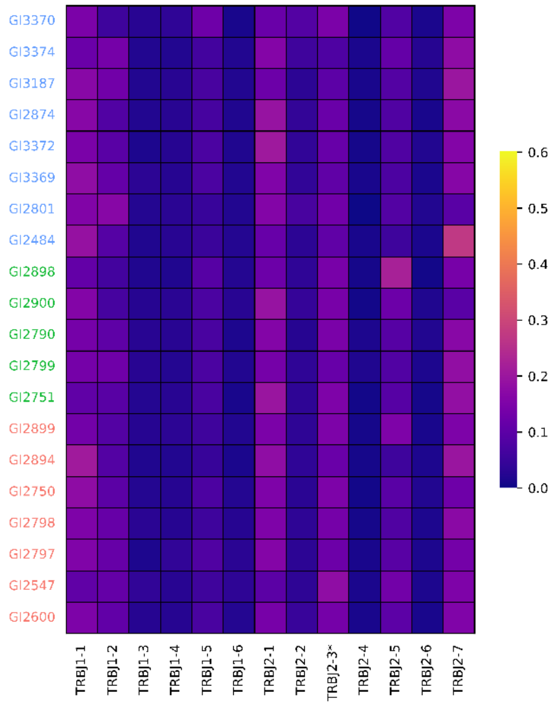

e. Heatmap showing proportions of TCR-β repertoire samples that use each J segment. We observed significantly higher usage of segment TRBJ2-3 (adjusted p = 0.0422, unadjusted p = 0.00325), indicated by *, in CeD patient samples compared to non-CeD samples, see S2h.

1.4 Supplementary Figure S4: Testing TCR-α dataset summary.

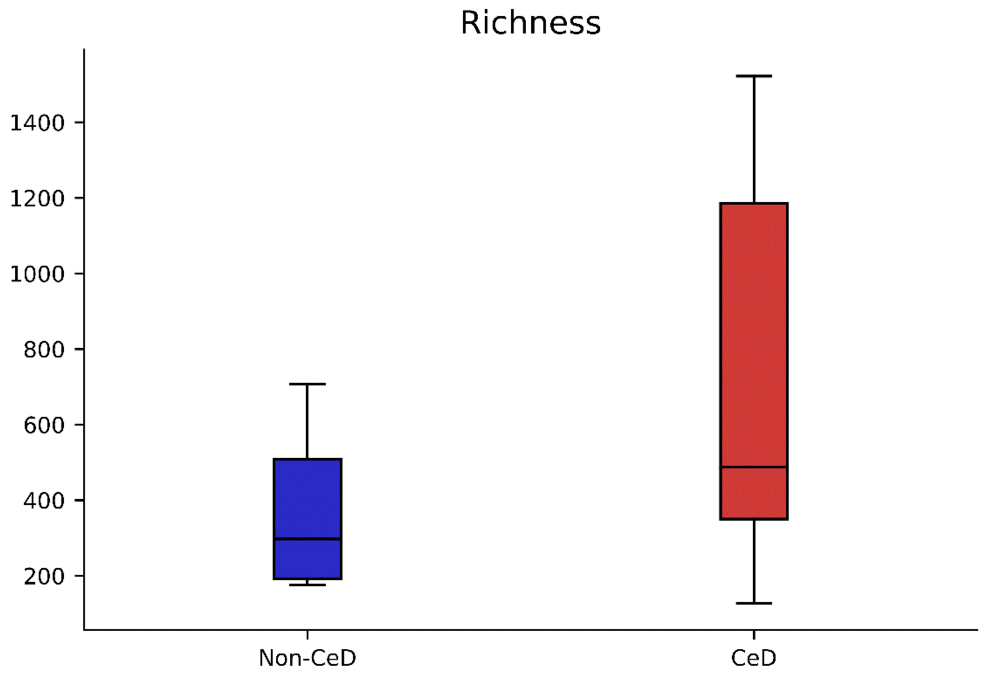

a. Boxplot indicating richness (number of unique sequences) in TCR-α repertoire samples from CeD patients and non-CeD individuals. No significant difference in mean between the two groups was found (adjusted p = 0.464, unadjusted p =0.155).

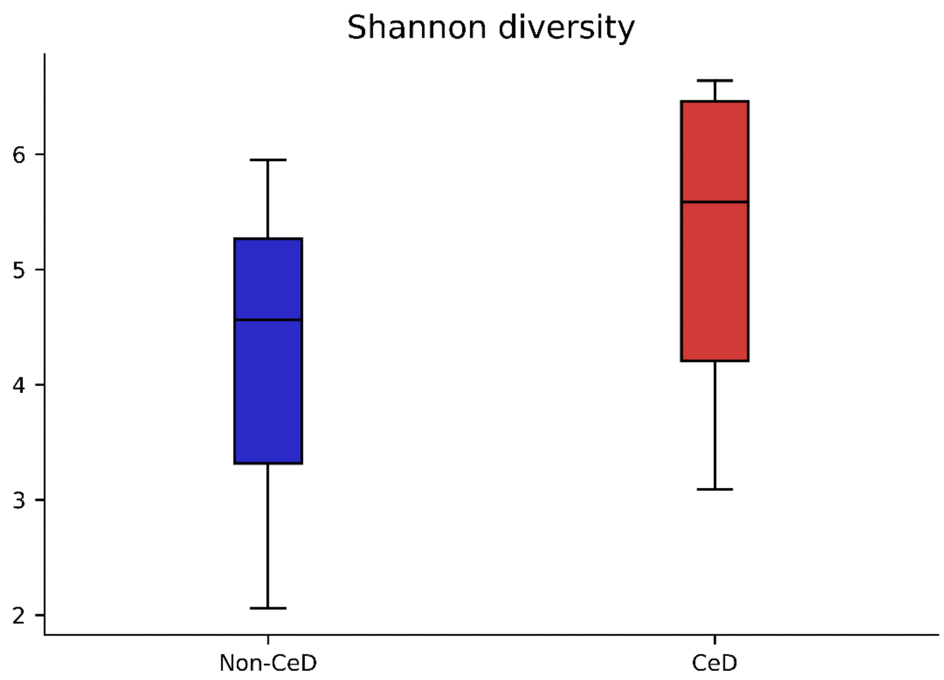

b. Boxplot indicating Shannon diversity in TCR-α repertoire samples from CeD patients and non-CeD individuals. No significant difference in mean between the two groups was found (adjusted p =0.475, unadjusted p =0.158).

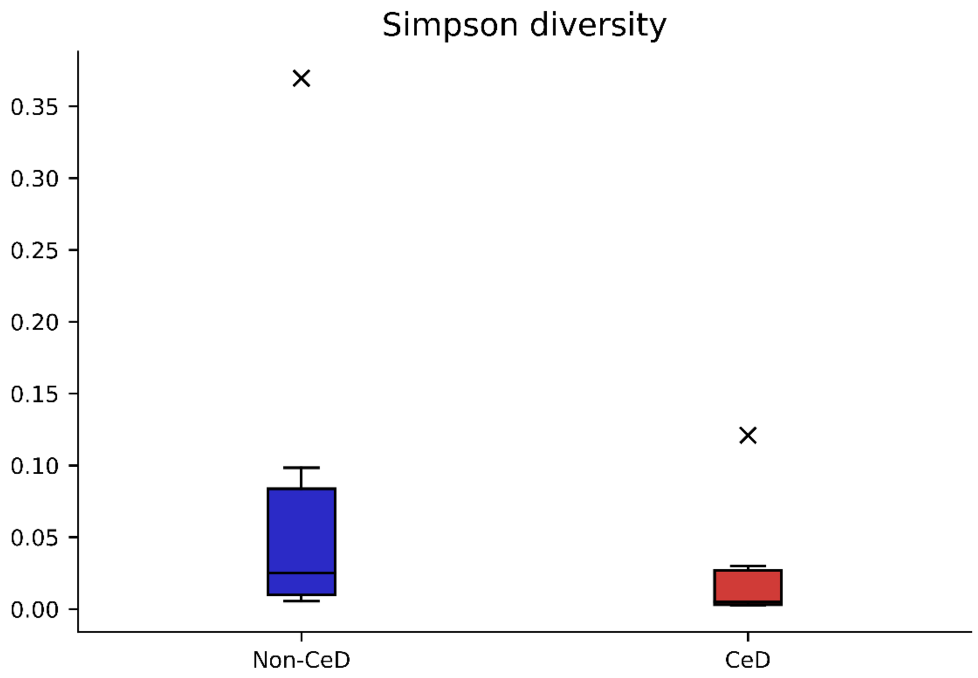

c. Boxplot indicating Simpson diversity in TCR-α repertoire samples from CeD patients and non-CeD individuals. No significant difference in mean between the two groups was found (adjusted p =0.598, unadjusted p =0.199).

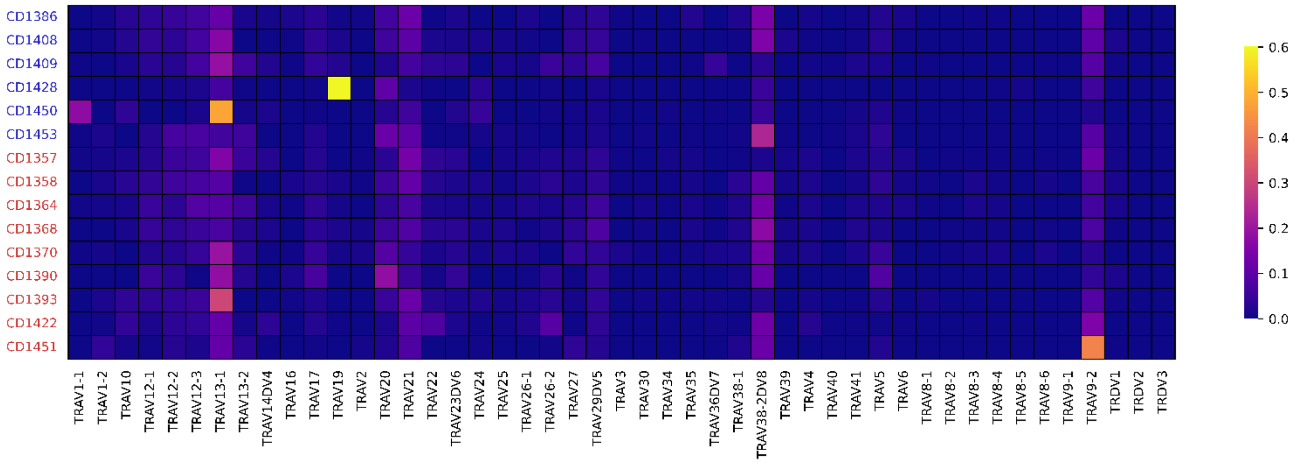

d. Heatmap showing proportions of TCR-α repertoire samples that use each V segment. No V segment shows significantly different mean usage between repertoire samples from CeD patients and non-CeD individuals.

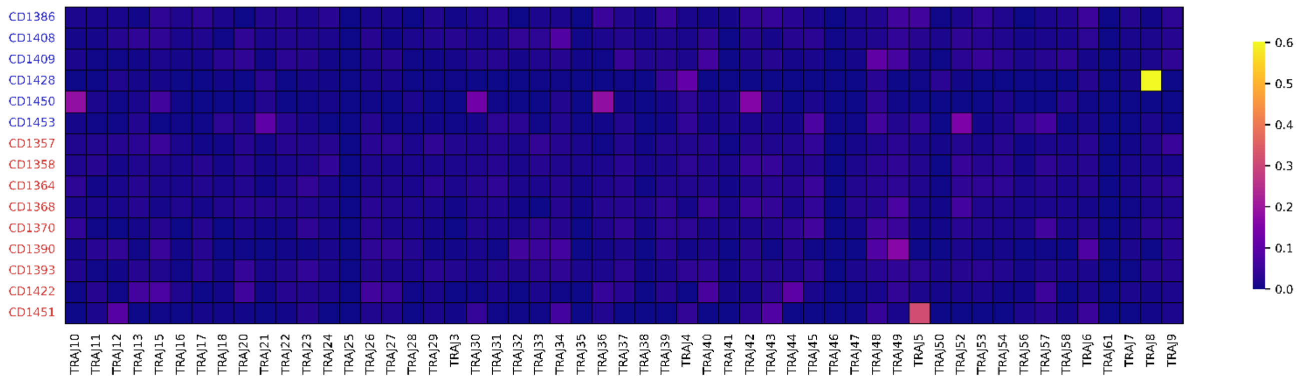

e. Heatmap showing proportions of TCR-α repertoire samples that use each J segment. No V segment shows significantly different mean usage between repertoire samples from CeD patients and non-CeD individuals.

1.5 Supplementary Figure S5: Testing TCR-β dataset summary.

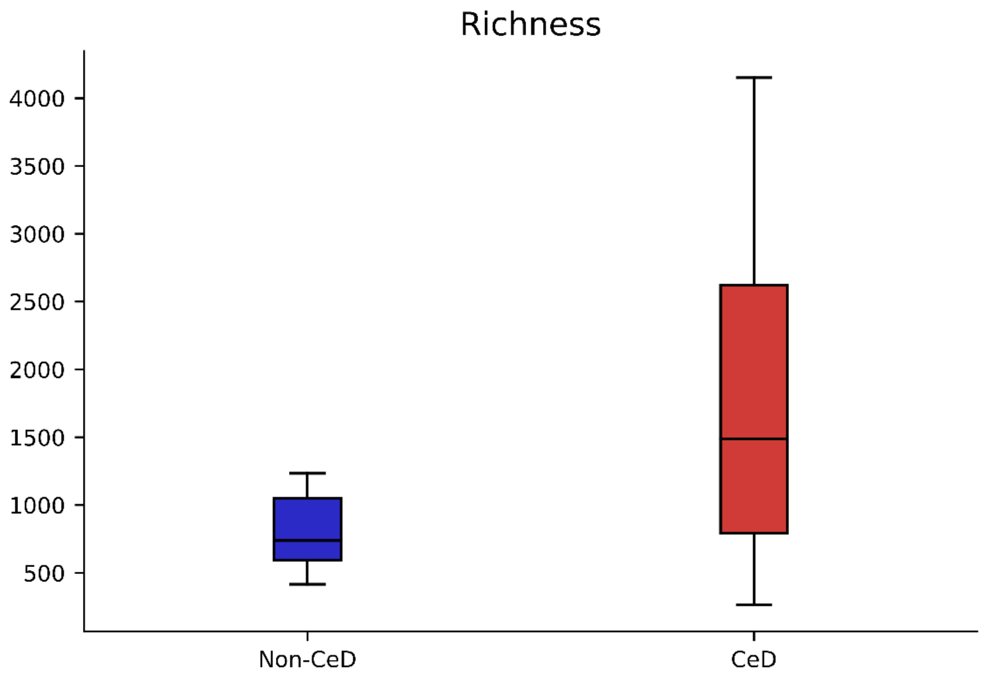

a. Boxplot indicating richness (number of unique sequences) in TCR-β repertoire samples from CeD patients and non-CeD individuals. No significant difference in mean between the two groups was found (adjusted p =0.313, unadjusted p =0.104).

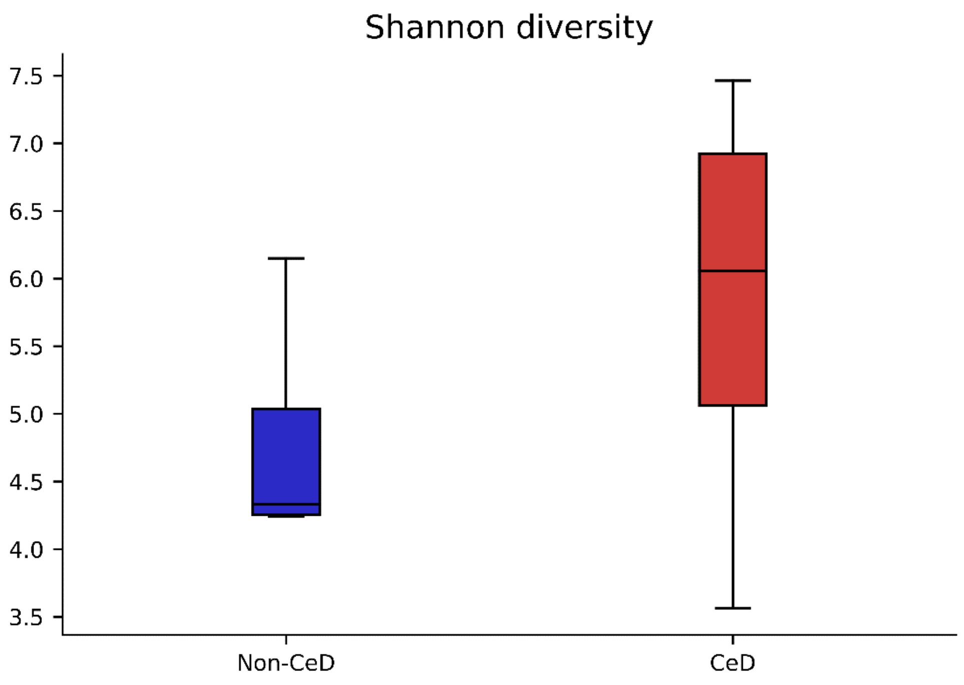

b. Boxplot indicating Shannon diversity in TCR-β repertoire samples from repertoire samples from CeD patients and non-CeD individuals. No significant difference in mean between the two groups was found (adjusted p =0.294, unadjusted p = 0.0981)

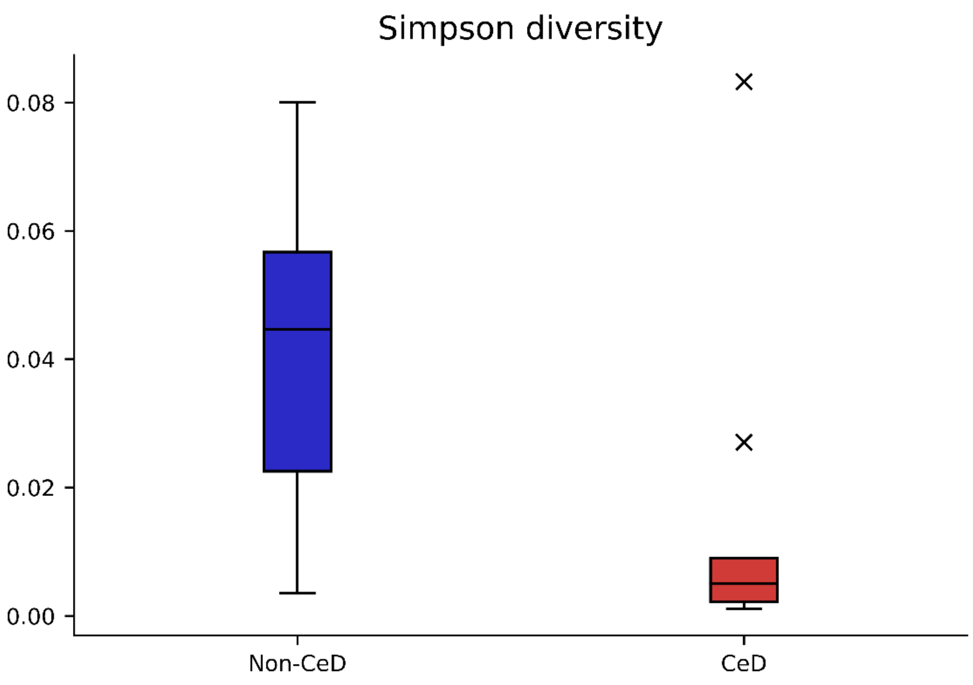

c. Boxplot indicating Simpson diversity in TCR-β repertoire samples from CeD patients and non-CeD individuals. No significant difference in mean between the two groups was found (adjusted p = 0.272, unadjusted p =0.0908).

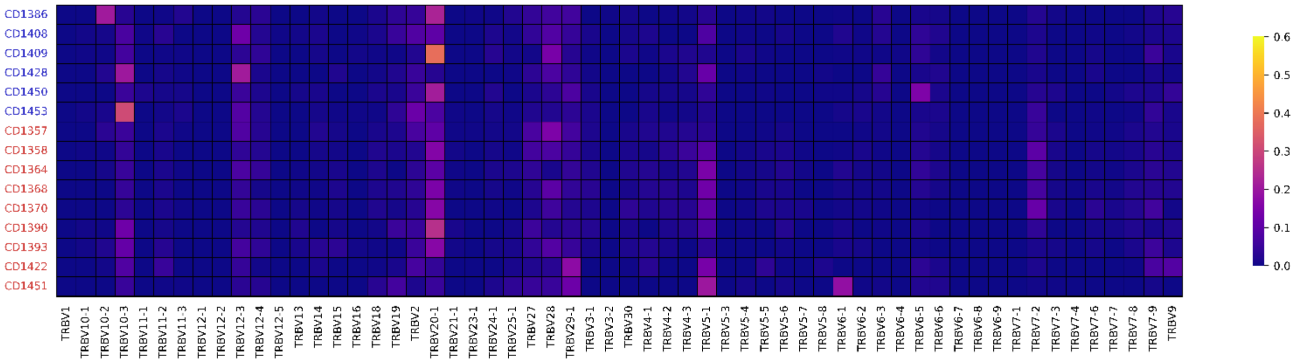

d. Heatmap showing proportions of TCR-β repertoire samples that use each V segment. No V segment shows significantly different mean usage between repertoire samples from CeD patients and non-CeD individuals.

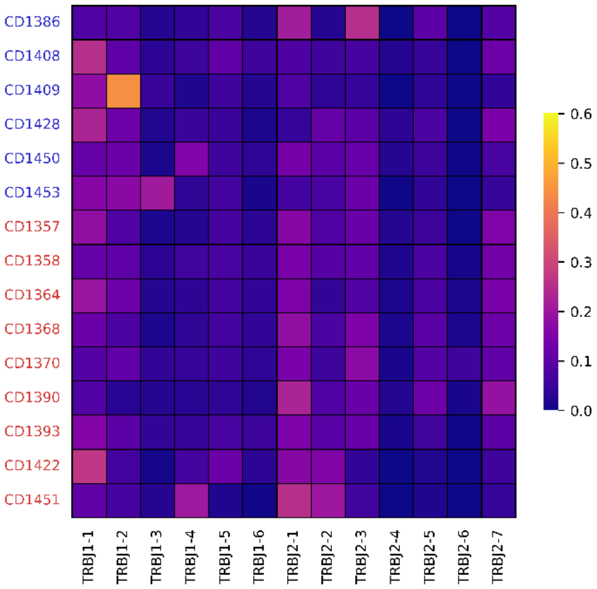

e. Heatmap showing proportions of TCR-β repertoire samples that use each J segment. No V segment shows significantly different mean usage between repertoire samples from CeD patients and non-CeD individuals.

1.6 Supplementary Figure S6: CD4+ T cell sorting gate

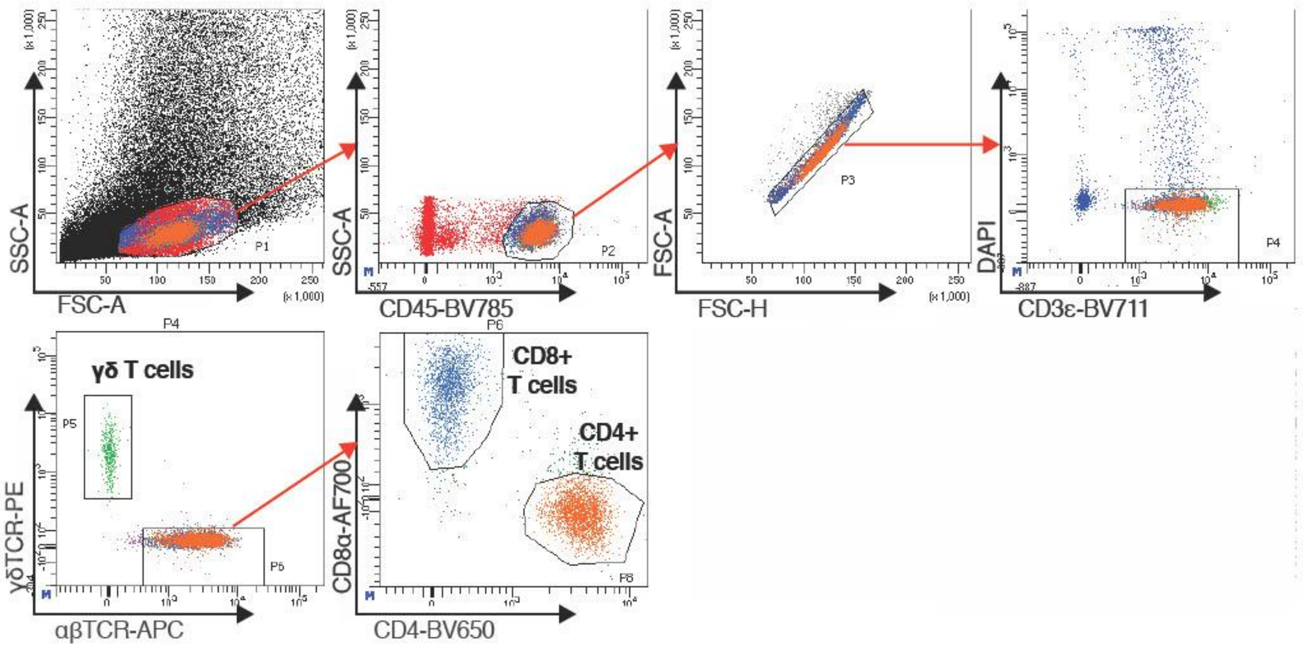

Representative gating strategy for FACS-sorting of intestinal T cell subsets (CD4+, CD8+ and γδ+).

1.7 Supplementary Figure S7: Logo plots of TCR-alpha sequences included in the model

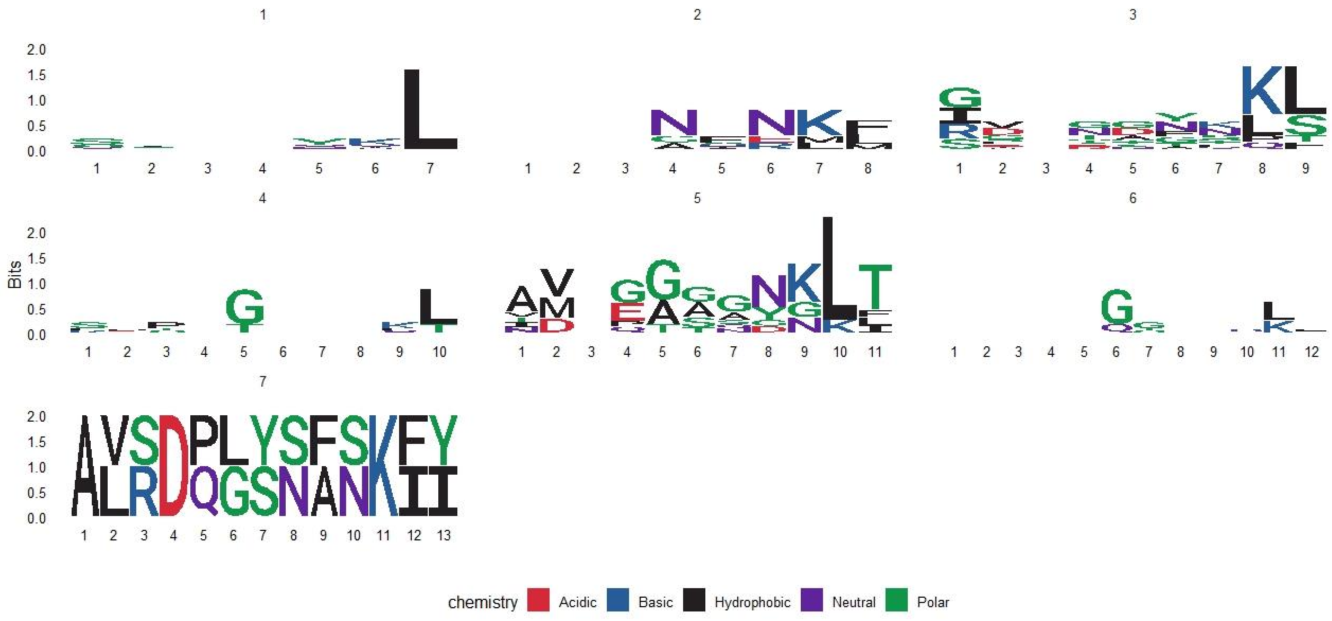

1.8 Supplementary Figure S8: Logo plots of TCR-beta sequences included in the model

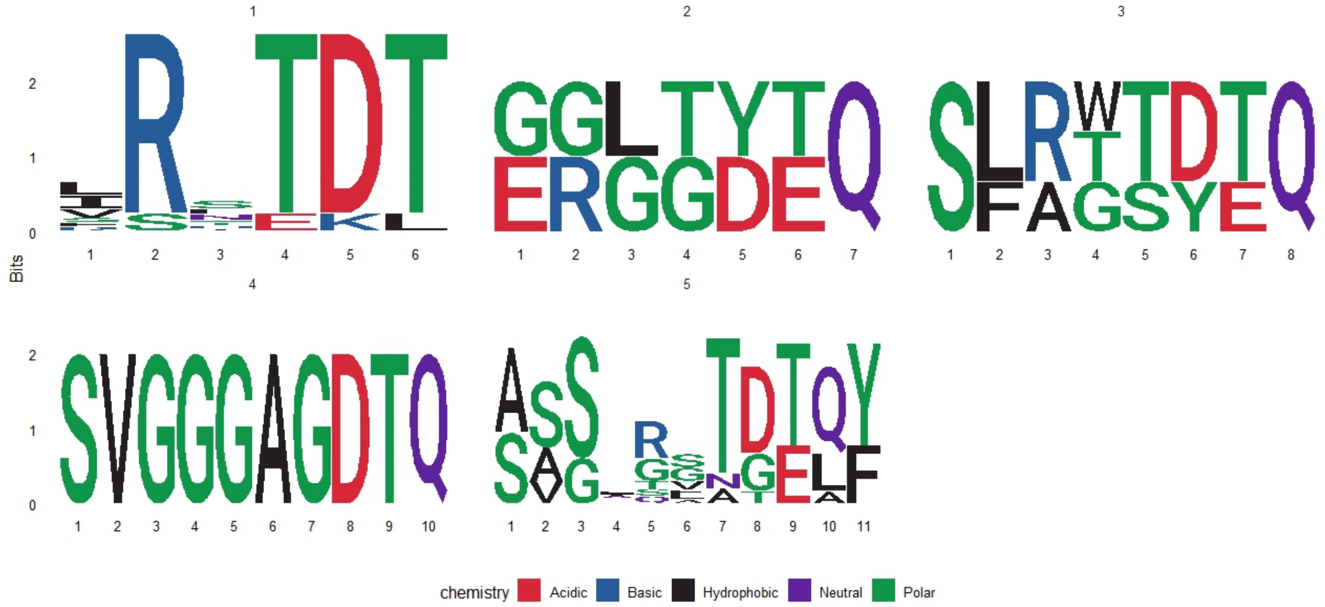

2 Supplementary Tables

2.1 Supplementary Table S1

	Age	Sex	Cohort	IgA-TTG	Marsh classification	HLA type
GI2547	19	F	CeD	356	Marsh 3	NA
GI2600	26	F	CeD	867	Marsh 3	NA
GI2750	33	F	CeD	831	Marsh 3	DQ2
GI2797	45	F	CeD	1301	Marsh 3	DQ2
GI2798	27	F	CeD	789	Marsh 3	DQ2
GI2894	20	F	CeD	1621	Marsh 3	DQ2
GI2899	62	F	CeD	879	Marsh 3	DQ2
GI2751	30	F	CeD-GFD	16	Marsh 1	DQ2
GI2799	41	M	CeD-GFD	296	Marsh 1	DQ2
GI2790	30	F	CeD-GFD	17	Marsh 1	DQ2
GI2898	57	M	CeD-GFD	<1.9	Marsh 0	DQ2
GI2900	60	F	CeD-GFD	83	Marsh 0	DQ8
GI2484	63	F	Control	Not tested	Marsh 0	NA
GI2801	23	M	Control	Not tested	Marsh 0	NA
GI2874	33	F	Control	Not tested	Marsh 0	DQ2
GI3187	25	F	Control	Not tested	Marsh 0	NA
GI3369	25	F	Control	<1.9	Marsh 0	NA
GI3370	26	F	Control	Not tested	Marsh 0	DQ8
GI3372	68	F	Control	<1.9	Marsh 0	DQ2
GI3374	17	F	Control	<1.9	Marsh 0	DQ8

Supplementary Table S1: Patient characteristics for cohort 1 (training data set). All patients on a gluten free diet did not consume gluten for at least 6 months.

2.2 Supplementary Table S2

Sequence	Publication	Loci	Pair (If provided)
YYCILRDGRGGADGLTFG	Broughton; 2012; Biased	alpha	CASSVAVSAGTYEQYFG
YYCILRDSRAQKLVFG	Broughton; 2012; Biased	alpha	CASSAGTSGEYEQYFG
YYCILRDRSNQFYFG	Broughton; 2012; Biased	alpha	CASSTTPGTGTETQYFG
YFCAYRSARGARLMFG	Broughton; 2012; Biased	alpha	CAS SVAVSAGTYEQ YFG
YFCAGGSSNTGKLIFG	Broughton; 2012; Biased	alpha	CAS SQDIRNTGEL FFG
YFCATDFPGTASKLTFG	Broughton; 2012; Biased	alpha	CAS SEALPGRSGNTI YFG
YFCAVGETGANNLFFG	Broughton; 2012; Biased	alpha	CAS SEARRYNEQ FGP
YLCAVQASGGSYIPTFG	Broughton; 2012; Biased	alpha	CAS SNRGLGTDTQ YFG
YFCAASAYPGNQFYFG	Broughton; 2012; Biased	alpha	CAS SVYDGRGETQ YFG
CASSVAVSAGTYEQYFG	Broughton; 2012; Biased	beta	YYC ILRDGRGGADGLT FG
CASSAGTSGEYEQYFG	Broughton; 2012; Biased	beta	YYC ILRDSRAQKLV FG
CASSTTPGTGTETQYFG	Broughton; 2012; Biased	beta	YYC ILRDRSNQFY FG
CASSVAVSAGTYEQYFG	Broughton; 2012; Biased	beta	YFC AYRSARGARLM FG
CASSQDIRNTGELFFG	Broughton; 2012; Biased	beta	YFC AGGSSNTGKLI FG
CASSEALPGRSGNTIYFG	Broughton; 2012; Biased	beta	YFC ATDFPGTASKLT FG
CASSEARRYNEQFGP	Broughton; 2012; Biased	beta	YFC AVGETGANNLF FG
CASSNRGLGTDTQYFG	Broughton; 2012; Biased	beta	YLC AVQASGGSYIPT FG
CASSVYDGRGETQYFG	Broughton; 2012; Biased	beta	YFC AASAYPGNQFY FG
AVEAGSGGGADGLT	Dahal-Koirala, 2019, immune	alpha	SASRTSGRAGDEQF
AGQDYKYI	Dahal-Koirala, 2019, immune	alpha	SASRTGGTDTQY
AVIYQGGKLI	Dahal-Koirala, 2019, immune	alpha	NO RESULTS
AVRVEYNFNKFY	Dahal-Koirala, 2019, immune	alpha	ASSQEGLADSYNEQF
CLVGDSM#GATNKLIF	Dahal-Koirala, 2019, immune	alpha	CSASRTSGRAGDEQFF
AVESSWGKLQ	Dahal-Koirala, 2019, immune	alpha	SAARTSGRAGDTQY
AVEAGSGGGADGLT	Dahal-Koirala, 2019, immune	alpha	SASRTSGRAGDEQF
AVGGEGFKTI	Dahal-Koirala, 2019, immune	alpha	ASSEVGGPSHEQY
ALENQGGKLI	Dahal-Koirala, 2019, immune	alpha	SAARTSGRAGDTQY
ALVARGYGNNRLA	Dahal-Koirala, 2019, immune	alpha	SAARTSGRAGDTQY
AVGGTDKLI	Dahal-Koirala, 2019, immune	alpha	SASRTSGRAGDEQF
AMSLTGANSKLT	Dahal-Koirala, 2019, immune	alpha	ASSHIAGAGTDTQY
SASRTSGRAGDEQF	Dahal-Koirala, 2019, immune	beta	AVEAGSGGGADGLT
SASRTGGTDTQY	Dahal-Koirala, 2019, immune	beta	AGQDYKYI
ASSQEGLADSYNEQF	Dahal-Koirala, 2019, immune	beta	AVRVEYNFNKFY
CSASRTSGRAGDEQFF	Dahal-Koirala, 2019, immune	beta	CLVGDSM#GATNKLIF
SAARTSGRAGDTQY	Dahal-Koirala, 2019, immune	beta	AVESSWGKLQ
SASRTSGRAGDEQF	Dahal-Koirala, 2019, immune	beta	AVEAGSGGGADGLT
ASSEVGGPSHEQY	Dahal-Koirala, 2019, immune	beta	AVGGEGFKTI
SAARTSGRAGDTQY	Dahal-Koirala, 2019, immune	beta	ALVARGYGNNRLA|ALENQGGKLI

SASRTSGRAGDEQF	Dahal-Koirala, 2019, immune	beta	AVGGTDKLI
ASSHIAGAGTDTQY	Dahal-Koirala, 2019, immune	beta	AMSLTGANSKLT
IVYGGFKTI	Dahal-Koirala; 2016;TCR	alpha	ASSLRSTDTQY
LVGHGSSNTGKLI	Dahal-Koirala; 2016;TCR	alpha	ASSLRWGDGGKL
IEYNFNKFY	Dahal-Koirala; 2016;TCR	alpha	ASRPVAG
ALSELSIQGAQKLV	Dahal-Koirala; 2016;TCR	alpha	ASSIVDRGGETQY
AGDSGYALN	Dahal-Koirala; 2016;TCR	alpha	ATSSRMGGDTQY
IVPANTGGFKTI	Dahal-Koirala; 2016;TCR	alpha	ASSFRSTDTQY
LVGGGADGLT	Dahal-Koirala; 2016;TCR	alpha	ASSVRTLDTGELF
ALSESGANSKLT	Dahal-Koirala; 2016;TCR	alpha	ASSLRAWETQY
AVRDPLYNFNKFY	Dahal-Koirala; 2016;TCR	alpha	ASSQGGDRGESEAF
VVNSWAGNQFY	Dahal-Koirala; 2016;TCR	alpha	ASSLGYGVSTGELF
ASSLRSTDTQY	Dahal-Koirala; 2016;TCR	beta	IVYGGFKTI
ASSLRWGDGGKL	Dahal-Koirala; 2016;TCR	beta	LVGHGSSNTGKLI
ASRPVAG	Dahal-Koirala; 2016;TCR	beta	IEYNFNKFY
ASSIVDRGGETQY	Dahal-Koirala; 2016;TCR	beta	ALSELSIQGAQKLV
ATSSRMGGDTQY	Dahal-Koirala; 2016;TCR	beta	AGDSGYALN
ASSFRSTDTQY	Dahal-Koirala; 2016;TCR	beta	IVPANTGGFKTI
ASSVRTLDTGELF	Dahal-Koirala; 2016;TCR	beta	LVGGGADGLT
ASSLRAWETQY	Dahal-Koirala; 2016;TCR	beta	ALSESGANSKLT
ASSQGGDRGESEAF	Dahal-Koirala; 2016;TCR	beta	AVRDPLYNFNKFY
ASSLGYGVSTGELF	Dahal-Koirala; 2016;TCR	beta	VVNSWAGNQFY
CILRDGRGGADGLTF	Peterson; 2015; determinants	alpha	CASSVAVSAGTYEQYF
CILRDSRAQKLVF	Peterson; 2015; determinants	alpha	CASSAGTSGEYEQYF
CILRDSRAQKLVF	Peterson; 2015; determinants	alpha	CASSVGVAGEYEQYF
CILRDRSNQFYF	Peterson; 2015; determinants	alpha	CASSTTPGTGTETQYF
CILRDDRGGKLIF	Peterson; 2015; determinants	alpha	CASSAGPQAGTQYF
CILRGRDNYGQNFVF	Peterson; 2015; determinants	alpha	CASSVESGISNNEQFF
CIVTGRGSTLGRLYF	Peterson; 2015; determinants	alpha	CASSVEVSGNTIYF
CIVHGRQTGANNLFF	Peterson; 2015; determinants	alpha	CASSVEVSGNTIYF
CIVRGRNAGMLTF	Peterson; 2015; determinants	alpha	CASSETLGNEQYF

CIVRANLVGNTGKLIF	Peterson; 2015; determinants	alpha	CASSEGPDLAKNIQY
CAVGETGANNLFF	Peterson; 2015; determinants	alpha	CASSEARRYNEQFF
CAVGDTGANNLFF	Peterson; 2015; determinants	alpha	CASSELRRYNEQFF
CAVVNTGTASKLTF	Peterson; 2015; determinants	alpha	CASSEGPTGRAGNTIYF
CATDFPGTASKLTF	Peterson; 2015; determinants	alpha	CASSEALPGRSGNTIYF
CLVGDGISGGYNKLIF	Peterson; 2015; determinants	alpha	CASSEGLAVAKNIQYF
CAVMVQGAQKLVF	Peterson; 2015; determinants	alpha	CSASRAGDFSTDTQYF
CAYRSARGARLMF	Peterson; 2015; determinants	alpha	CASSVAVSAGTYEQYF
CAGGSSNTGKLIF	Peterson; 2015; determinants	alpha	CASSQDIRNTGELFF
CASSVAVSAGTYEQYF	Peterson; 2015; determinants	beta	CILRDGRGGADGLTF
CASSAGTSGEYEQYF	Peterson; 2015; determinants	beta	CILRDSRAQKLVF
CASSVGVAGEYEQYF	Peterson; 2015; determinants	beta	CILRDSRAQKLVF
CASSTTPGTGTETQYF	Peterson; 2015; determinants	beta	CILRDRSNQFYF
CASSAGPQAGTQYF	Peterson; 2015; determinants	beta	CILRDDRGGKLIF
CASSVESGISNNEQFF	Peterson; 2015; determinants	beta	CILRGRDNYGQNFVF
CASSVEVSGNTIYF	Peterson; 2015; determinants	beta	CIVTGRGSTLGRLYF
CASSVEVSGNTIYF	Peterson; 2015; determinants	beta	CIVHGRQTGANNLFF
CASSETLGNEQYF	Peterson; 2015; determinants	beta	CIVRGRNAGMLTF
CASSEGPDLAKNIQY	Peterson; 2015; determinants	beta	CIVRANLVGNTGKLIF
CASSEARRYNEQFF	Peterson; 2015; determinants	beta	CAVGETGANNLFF
CASSELRRYNEQFF	Peterson; 2015; determinants	beta	CAVGDTGANNLFF

CASSEGPTGRAGNTIYF	Peterson; 2015; determinants	beta	CAVVNTGTASKLTF
CASSEALPGRSGNTIYF	Peterson; 2015; determinants	beta	CATDFPGTASKLTF
CASSEGLAVAKNIQYF	Peterson; 2015; determinants	beta	CLVGDGISGGYNKLIF
CSASRAGDFSTDTQYF	Peterson; 2015; determinants	beta	CAVMVQGAQKLVF
CASSVAVSAGTYEQYF	Peterson; 2015; determinants	beta	CAYRSARGARLMF
CASSQDIRNTGELFF	Peterson; 2015; determinants	beta	CAGGSSNTGKLIF
LGDDTGFQKLV	Rinses; 2018; disease-driving	alpha	
IAFNDYKLS	Rinses; 2018; disease-driving	alpha	
ISFNDYKLS	Rinses; 2018; disease-driving	alpha	
IVFNDYKLS	Rinses; 2018; disease-driving	alpha	
IAYNDYKLS	Rinses; 2018; disease-driving	alpha	
ISYNDYKLS	Rinses; 2018; disease-driving	alpha	
IVYNDYKLS	Rinses; 2018; disease-driving	alpha	
LVGGAGGYNKLI	Rinses; 2018; disease-driving	alpha	
LVGGSGGYNKLI	Rinses; 2018; disease-driving	alpha	
AMIEAAGNKLT	Rinses; 2018; disease-driving	alpha	
AMLEAAGNKLT	Rinses; 2018; disease-driving	alpha	
AMSEAAGNKLT	Rinses; 2018; disease-driving	alpha	
AMIQAAGNKLT	Rinses; 2018; disease-driving	alpha	
AMLQAAGNKLT	Rinses; 2018; disease-driving	alpha	
AMQEAAGNKLT	Rinses; 2018; disease-driving	alpha	
ASSxRxTDTQY	Rinses; 2018; disease-driving	beta	
SAGQGGTGELF	Rinses; 2018; disease-driving	beta	
SVGQGGTGELF	Rinses; 2018; disease-driving	beta	
SAGAGGTGELF	Rinses; 2018; disease-driving	beta	
SVGAGGTGELF	Rinses; 2018; disease-driving	beta	
SAGQGGSGELF	Rinses; 2018; disease-driving	beta	
SVGQGGSGELF	Rinses; 2018; disease-driving	beta	
SAGAGGSGELF	Rinses; 2018; disease-driving	beta	
SVGAGGSGELF	Rinses; 2018; disease-driving	beta	
LGQGVAGEL	Hardy; 2019; Characterisation	beta	
GGTGAGETQ	Hardy; 2019; Characterisation	beta	

SHIAGAGTDTQ	Hardy; 2019; Characterisation	beta	
SFGSPHNEQ	Hardy; 2019; Characterisation	beta	
KESGLADVNEQ	Hardy; 2019; Characterisation	beta	
SLRGKGGHEQ	Hardy; 2019; Characterisation	beta	
SLEITYRWTDTQ	Hardy; 2019; Characterisation	beta	
STQWGIDEKL	Hardy; 2019; Characterisation	beta	
SESDDRVTDTQ	Hardy; 2019; Characterisation	beta	
SGQGDNEQ	Hardy; 2019; Characterisation	beta	
SLSLRNSGNTI	Hardy; 2019; Characterisation	beta	
REQGWTEA	Hardy; 2019; Characterisation	beta	
GREQGWTEA	Hardy; 2019; Characterisation	beta	
NRGPDYGY	Hardy; 2019; Characterisation	beta	
TRDYRSNQPQ	Hardy; 2019; Characterisation	beta	
TRTYRYNEQ	Hardy; 2019; Characterisation	beta	
TTSWDEQ	Hardy; 2019; Characterisation	beta	
EGGNYGY	Hardy; 2019; Characterisation	beta	
SEVAGNTI	Hardy; 2019; Characterisation	beta	
NEVAGNTI	Hardy; 2019; Characterisation	beta	
SRTPGQGAYEQ	Hardy; 2019; Characterisation	beta	
SRFPSGIGVSEQ	Hardy; 2019; Characterisation	beta	
SRTPGRGAYEQ	Hardy; 2019; Characterisation	beta	

SPTTSEGRIQ	Hardy; 2019; Characterisation	beta	
SPTASEGRIQ	Hardy; 2019; Characterisation	beta	
SQVYNFSPL	Hardy; 2019; Characterisation	beta	
SQVVAYEQ	Hardy; 2019; Characterisation	beta	
SQVYMYEQ	Hardy; 2019; Characterisation	beta	
SRTSENTGEL	Hardy; 2019; Characterisation	beta	
SHLAGAGTDTQ	Hardy; 2019; Characterisation	beta	
SQVLFYEQ	Hardy; 2019; Characterisation	beta	
SRTPW	Hardy; 2019; Characterisation	beta	
SFGIDTQ	Hardy; 2019; Characterisation	beta	
GGDAKNIQ	Hardy; 2019; Characterisation	beta	
SAQWVEQ	Hardy; 2019; Characterisation	beta	
SLWPPGQGSETQ	Hardy; 2019; Characterisation	beta	
GTSGRVITDTQ	Hardy; 2019; Characterisation	beta	
SQDLAWTLGGRGHNEQ	Hardy; 2019; Characterisation	beta	
SPLGLAGAYEQ	Hardy; 2019; Characterisation	beta	
SITGSGEL	Hardy; 2019; Characterisation	beta	
KTPAPTYEQ	Hardy; 2019; Characterisation	beta	
SREWETQ	Hardy; 2019; Characterisation	beta	
GRLTDTQ	Hardy; 2019; Characterisation	beta	
SIVTGGWGIQ	Hardy; 2019; Characterisation	beta	

SRTSGRAGDEQ	Hardy; 2019; Characterisation	beta	
ARTSGRAGDEQ	Hardy; 2019; Characterisation	beta	
SLLAGGFYEQ	Hardy; 2019; Characterisation	beta	
GKLGLDTQ	Hardy; 2019; Characterisation	beta	
SLAKQGVYEQ	Hardy; 2019; Characterisation	beta	
SFWSQDTI	Hardy; 2019; Characterisation	beta	
EAGAYEQ	Hardy; 2019; Characterisation	beta	
RWPLTGLPFLDTQ	Hardy; 2019; Characterisation	beta	
DRLTDTQ	Hardy; 2019; Characterisation	beta	
QDRPGEQ	Hardy; 2019; Characterisation	beta	
SEAYRYEQ	Hardy; 2019; Characterisation	beta	
TRSGTIGDTQ	Hardy; 2019; Characterisation	beta	
SQVYMYEQ	Hardy; 2019; Characterisation	beta	
SVGAQGGEQ	Hardy; 2019; Characterisation	beta	
SPPDRNTEA	Hardy; 2019; Characterisation	beta	
EGGNYGY	Hardy; 2019; Characterisation	beta	
AQGWGYEQ	Hardy; 2019; Characterisation	beta	
SKQGGTATNEKL	Hardy; 2019; Characterisation	beta	
SFGQGSTEA	Hardy; 2019; Characterisation	beta	
SLQGGETQ	Hardy; 2019; Characterisation	beta	
SPPXGGAAEA	Hardy; 2019; Characterisation	beta	

HGIERGPEETQ	Hardy; 2019; Characterisation	beta	
EAGTAYAYNSPL	Hardy; 2019; Characterisation	beta	
ARTSGRAGDTQ	Hardy; 2019; Characterisation	beta	
SSLGPDQPQ	Hardy; 2019; Characterisation	beta	
GLAKNIQ	Hardy; 2019; Characterisation	beta	
SEAYRYEQ	Hardy; 2019; Characterisation	beta	
SLNVVTYNSPL	Hardy; 2019; Characterisation	beta	
SQAGGLFNSPL	Hardy; 2019; Characterisation	beta	
SFSAGTTEA	Hardy; 2019; Characterisation	beta	
VDSVTYEQ	Hardy; 2019; Characterisation	beta	
EGGGYEQ	Hardy; 2019; Characterisation	beta	
ILWGIGTGTDTQ	Hardy; 2019; Characterisation	beta	
SHIAGAGTDTQ	Hardy; 2019; Characterisation	beta	
SSGQPTTDTQ	Hardy; 2019; Characterisation	beta	
SFGGPYNEQ	Hardy; 2019; Characterisation	beta	
SWLAGGPEETQ	Hardy; 2019; Characterisation	beta	
SSDAYTGEL	Hardy; 2019; Characterisation	beta	
SPRAGASGHTGEL	Hardy; 2019; Characterisation	beta	
SIFLGIRNQPQ	Hardy; 2019; Characterisation	beta	
SEWGLQGNEQ	Hardy; 2019; Characterisation	beta	
SLGTVSNYGY	Hardy; 2019; Characterisation	beta	

RRAGWETEA	Hardy; 2019; Characterisation	beta	
ARTSGRAGDEQ	Hardy; 2019; Characterisation	beta	
SQSRGTFTHHSEA	Hardy; 2019; Characterisation	beta	
TPRGGNQPQ	Hardy; 2019; Characterisation	beta	
RVAGYNEQ	Hardy; 2019; Characterisation	beta	
TVMDSSYKL	Hardy; 2019; Characterisation	alpha	
SIVGYQL	Hardy; 2019; Characterisation	alpha	
DYGGATNKL	Hardy; 2019; Characterisation	alpha	
SDNWYGGATNKL	Hardy; 2019; Characterisation	alpha	
RAFGNEKL	Hardy; 2019; Characterisation	alpha	
NGGNYGQNF	Hardy; 2019; Characterisation	alpha	
TPRGYAL	Hardy; 2019; Characterisation	alpha	
NPGGAGGTSYGKL	Hardy; 2019; Characterisation	alpha	
IILTGGGNKL	Hardy; 2019; Characterisation	alpha	
SRTSGSARQL	Hardy; 2019; Characterisation	alpha	
SIGNYQL	Hardy; 2019; Characterisation	alpha	
NRKTSYDKV	Hardy; 2019; Characterisation	alpha	
SLTGANSKL	Hardy; 2019; Characterisation	alpha	
SADQTGANNL	Hardy; 2019; Characterisation	alpha	
KTNDYKL	Hardy; 2019; Characterisation	alpha	
SGGTGGFKT	Hardy; 2019; Characterisation	alpha	

RASEYGNKL	Hardy; 2019; Characterisation	alpha	
SARSGSNYKL	Hardy; 2019; Characterisation	alpha	
QLPMNRDDKI	Hardy; 2019; Characterisation	alpha	
GDPYTGGGNKL	Hardy; 2019; Characterisation	alpha	
EGNYGQNF	Hardy; 2019; Characterisation	alpha	
EGNTGFQKL	Hardy; 2019; Characterisation	alpha	
SANFGFGNEKL	Hardy; 2019; Characterisation	alpha	
SSVYSGGGADGL	Hardy; 2019; Characterisation	alpha	
NAPHGGSQGNL	Hardy; 2019; Characterisation	alpha	
DINAGNML	Hardy; 2019; Characterisation	alpha	
PLNTGFQKL	Hardy; 2019; Characterisation	alpha	
SGTGTASKL	Hardy; 2019; Characterisation	alpha	
KGGTNQF	Hardy; 2019; Characterisation	alpha	
SEAGYGGATNKL	Hardy; 2019; Characterisation	alpha	
YQGGKL	Hardy; 2019; Characterisation	alpha	
RLVEYGNKL	Hardy; 2019; Characterisation	alpha	
RVPNTGFQKL	Hardy; 2019; Characterisation	alpha	
RFPVYGGNQGNL	Hardy; 2019; Characterisation	alpha	
SSRSDGQKL	Hardy; 2019; Characterisation	alpha	
RPPTQGGSEKL	Hardy; 2019; Characterisation	alpha	
MNYGGGTNKL	Hardy; 2019; Characterisation	alpha	

FMPPGNQF	Hardy; 2019; Characterisation	alpha	
RTGANNL	Hardy; 2019; Characterisation	alpha	
SVRTSGTYKY	Hardy; 2019; Characterisation	alpha	
SAGTGTASKL	Hardy; 2019; Characterisation	alpha	
ENTSYGKL	Hardy; 2019; Characterisation	alpha	
MKHRGFGNKL	Hardy; 2019; Characterisation	alpha	
MKQRGFGNVL	Hardy; 2019; Characterisation	alpha	
RPYNAGNML	Hardy; 2019; Characterisation	alpha	
WNTDKL	Hardy; 2019; Characterisation	alpha	
SITGYAL	Hardy; 2019; Characterisation	alpha	
GPGTASKL	Hardy; 2019; Characterisation	alpha	
SERGSNDYKL	Hardy; 2019; Characterisation	alpha	
NPGGAGGTSYGKL	Hardy; 2019; Characterisation	alpha	
NDAGGTSYGKL	Hardy; 2019; Characterisation	alpha	
FILTGGGNKL	Hardy; 2019; Characterisation	alpha	
ETGFQKL	Hardy; 2019; Characterisation	alpha	
GYNKL	Hardy; 2019; Characterisation	alpha	
SNDYKL	Hardy; 2019; Characterisation	alpha	
RPSEYGNKL	Hardy; 2019; Characterisation	alpha	
RASEYGNKL	Hardy; 2019; Characterisation	alpha	
EKL	Hardy; 2019; Characterisation	alpha	

AQXRGGATNKL	Hardy; 2019; Characterisation	alpha	
AQLRGGATNKL	Hardy; 2019; Characterisation	alpha	
DPGGFKT	Hardy; 2019; Characterisation	alpha	
PPPVGRL	Hardy; 2019; Characterisation	alpha	
DPQGAQKL	Hardy; 2019; Characterisation	alpha	
NTGKL	Hardy; 2019; Characterisation	alpha	
RSDPPNFGNEKL	Hardy; 2019; Characterisation	alpha	
GDGGATNKL	Hardy; 2019; Characterisation	alpha	
ALDSNYQL	Hardy; 2019; Characterisation	alpha	
SALIGGGADGL	Hardy; 2019; Characterisation	alpha	
ADKL	Hardy; 2019; Characterisation	alpha	
NEGRL	Hardy; 2019; Characterisation	alpha	
PSGSSASKI	Hardy; 2019; Characterisation	alpha	
SLTGGGADGL	Hardy; 2019; Characterisation	alpha	
RWSTGTASKL	Hardy; 2019; Characterisation	alpha	
NPWKAGGSEKL	Hardy; 2019; Characterisation	alpha	
DGSTLGRL	Hardy; 2019; Characterisation	alpha	
DNDM	Hardy; 2019; Characterisation	alpha	
PNNAGNML	Hardy; 2019; Characterisation	alpha	
RLPTSGSARQL	Hardy; 2019; Characterisation	alpha	
PGPGTYKY	Hardy; 2019; Characterisation	alpha	

RLPEYGNKL	Hardy; 2019; Characterisation	alpha	
RRTSGSRL	Hardy; 2019; Characterisation	alpha	
GVNTGGFKT	Hardy; 2019; Characterisation	alpha	
TDYNARL	Hardy; 2019; Characterisation	alpha	
IQGAQKL	Hardy; 2019; Characterisation	alpha	
SEHLLRTEA	Hardy; 2019; Characterisation	beta	
SRNRDRGL	Hardy; 2019; Characterisation	beta	
SRGLDLLGEQ	Hardy; 2019; Characterisation	beta	
SRLGVGQETQ	Hardy; 2019; Characterisation	beta	
SQGWASGFGNTGEL	Hardy; 2019; Characterisation	beta	
SLGHSGTIDGY	Hardy; 2019; Characterisation	beta	
SFFQLGDTQ	Hardy; 2019; Characterisation	beta	
STNGGNYEQ	Hardy; 2019; Characterisation	beta	
SLQPHLTATYEQ	Hardy; 2019; Characterisation	beta	
SLAREYSGNTI	Hardy; 2019; Characterisation	beta	
SFFLRDGY	Hardy; 2019; Characterisation	beta	
SEANGEL	Hardy; 2019; Characterisation	beta	
KESGLADVNEQ	Hardy; 2019; Characterisation	beta	
SQWGQGEGEQ	Hardy; 2019; Characterisation	beta	
SFSASDSYEQ	Hardy; 2019; Characterisation	beta	
SLSFDYEQ	Hardy; 2019; Characterisation	beta	

SPGQGGYNEQ	Hardy; 2019; Characterisation	beta	
SLAGSYEQ	Hardy; 2019; Characterisation	beta	
SPGQGPYGY	Hardy; 2019; Characterisation	beta	
SLAGTLPNEQ	Hardy; 2019; Characterisation	beta	
SLLAGGDTQ	Hardy; 2019; Characterisation	beta	
SWAVGSNEQ	Hardy; 2019; Characterisation	beta	
GSIAGGTYEQ	Hardy; 2019; Characterisation	beta	
SPWGGGTQ	Hardy; 2019; Characterisation	beta	
SSTGLYQETQ	Hardy; 2019; Characterisation	beta	
SESDDRVTDTQ	Hardy; 2019; Characterisation	beta	
SFSPAGGGETQ	Hardy; 2019; Characterisation	beta	
SISWTYEQ	Hardy; 2019; Characterisation	beta	
GRLGPYNEQ	Hardy; 2019; Characterisation	beta	
GGASRGYGY	Hardy; 2019; Characterisation	beta	
SPGGEKL	Hardy; 2019; Characterisation	beta	
NPGGGAVG	Hardy; 2019; Characterisation	beta	
EEGADSPL	Hardy; 2019; Characterisation	beta	
SGRRGTDTQ	Hardy; 2019; Characterisation	beta	
GRPDSPL	Hardy; 2019; Characterisation	beta	
SVGPGGEQ	Hardy; 2019; Characterisation	beta	
WRSDYGY	Hardy; 2019; Characterisation	beta	

SLRGAVLAPYEQ	Hardy; 2019; Characterisation	beta	
TPIQGAGGY	Hardy; 2019; Characterisation	beta	
SSLQRHEQ	Hardy; 2019; Characterisation	beta	
SLLQRYEQ	Hardy; 2019; Characterisation	beta	
SLVGQGRSEQ	Hardy; 2019; Characterisation	beta	
RTGEWETQ	Hardy; 2019; Characterisation	beta	
SPSGGSTDTQ	Hardy; 2019; Characterisation	beta	
EFWGGNTEA	Hardy; 2019; Characterisation	beta	
RTGEWETQ	Hardy; 2019; Characterisation	beta	
SVSLGSVNTEA	Hardy; 2019; Characterisation	beta	
STLTGDYEQ	Hardy; 2019; Characterisation	beta	
SSVPGGEQ	Hardy; 2019; Characterisation	beta	
SLAGLYEQ	Hardy; 2019; Characterisation	beta	
SISAVSTDTQ	Hardy; 2019; Characterisation	beta	
SISAVSTDTQ	Hardy; 2019; Characterisation	beta	
SLRVSQPQ	Hardy; 2019; Characterisation	beta	
RRGTGDYEQ	Hardy; 2019; Characterisation	beta	
SLGQGTTQ	Hardy; 2019; Characterisation	beta	
STRTGNEQ	Hardy; 2019; Characterisation	beta	
SPSGQGEYEQ	Hardy; 2019; Characterisation	beta	
SPSGQGEYEQ	Hardy; 2019; Characterisation	beta	

SLEGTDTQ	Hardy; 2019; Characterisation	beta	
SLVGAGDGTDTQ	Hardy; 2019; Characterisation	beta	
STGDPGEL	Hardy; 2019; Characterisation	beta	
SVGGGAGDTQ	Hardy; 2019; Characterisation	beta	
SPTLGGEL	Hardy; 2019; Characterisation	beta	
YAGLPGDEQ	Hardy; 2019; Characterisation	beta	
SYAGLPGDEQ	Hardy; 2019; Characterisation	beta	
SPGTTRSGEL	Hardy; 2019; Characterisation	beta	
SQVNRHTEA	Hardy; 2019; Characterisation	beta	
SRLGVGQETQ	Hardy; 2019; Characterisation	beta	
SFGLRDTGEL	Hardy; 2019; Characterisation	beta	
TLLAGGGGDTQ	Hardy; 2019; Characterisation	beta	
SLGGDSYEQ	Hardy; 2019; Characterisation	beta	
SFGTTNEKL	Hardy; 2019; Characterisation	beta	
SKGQGALLSSYEQ	Hardy; 2019; Characterisation	beta	
SLIPYNEQ	Hardy; 2019; Characterisation	beta	
SRSGGAWADTQ	Hardy; 2019; Characterisation	beta	
SIAWTGHVDGY	Hardy; 2019; Characterisation	beta	
TTGGSEKL	Hardy; 2019; Characterisation	beta	
GPGGDSYEQ	Hardy; 2019; Characterisation	beta	
ILGGDSYEQ	Hardy; 2019; Characterisation	beta	

GGAQKGYGY	Hardy; 2019; Characterisation	beta	
AGTGIGY	Hardy; 2019; Characterisation	beta	
GSQGDTEA	Hardy; 2019; Characterisation	beta	
REGIDTEA	Hardy; 2019; Characterisation	beta	
EQGWDSAEA	Hardy; 2019; Characterisation	beta	
SSNEKL	Hardy; 2019; Characterisation	beta	
RGWDTEA	Hardy; 2019; Characterisation	beta	
GQADTEA	Hardy; 2019; Characterisation	beta	
GEALGETQ	Hardy; 2019; Characterisation	beta	
ETSRGHEQ	Hardy; 2019; Characterisation	beta	
SEVAGGEQ	Hardy; 2019; Characterisation	beta	
SQEAPGQGYGQPQ	Hardy; 2019; Characterisation	beta	
SRTGIWDEQ	Hardy; 2019; Characterisation	beta	
SQDLTDSPL	Hardy; 2019; Characterisation	beta	
SQDLTDSPL	Hardy; 2019; Characterisation	beta	
SFGPLGSEA	Hardy; 2019; Characterisation	beta	
RGSDYEQ	Hardy; 2019; Characterisation	beta	
SVGGGAGDTQ	Hardy; 2019; Characterisation	beta	
SVGGPYNEQ	Hardy; 2019; Characterisation	beta	
SPWTSGYNQDTQ	Hardy; 2019; Characterisation	beta	
SLLGQAGY	Hardy; 2019; Characterisation	beta	

SLTGARTDTQ	Hardy; 2019; Characterisation	beta	
SAGISEEAG	Hardy; 2019; Characterisation	beta	
SIAWVETQ	Hardy; 2019; Characterisation	beta	
GGTAGSNYGY	Hardy; 2019; Characterisation	beta	
FKGGDTEA	Hardy; 2019; Characterisation	beta	
RDLRRTEA	Hardy; 2019; Characterisation	beta	
GRLTDTQ	Hardy; 2019; Characterisation	beta	
RRTFGQETQ	Hardy; 2019; Characterisation	beta	
AVGGDSYEQ	Hardy; 2019; Characterisation	beta	
SQGVEA	Hardy; 2019; Characterisation	beta	
RAGVDTEA	Hardy; 2019; Characterisation	beta	
SALQGASYEQ	Hardy; 2019; Characterisation	beta	
NSGWDGEL	Hardy; 2019; Characterisation	beta	
GYGLGETQ	Hardy; 2019; Characterisation	beta	
GGGYDQPQ	Hardy; 2019; Characterisation	beta	
SAGGWDTGEL	Hardy; 2019; Characterisation	beta	
SGQGGRA	Hardy; 2019; Characterisation	beta	
SSLQGRDGY	Hardy; 2019; Characterisation	beta	
SLFLAGNRSYEQ	Hardy; 2019; Characterisation	beta	
RNRGQASYEQ	Hardy; 2019; Characterisation	beta	
SVGLQVSAETQ	Hardy; 2019; Characterisation	beta	

SSWGQGESPL	Hardy; 2019; Characterisation	beta	
SLRGGQGGETQ	Hardy; 2019; Characterisation	beta	
SSFRNTEA	Hardy; 2019; Characterisation	beta	
SLAYSGYGY	Hardy; 2019; Characterisation	beta	
SHGQDTEA	Hardy; 2019; Characterisation	beta	
SLRGGQGGEKL	Hardy; 2019; Characterisation	beta	
SPGQGAYGY	Hardy; 2019; Characterisation	beta	
AREGTDTQ	Hardy; 2019; Characterisation	beta	
SSRGTDTQ	Hardy; 2019; Characterisation	beta	
SQGQDTEA	Hardy; 2019; Characterisation	beta	
SLLAGGDTQ	Hardy; 2019; Characterisation	alpha	
SERVGQGFGY	Hardy; 2019; Characterisation	alpha	
SELSSVWDEKL	Hardy; 2019; Characterisation	alpha	
SPVGAYEQ	Hardy; 2019; Characterisation	alpha	
TRQSPADTQ	Hardy; 2019; Characterisation	alpha	
REESGYSTL	Hardy; 2019; Characterisation	alpha	
RAVISGGYNKL	Hardy; 2019; Characterisation	alpha	
RAVLSGGYNKL	Hardy; 2019; Characterisation	alpha	
SRVQAGTAL	Hardy; 2019; Characterisation	alpha	
RSISGNTPL	Hardy; 2019; Characterisation	alpha	
GDDTGFQKL	Hardy; 2019; Characterisation	alpha	

GDSRGGKL	Hardy; 2019; Characterisation	alpha	
GDVDYNQGGKL	Hardy; 2019; Characterisation	alpha	
GDGAAGNKL	Hardy; 2019; Characterisation	alpha	
SSQLGGKL	Hardy; 2019; Characterisation	alpha	
AVGVEDSGNTPL	Hardy; 2019; Characterisation	alpha	
SVYSSASKI	Hardy; 2019; Characterisation	alpha	
APNQAGTAL	Hardy; 2019; Characterisation	alpha	
REDTGRRAL	Hardy; 2019; Characterisation	alpha	
TGGFKT	Hardy; 2019; Characterisation	alpha	
SDNWYGGATNKL	Hardy; 2019; Characterisation	alpha	
GGYGGSQGNL	Hardy; 2019; Characterisation	alpha	
SIGNYQL	Hardy; 2019; Characterisation	alpha	
DANYGQNF	Hardy; 2019; Characterisation	alpha	
SGAGANSKL	Hardy; 2019; Characterisation	alpha	
SRSYNTDKL	Hardy; 2019; Characterisation	alpha	
GPTGANNL	Hardy; 2019; Characterisation	alpha	
TTDSWGKL	Hardy; 2019; Characterisation	alpha	
SGGTGGFKT	Hardy; 2019; Characterisation	alpha	
NVVYEKL	Hardy; 2019; Characterisation	alpha	
SLAYTSGTYKY	Hardy; 2019; Characterisation	alpha	
LNYGGSQGNL	Hardy; 2019; Characterisation	alpha	

EKDSGYSTL	Hardy; 2019; Characterisation	alpha	
PHNAGNML	Hardy; 2019; Characterisation	alpha	
SGTYKY	Hardy; 2019; Characterisation	alpha	
RSGDYTGANSKL	Hardy; 2019; Characterisation	alpha	
ESGSARQL	Hardy; 2019; Characterisation	alpha	
RDLYNFNKF	Hardy; 2019; Characterisation	alpha	
RTDYLTGTASKL	Hardy; 2019; Characterisation	alpha	
GRPYTDKL	Hardy; 2019; Characterisation	alpha	
SMWGTSYGKL	Hardy; 2019; Characterisation	alpha	
SDYNFNKF	Hardy; 2019; Characterisation	alpha	
SEVPGGGNKL	Hardy; 2019; Characterisation	alpha	
RGSAGGTSYGKL	Hardy; 2019; Characterisation	alpha	
SLSGGYNKL	Hardy; 2019; Characterisation	alpha	
ILPXSWGKL	Hardy; 2019; Characterisation	alpha	
SEGGTYGNKL	Hardy; 2019; Characterisation	alpha	
QGTDSWGKL	Hardy; 2019; Characterisation	alpha	
SSAKTSYDKV	Hardy; 2019; Characterisation	alpha	
NRGSNYKL	Hardy; 2019; Characterisation	alpha	
SWGKL	Hardy; 2019; Characterisation	alpha	
QLGGQGAQKL	Hardy; 2019; Characterisation	alpha	
QSTGKL	Hardy; 2019; Characterisation	alpha	

VTSGSRL	Hardy; 2019; Characterisation	alpha	
MEENAGNML	Hardy; 2019; Characterisation	alpha	
SAKEYGNKL	Hardy; 2019; Characterisation	alpha	
RETSGSRL	Hardy; 2019; Characterisation	alpha	
SARWRAL	Hardy; 2019; Characterisation	alpha	
IAQPGTYNQGGKL	Hardy; 2019; Characterisation	alpha	
TGGGNKL	Hardy; 2019; Characterisation	alpha	
HPADSYDKV	Hardy; 2019; Characterisation	alpha	
MNIGYAL	Hardy; 2019; Characterisation	alpha	
GDSSYKL	Hardy; 2019; Characterisation	alpha	
EAGSYQL	Hardy; 2019; Characterisation	alpha	
SEGSTSGTYKY	Hardy; 2019; Characterisation	alpha	
SEGGNNFNKF	Hardy; 2019; Characterisation	alpha	
SEASGGKL	Hardy; 2019; Characterisation	alpha	
VGPSGSARQL	Hardy; 2019; Characterisation	alpha	
IGGGATNKL	Hardy; 2019; Characterisation	alpha	
FTSGTYKY	Hardy; 2019; Characterisation	alpha	
SAGGSARQL	Hardy; 2019; Characterisation	alpha	
SEAAGNKL	Hardy; 2019; Characterisation	alpha	
SAGSGGYNKL	Hardy; 2019; Characterisation	alpha	
KNTGFQKL	Hardy; 2019; Characterisation	alpha	

SDPTGANSKL	Hardy; 2019; Characterisation	alpha	
SDPTGTASKL	Hardy; 2019; Characterisation	alpha	
SGLRGSSTLGRL	Hardy; 2019; Characterisation	alpha	
GPSTLAGNKL	Hardy; 2019; Characterisation	alpha	
SSLGGGGNKL	Hardy; 2019; Characterisation	alpha	
RERGNNDM	Hardy; 2019; Characterisation	alpha	
QVGSGNTGKL	Hardy; 2019; Characterisation	alpha	
GDDTGFQKL	Hardy; 2019; Characterisation	alpha	
GDETGGYNKL	Hardy; 2019; Characterisation	alpha	
GEGDSNYQL	Hardy; 2019; Characterisation	alpha	
GPDNQGGKL	Hardy; 2019; Characterisation	alpha	
GDGGGYNKL	Hardy; 2019; Characterisation	alpha	
TSGTYKY	Hardy; 2019; Characterisation	alpha	
RGVFSGGYNKL	Hardy; 2019; Characterisation	alpha	
RDPGGKL	Hardy; 2019; Characterisation	alpha	
RGVFSGGYNKL	Hardy; 2019; Characterisation	alpha	
LFDSWGKL	Hardy; 2019; Characterisation	alpha	
GDDTGFQKL	Hardy; 2019; Characterisation	alpha	
GDTGFTGGGNKL	Hardy; 2019; Characterisation	alpha	
VGYSSASKI	Hardy; 2019; Characterisation	alpha	
RPSGYNKL	Hardy; 2019; Characterisation	alpha	

NRPTGFQKL	Hardy; 2019; Characterisation	alpha	
SGNDYKL	Hardy; 2019; Characterisation	alpha	
TGGTSYGKL	Hardy; 2019; Characterisation	alpha	
SEGNFNKF	Hardy; 2019; Characterisation	alpha	
SEGYNFNKF	Hardy; 2019; Characterisation	alpha	
SEATSGTYKY	Hardy; 2019; Characterisation	alpha	
TLGNFGNEKL	Hardy; 2019; Characterisation	alpha	
NSGFQKL	Hardy; 2019; Characterisation	alpha	
SEGLTGGGNKL	Hardy; 2019; Characterisation	alpha	
SEAAGNKL	Hardy; 2019; Characterisation	alpha	
SKGSSASKI	Hardy; 2019; Characterisation	alpha	
TTDSWGKL	Hardy; 2019; Characterisation	alpha	
SEAGANSKL	Hardy; 2019; Characterisation	alpha	
PNFGNEKL	Hardy; 2019; Characterisation	alpha	
QSFAGGTSYGKL	Hardy; 2019; Characterisation	alpha	
YTGTASKL	Hardy; 2019; Characterisation	alpha	
EGTTDSWGKL	Hardy; 2019; Characterisation	alpha	
NNNARL	Hardy; 2019; Characterisation	alpha	
GSGFQKL	Hardy; 2019; Characterisation	alpha	
APTEYGNKL	Hardy; 2019; Characterisation	alpha	
SGLAGGGADGL	Hardy; 2019; Characterisation	alpha	

ATYNFNKF	Hardy; 2019; Characterisation	alpha	
RINARL	Hardy; 2019; Characterisation	alpha	
QGATNKL	Hardy; 2019; Characterisation	alpha	
REAYNQGGKL	Hardy; 2019; Characterisation	alpha	
MGEYGGATNKL	Hardy; 2019; Characterisation	alpha	
NEWEYGNKL	Hardy; 2019; Characterisation	alpha	
REGGFGQF	Hardy; 2019; Characterisation	alpha	
DADSWGKL	Hardy; 2019; Characterisation	alpha	
TGGGNKL	Hardy; 2019; Characterisation	alpha	
MYSSYKL	Hardy; 2019; Characterisation	alpha	
RVAAVTGRRAL	Hardy; 2019; Characterisation	alpha	
RDPMNYGGSQGNL	Hardy; 2019; Characterisation	alpha	
FTGTASKL	Hardy; 2019; Characterisation	alpha	
ALSDHYSSGSARQLT	Gunnarsen; 2017; TCRalpha	alpha	ASSTAVLAGGPQY
IVTNNNDMR	Gunnarsen; 2017; TCRalpha	alpha	ASSIRSTDTQY
AESPGPGKLI	Gunnarsen; 2017; TCRalpha	alpha	ASSLRSADTQY
AVDPP	Gunnarsen; 2017; TCRalpha	alpha	ASSFRSTDTQY
AVTRNSGGYQKVT	Gunnarsen; 2017; TCRalpha	alpha	ASSIRSTDTQY
APDPSTSGTYKYI	Gunnarsen; 2017; TCRalpha	alpha	ASSLRFTDTQY
AEGITGANSKLT	Gunnarsen; 2017; TCRalpha	alpha	ASSIRATDTQY
AESGYSTLT	Gunnarsen; 2017; TCRalpha	alpha	ASSVRSTDTQY
AMKEYGNKLV	Gunnarsen; 2017; TCRalpha	alpha	ASSLRSTDTQY
ASSTAVLAGGPQY	Gunnarsen; 2017; TCRalpha	beta	ALSDHYSSGSARQLT
ASSIRSTDTQY	Gunnarsen; 2017; TCRalpha	beta	IVTNNNDMR
ASSLRSADTQY	Gunnarsen; 2017; TCRalpha	beta	AESPGPGKLI
ASSFRSTDTQY	Gunnarsen; 2017; TCRalpha	beta	AVDPP
ASSIRSTDTQY	Gunnarsen; 2017; TCRalpha	beta	AVTRNSGGYQKVT
ASSLRFTDTQY	Gunnarsen; 2017; TCRalpha	beta	APDPSTSGTYKYI

ASSIRATDTQY	Gunnarsen; 2017; TCRalpha	beta	AEGITGANSKLT
ASSVRSTDTQY	Gunnarsen; 2017; TCRalpha	beta	AESGYSTLT
ASSLRSTDTQY	Gunnarsen; 2017; TCRalpha	beta	AMKEYGNKLV
CASSQVTLPTETQYF	cook; 2020; circulating	beta	
CASGQVTLPTETQYF	cook; 2020; circulating	beta	
CASSSLNTQYF	cook; 2020; circulating	beta	
CTSSQVTLPTETQYF	cook; 2020; circulating	beta	
CASSQVTLPTGTQYF	cook; 2020; circulating	beta	
CASGQVILPTETQYF	cook; 2020; circulating	beta	
CASSFGVEDEQYF	cook; 2020; circulating	beta	
CASSSLNTQYF	cook; 2020; circulating	beta	
CASSIRSTDTQYF	cook; 2020; circulating	beta	
CASSQVTLPTETQYF	cook; 2020; circulating	beta	
CASNFGVEDEQYF	cook; 2020; circulating	beta	
CASSLGVEDEQYF	cook; 2020; circulating	beta	
CTSSSLNTQYF	cook; 2020; circulating	beta	
CASSQVTLPTETQYF	cook; 2020; circulating	beta	
CASSLGGSTDTQYF	cook; 2020; circulating	beta	
CSASSSGGASYNEQFF	cook; 2020; circulating	beta	
CASSRTGQGGETQYF	cook; 2020; circulating	beta	
CASSFGVEDEQYF	cook; 2020; circulating	beta	
CASSLSFDSPLHF	cook; 2020; circulating	beta	
CASSRSYGYTF	cook; 2020; circulating	beta	
CASSLSGADNSPLHF	cook; 2020; circulating	beta	
CASSVQGITNEKLFF	cook; 2020; circulating	beta	
CASSQVTLPTETRYF	cook; 2020; circulating	beta	
CASSQVTLPTETQHF	cook; 2020; circulating	beta	
CASSWGQGYQPQHF	cook; 2020; circulating	beta	
CPSFRGDIQYF	cook; 2020; circulating	beta	
CASSPLSFGGGQETQYF	cook; 2020; circulating	beta	
CAMTLNTDKLIF	Ting; 2020; molecular	alpha	CASSLQGGDYGYTF
CAFMKEDGGATNKLIF	Ting; 2020; molecular	alpha	CSASPGNTGVAQYF
CIVYLSNFGNEKLTF	Ting; 2020; molecular	alpha	CASSLIGGGELFF
CAASAHYGGSQGNLIF	Ting; 2020; molecular	alpha	CASGPGTATEAFF
CAYRSQETSGSRLTF	Ting; 2020; molecular	alpha	CSASWLGFGETQYF
CAYTPLETSGSRLTF	Ting; 2020; molecular	alpha	CSARAPITGRFYEQYF
CAVRPGGSQGNLIF	Ting; 2020; molecular	alpha	CASSLTTSEETQYF
CAASADLTNRDDKIIF	Ting; 2020; molecular	alpha	CASSLTTSEETQYF
CAFMKGSYGGSQGNLIF	Ting; 2020; molecular	alpha	CASSRTANYEQYF
FLLGITSGTYKYIF	Ting; 2020; molecular	alpha	CASSRTANYEQYF
CIVRVAEGAQKLVF	Ting; 2020; molecular	alpha	CSATKETQYF

CIVRVAEGAQKLVF	Ting; 2020; molecular	alpha	CSATKETQYF
CAVHTGARLMF	Ting; 2020; molecular	alpha	CASSHGASTDTQYF
CIVRVAEGAQKLVF	Ting; 2020; molecular	alpha	CSATKETQYF
CALVRDYGQNFVF	Ting; 2020; molecular	alpha	CASSTSGGGARSYEQYF
CAWTGGGGFKTIF	Ting; 2020; molecular	alpha	CASRKPGGDYEQYF
CAVIGTSGSRLTF	Ting; 2020; molecular	alpha	CASSSGAATDTQYF
CIVRPAQGAQKLVF	Ting; 2020; molecular	alpha	CSVSSGGEGGNGYTF
CASPQGGSEKLVF	Ting; 2020; molecular	alpha	CASSSGGWGGGTEAFF
CAGRDDFNKFYF	Ting; 2020; molecular	alpha	CASSSGGWGGGTEAFF
CALGLNNNAGNMLTF	Ting; 2020; molecular	alpha	CAISPRQAETQYF
CAASGYSTLTF	Ting; 2020; molecular	alpha	CASTHGAGSDTQYF
CAVSGNTGFQKLVF	Ting; 2020; molecular	alpha	CASTSRGGNSNQPQHF
CALGATVMDSSYKLI	Ting; 2020; molecular	alpha	CASTSRGGNSNQPQHF
CASSLQGGDYGYTF	Ting; 2020; molecular	beta	CAMTLNTDKLIF
CSASPGNTGVAQYF	Ting; 2020; molecular	beta	CAFMKEDGGATNKLIF
CASSLIGGGELFF	Ting; 2020; molecular	beta	CIVYLSNFGNEKLTF
CASGPGTATEAFF	Ting; 2020; molecular	beta	CAASAHYGGSQGNLIF
CSASWLGFGETQYF	Ting; 2020; molecular	beta	CAYRSQETSGSRLTF
CSARAPITGRFYEQYF	Ting; 2020; molecular	beta	CAYTPLETSGSRLTF
CASSLTTSEETQYF	Ting; 2020; molecular	beta	CAVRPGGSQGNLIF
CASSLTTSEETQYF	Ting; 2020; molecular	beta	CAASADLTNRDDKIIF
CASSRTANYEQYF	Ting; 2020; molecular	beta	CAFMKGSYGGSQGNLIF
CASSRTANYEQYF	Ting; 2020; molecular	beta	FLLGITSGTYKYIF
CSATKETQYF	Ting; 2020; molecular	beta	CIVRVAEGAQKLVF
CSATKETQYF	Ting; 2020; molecular	beta	CIVRVAEGAQKLVF
CASSHGASTDTQYF	Ting; 2020; molecular	beta	CAVHTGARLMF
CSATKETQYF	Ting; 2020; molecular	beta	CIVRVAEGAQKLVF
CASSTSGGGARSYEQYF	Ting; 2020; molecular	beta	CALVRDYGQNFVF
CASRKPGGDYEQYF	Ting; 2020; molecular	beta	CAWTGGGGFKTIF
CASSSGAATDTQYF	Ting; 2020; molecular	beta	CAVIGTSGSRLTF
CSVSSGGEGGNGYTF	Ting; 2020; molecular	beta	CIVRPAQGAQKLVF
CASSSGGWGGGTEAFF	Ting; 2020; molecular	beta	CASPQGGSEKLVF
CASSSGGWGGGTEAFF	Ting; 2020; molecular	beta	CAGRDDFNKFYF
CAISPRQAETQYF	Ting; 2020; molecular	beta	CALGLNNNAGNMLTF
CASTHGAGSDTQYF	Ting; 2020; molecular	beta	CAASGYSTLTF
CASTSRGGNSNQPQHF	Ting; 2020; molecular	beta	CAVSGNTGFQKLVF
CASTSRGGNSNQPQHF	Ting; 2020; molecular	beta	CALGATVMDSSYKLI
IVTNNNDMR	qiao; 2013; biased	alpha	IRSTDT
IAWANDMR	qiao; 2013; biased	alpha	LRSTDT
IGFNDKLS	qiao; 2013; biased	alpha	LRSTDT
TDSGGGADGLT	qiao; 2013; biased	alpha	LRFTDT

IVFGGYQKVT	qiao; 2013; biased	alpha	LRATDT
IVTGGADGLT	qiao; 2013; biased	alpha	IRTTDT
IAPYGGATNKLI	qiao; 2013; biased	alpha	IRWTDT
IDFGGSQGNLI	qiao; 2013; biased	alpha	IRATDT
IVTNGQNFV	qiao; 2013; biased	alpha	LRTSDT
ISLNGAQKLV	qiao; 2013; biased	alpha	RRSTDT
IANYGGSQGNLI	qiao; 2013; biased	alpha	MRFTDT
IVNGGANNLF	qiao; 2013; biased	alpha	IRHTDT
IVYGGSQGNLI	qiao; 2013; biased	alpha	FRNTDT
IVFNDYKLS	qiao; 2013; biased	alpha	FRSTDT
IAYGKLT	qiao; 2013; biased	alpha	VRFTDT
IALNARLM	qiao; 2013; biased	alpha	LRATDT
IVYNDYKLS	qiao; 2013; biased	alpha	LRTTDT
IVHQGKLI	qiao; 2013; biased	alpha	IRFTDT
IVMGGADGLT	qiao; 2013; biased	alpha	FRSTDT
IVYNDYKLS	qiao; 2013; biased	alpha	LRTTDT
IAPFGNDMR	qiao; 2013; biased	alpha	IRETDT
IAFNDYKLS	qiao; 2013; biased	alpha	LRSTDT
IAYNDYKLS	qiao; 2013; biased	alpha	FRSTDT
IGDYGGSQGNLI	qiao; 2013; biased	alpha	LRFTDT
IVYNNARLM	qiao; 2013; biased	alpha	VRHTDT
IGNYGGSQGNLI	qiao; 2013; biased	alpha	LRYTDT
IVFGGSQGNLI	qiao; 2013; biased	alpha	IRATDT
IVLSQNFV	qiao; 2013; biased	alpha	LRYTDT
IAPDNAGNMLTF	qiao; 2013; biased	alpha	IRFTDT
IAWGGYGQNFV	qiao; 2013; biased	alpha	IRATDT
IRSTDT	qiao; 2013; biased	beta	IVTNNNDMR
LRSTDT	qiao; 2013; biased	beta	IAWANDMR
LRSTDT	qiao; 2013; biased	beta	IGFNDKLS
LRFTDT	qiao; 2013; biased	beta	TDSGGGADGLT
LRATDT	qiao; 2013; biased	beta	IVFGGYQKVT
IRTTDT	qiao; 2013; biased	beta	IVTGGADGLT
IRWTDT	qiao; 2013; biased	beta	IAPYGGATNKLI
IRATDT	qiao; 2013; biased	beta	IDFGGSQGNLI
LRTSDT	qiao; 2013; biased	beta	IVTNGQNFV
RRSTDT	qiao; 2013; biased	beta	ISLNGAQKLV
MRFTDT	qiao; 2013; biased	beta	IANYGGSQGNLI
IRHTDT	qiao; 2013; biased	beta	IVNGGANNLF
FRNTDT	qiao; 2013; biased	beta	IVYGGSQGNLI
FRSTDT	qiao; 2013; biased	beta	IVFNDYKLS
VRFTDT	qiao; 2013; biased	beta	IAYGKLT

LRATDT	qiao; 2013; biased	beta	IALNARLM
LRTTDT	qiao; 2013; biased	beta	IVYNDYKLS
IRFTDT	qiao; 2013; biased	beta	IVHQGKLI
FRSTDT	qiao; 2013; biased	beta	IVMGGADGLT
LRTTDT	qiao; 2013; biased	beta	IVYNDYKLS
IRETDT	qiao; 2013; biased	beta	IAPFGNDMR
LRSTDT	qiao; 2013; biased	beta	IAFNDYKLS
FRSTDT	qiao; 2013; biased	beta	IAYNDYKLS
LRFTDT	qiao; 2013; biased	beta	IGDYGGSQGNLI
VRHTDT	qiao; 2013; biased	beta	IVYNNARLM
LRYTDT	qiao; 2013; biased	beta	IGNYGGSQGNLI
IRATDT	qiao; 2013; biased	beta	IVFGGSQGNLI
LRYTDT	qiao; 2013; biased	beta	IVLSQNFV
IRFTDT	qiao; 2013; biased	beta	IAPDNAGNMLTF
IRATDT	qiao; 2013; biased	beta	IAWGGYGQNFV
ASSIRSTDTQY	Qiao; 2011; posttranslational	beta	
ASSLRYTDTQY	Qiao; 2011; posttranslational	beta	
ASSLRSTDTQY	Qiao; 2011; posttranslational	beta	
ASSFRSTDTQY	Qiao; 2011; posttranslational	beta	
ASSLRFTDTQY	Qiao; 2011; posttranslational	beta	
ASSIRATDTQY	Qiao; 2011; posttranslational	beta	
CAASEGDSGTYKYIF	Dahal-Koirala; 2019; discrim	alpha	CASSINALVGEQFF
CAVKFASGTYKYIF	Dahal-Koirala; 2019; discrim	alpha	CASSPAFSTDTQYF
CAMREGWQAGNTLIF	Dahal-Koirala; 2019; discrim	alpha	CASSLDGLTNTEAFF
CAVVDASSKLIF	Dahal-Koirala; 2019; discrim	alpha	CSATLGGDYGYTF
CLVGGYNNNDMRF	Dahal-Koirala; 2019; discrim	alpha	CSAQLAGGGGDTQYF
CAESRYSGYSTLTF	Dahal-Koirala; 2019; discrim	alpha	CSAFPGGDTEAFF
CALSEYGNKLVF	Dahal-Koirala; 2019; discrim	alpha	CASSYVGGDTDTQYF
CASSINALVGEQFF	Dahal-Koirala; 2019; discrim	beta	CAASEGDSGTYKYIF
CASSPAFSTDTQYF	Dahal-Koirala; 2019; discrim	beta	CAVKFASGTYKYIF
CASSLDGLTNTEAFF	Dahal-Koirala; 2019; discrim	beta	CAMREGWQAGNTLIF
CSATLGGDYGYTF	Dahal-Koirala; 2019; discrim	beta	CAVVDASSKLIF
CSAQLAGGGGDTQYF	Dahal-Koirala; 2019; discrim	beta	CLVGGYNNNDMRF
CSAFPGGDTEAFF	Dahal-Koirala; 2019; discrim	beta	CAESRYSGYSTLTF
CASSYVGGDTDTQYF	Dahal-Koirala; 2019; discrim	beta	CALSEYGNKLVF
SLRTSGSHEQ	Hardy; 2015; consistency	beta	
STRTYGGNIQ	Hardy; 2015; consistency	beta	
SLRWTDTQ	Hardy; 2015; consistency	beta	
SLRSSGSHEQ	Hardy; 2015; consistency	beta	
SIRSTDTQ	Hardy; 2015; consistency	beta	
SIRLADEGEQ	Hardy; 2015; consistency	beta	

SLRFTDTQ	Hardy; 2015; consistency	beta	
SLRAAGENIQ	Hardy; 2015; consistency	beta	
SLRTSGGHEQ	Hardy; 2015; consistency	beta	
SLIGAETQ	Hardy; 2015; consistency	beta	
SRRSTDTQ	Hardy; 2015; consistency	beta	
SVRALGADTQ	Hardy; 2015; consistency	beta	
IDPRGPGYGY	Hardy; 2015; consistency	beta	
SFRVLAQETQ	Hardy; 2015; consistency	beta	
SIRATDTQ	Hardy; 2015; consistency	beta	
SLRDYETQ	Hardy; 2015; consistency	beta	
SPRTSGGLFTYEQ	Hardy; 2015; consistency	beta	
SLRSTDTQ	Hardy; 2015; consistency	beta	
SLAPLTSTDTQ	Hardy; 2015; consistency	beta	
SLVARDHEQ	Hardy; 2015; consistency	beta	
SFRFTDTQ	Hardy; 2015; consistency	beta	
SVRTSGSHEQ	Hardy; 2015; consistency	beta	
STRWGDEGEQ	Hardy; 2015; consistency	beta	
SLRAGGGTQ	Hardy; 2015; consistency	beta	
SIRSTDTQ	Hardy; 2015; consistency	beta	
SLRYTDTQ	Hardy; 2015; consistency	beta	
SLRTGGENEQ	Hardy; 2015; consistency	beta	
SIRAGDGNTQ	Hardy; 2015; consistency	beta	
SFRTTDTQ	Hardy; 2015; consistency	beta	
SLRSTDTQ	Hardy; 2015; consistency	beta	
SLAASEPGY	Hardy; 2015; consistency	beta	
SLKWDYEQ	Hardy; 2015; consistency	beta	
CAYRSGYMEYGNKLV	Peterson; 2014; T-cell	alpha	CSVSGGPSTDTQYF
CLVGDNDYKLSF	Peterson; 2014; T-cell	alpha	CSVGQTDSPLHF
CAVELGDSWGKFQF	Peterson; 2014; T-cell	alpha	CSAGQGGTGELFF
CAAPGGATNKLIF	Peterson; 2014; T-cell	alpha	CSVAALAGFQETQYF
CAVGWEDGTASKLTF	Peterson; 2014; T-cell	alpha	CSASRWRSTDTQYF
CLVGDGGSFSGGYNKLIF	Peterson; 2014; T-cell	alpha	CSAGVGGQETQYF
CAVQADGNTGKLIF	Peterson; 2014; T-cell	alpha	CSAYRTWDQETQYF
CAFIGGSNYKLTF	Peterson; 2014; T-cell	alpha	CSAREPDNTGELFF
ND (not determined)	Peterson; 2014; T-cell	alpha	CSARGYGLANPYEQY
CAGPYNTDKLIF	Peterson; 2014; T-cell	alpha	CASSLASAGGTDTQYF
CAASANYGGATNKLIF	Peterson; 2014; T-cell	alpha	CASSLNWDTEAFF
CAVTLGGGSEKLVF	Peterson; 2014; T-cell	alpha	CASSFSPAGSEAFF
CAVGAGSNYQLIW	Peterson; 2014; T-cell	alpha	CASSLEGQGASEQFF
CLVAGAGGYNKLIF	Peterson; 2014; T-cell	alpha	CASSQGLAGVQETQYF
CAGPSGATNKLIF	Peterson; 2014; T-cell	alpha	CASSPTALGTDTQYF

CIVLGGADGLTF	Peterson; 2014; T-cell	alpha	CASSFRFTDTQYF
CIVWGGATNKLIF	Peterson; 2014; T-cell	alpha	CASSVRSTDTQYF
CIAFQGAQKLVF	Peterson; 2014; T-cell	alpha	CASSFRALAADTQYF
CLVGEAAGNKLTF	Peterson; 2014; T-cell	alpha	CASSIRTSGDHEQYF
CAVGRGGSQGNLIF	Peterson; 2014; T-cell	alpha	CASSQYQSLVRGNNEQFF
CAMREGRGAGSYQLTF	Peterson; 2014; T-cell	alpha	CASSFPQVTDTQYF
CAMSVLSGTYKYIF	Peterson; 2014; T-cell	alpha	CASSHVDRGGETQYF
CSVSGGPSTDTQYF	Peterson; 2014; T-cell	beta	CAYRSGYMEYGNKLV
CSVGQTDSPLHF	Peterson; 2014; T-cell	beta	CLVGDNDYKLSF
CSAGQGGTGELFF	Peterson; 2014; T-cell	beta	CAVELGDSWGKFQF
CSVAALAGFQETQYF	Peterson; 2014; T-cell	beta	CAAPGGATNKLIF
CSASRWRSTDTQYF	Peterson; 2014; T-cell	beta	CAVGWEDGTASKLTF
CSAGVGGQETQYF	Peterson; 2014; T-cell	beta	CLVGDGGSFSGGYNKLIF
CSAYRTWDQETQYF	Peterson; 2014; T-cell	beta	CAVQADGNTGKLIF
CSAREPDNTGELFF	Peterson; 2014; T-cell	beta	CAFIGGSNYKLTF
CSARGYGLANPYEQY	Peterson; 2014; T-cell	beta	ND (not determined)
CASSLASAGGTDTQYF	Peterson; 2014; T-cell	beta	CAGPYNTDKLIF
CASSLNWDTEAFF	Peterson; 2014; T-cell	beta	CAASANYGGATNKLIF
CASSFSPAGSEAFF	Peterson; 2014; T-cell	beta	CAVTLGGGSEKLVF
CASSLEGQGASEQFF	Peterson; 2014; T-cell	beta	CAVGAGSNYQLIW
CASSQGLAGVQETQYF	Peterson; 2014; T-cell	beta	CLVAGAGGYNKLIF
CASSPTALGTDTQYF	Peterson; 2014; T-cell	beta	CAGPSGATNKLIF
CASSFRFTDTQYF	Peterson; 2014; T-cell	beta	CIVLGGADGLTF
CASSVRSTDTQYF	Peterson; 2014; T-cell	beta	CIVWGGATNKLIF
CASSFRALAADTQYF	Peterson; 2014; T-cell	beta	CIAFQGAQKLVF
CASSIRTSGDHEQYF	Peterson; 2014; T-cell	beta	CLVGEAAGNKLTF
CASSQYQSLVRGNNEQFF	Peterson; 2014; T-cell	beta	CAVGRGGSQGNLIF
CASSFPQVTDTQYF	Peterson; 2014; T-cell	beta	CAMREGRGAGSYQLTF
CASSHVDRGGETQYF	Peterson; 2014; T-cell	beta	CAMSVLSGTYKYIF
CASSPGTDTQYF	Han; 2013; dietary	beta	
CASSFGGELFF	Han; 2013; dietary	beta	
CASAGNYEKLFF	Han; 2013; dietary	beta	
CASSAGHPEQFF	Han; 2013; dietary	beta	
CASSLINTEAFF	Han; 2013; dietary	beta	
CASSNLRQGAAGNTIYF	Han; 2013; dietary	beta	
CASSQEEQGAFYEQFF	Han; 2013; dietary	beta	
CASTEGQAEAFF	Han; 2013; dietary	beta	
CATSGTSGYNEQFF	Han; 2013; dietary	beta	
CSVDGNYLTDTQYF	Han; 2013; dietary	beta	
CASRIQGEGSPLHF	Han; 2013; dietary	beta	
CASSPGTNTQYF	Han; 2013; dietary	beta	

CASSQDLGDYGYTF	Han; 2013; dietary	beta	
CSVEMNTEAFF	Han; 2013; dietary	beta	
CAISDPPLATEAFF	Han; 2013; dietary	beta	
CAISSGQVPEQFF	Han; 2013; dietary	beta	
CASEMDANTGELFF	Han; 2013; dietary	beta	
CASKLGGATEAFF	Han; 2013; dietary	beta	
CASNQGQGVETQYF	Han; 2013; dietary	beta	
CASSEGTYEQYF	Han; 2013; dietary	beta	
CASSFGTDTQYF	Han; 2013; dietary	beta	
CASSFGTSDQFF	Han; 2013; dietary	beta	
CASSFLGTYEQYF	Han; 2013; dietary	beta	
CASSFPNPTFEAFF	Han; 2013; dietary	beta	
CASSFRGQGNEQYF	Han; 2013; dietary	beta	
CASSFTGSSYEQYF	Han; 2013; dietary	beta	
CASSGGTVYGYTF	Han; 2013; dietary	beta	
CASSGSGGVTGELFF	Han; 2013; dietary	beta	
CASSLASVGSTEAFF	Han; 2013; dietary	beta	
CASSLGTGGYNEQFF	Han; 2013; dietary	beta	
CASSLLGLTGELFF	Han; 2013; dietary	beta	
CASSLNLGQNTEAFF	Han; 2013; dietary	beta	
CASSLPLGQGNQPQHF	Han; 2013; dietary	beta	
CASSLPPRGGGYEQYF	Han; 2013; dietary	beta	
CASSPEDPYTDTQYF	Han; 2013; dietary	beta	
CASSPEREVYNEQFF	Han; 2013; dietary	beta	
CASSPFSGDYYEQYF	Han; 2013; dietary	beta	
CASSPGTNIQYF	Han; 2013; dietary	beta	
CASSPGTVVYGYTF	Han; 2013; dietary	beta	
CASSPGTYEQYF	Han; 2013; dietary	beta	
CASSPGVYEQYF	Han; 2013; dietary	beta	
CASSPSNTGELFF	Han; 2013; dietary	beta	
CASSQAGALWDYGYTF	Han; 2013; dietary	beta	
CASSQGIRSEYEQYF	Han; 2013; dietary	beta	
CASSQLPVNSPLHF	Han; 2013; dietary	beta	
CASSSGLATDTQYF	Han; 2013; dietary	beta	
CASSTGHMEDTQYF	Han; 2013; dietary	beta	
CASSTGVSGANVLTF	Han; 2013; dietary	beta	
CASSVEGGMGEKLFF	Han; 2013; dietary	beta	
CASSVGAGVNSYEQYF	Han; 2013; dietary	beta	
CASSVRAGTGTYEQYF	Han; 2013; dietary	beta	
CASSVSTGSYEQYF	Han; 2013; dietary	beta	
CASSWDRATNEKLFF	Han; 2013; dietary	beta	

CASSYAPTGNYGYTF	Han; 2013; dietary	beta	
CASSYKRGPGELFF	Han; 2013; dietary	beta	
CASSYSMASGGAQETQYF	Han; 2013; dietary	beta	
CASSYTAGSNQPQHF	Han; 2013; dietary	beta	
CASSYWEEGGGAFF	Han; 2013; dietary	beta	
CASSYWGPMNTEAFF	Han; 2013; dietary	beta	
CAWSVKTLRRADTQYF	Han; 2013; dietary	beta	
CSAAGHFYEQYF	Han; 2013; dietary	beta	
CSAMTQEDYAFF	Han; 2013; dietary	beta	
CSARDFSLRTGELFF	Han; 2013; dietary	beta	
CSARDMFGGHVSGNTIYF	Han; 2013; dietary	beta	
CSVDGLAGITDTQYF	Han; 2013; dietary	beta	
CASSPGTDTQYF	Han; 2013; dietary	beta	
CSVEMNTEAFF	Han; 2013; dietary	beta	
CASSLLAGGAEQFF	Han; 2013; dietary	beta	
CASSNLRQGAAGNTIYF	Han; 2013; dietary	beta	
CASSQEEQGAFYEQFF	Han; 2013; dietary	beta	
CASSSSDRATDTQYF	Han; 2013; dietary	beta	
CAWSVKTLRRADTQYF	Han; 2013; dietary	beta	
CASSAGHPEQFF	Han; 2013; dietary	beta	
CASSLASVGSTEAFF	Han; 2013; dietary	beta	
CASSYRQAGYEQYF	Han; 2013; dietary	beta	
CASTEGQAEAFF	Han; 2013; dietary	beta	
CASSLPTAVTEAFF	Han; 2013; dietary	beta	
CASEMDANTGELFF	Han; 2013; dietary	beta	
CASRIQGEGSPLHF	Han; 2013; dietary	beta	
CASSDRGFPSYEQYF	Han; 2013; dietary	beta	
CASSFGGELFF	Han; 2013; dietary	beta	
CASSFGNQPQHF	Han; 2013; dietary	beta	
CASSFSLAGWSYNEQFF	Han; 2013; dietary	beta	
CASSFSNGENTDTQYF	Han; 2013; dietary	beta	
CASSHLGGGNTIYF	Han; 2013; dietary	beta	
CASSLAGDSYNEQFF	Han; 2013; dietary	beta	
CASSLGQGTPDTQYF	Han; 2013; dietary	beta	
CASSLINTEAFF	Han; 2013; dietary	beta	
CASSLSLAGDTGELFF	Han; 2013; dietary	beta	
CASSLTRQGGEGSPLHF	Han; 2013; dietary	beta	
CASSLVADSYNEQFF	Han; 2013; dietary	beta	
CASSLVGPGDTQYF	Han; 2013; dietary	beta	
CASSPFSGDYYEQYF	Han; 2013; dietary	beta	
CASSPGTALAFF	Han; 2013; dietary	beta	

CASSQDGGARQHF	Han; 2013; dietary	beta	
CASSQDSGGARNNEQFF	Han; 2013; dietary	beta	
CASSQDWALGWGYGYTF	Han; 2013; dietary	beta	
CASSQLTQNTEAFF	Han; 2013; dietary	beta	
CASSQPFVGSGNTIYF	Han; 2013; dietary	beta	
CASSRDWGETQYF	Han; 2013; dietary	beta	
CASSRGLAGESTDTQYF	Han; 2013; dietary	beta	
CASSRPASYEQYF	Han; 2013; dietary	beta	
CASSSGADLYGYTF	Han; 2013; dietary	beta	
CASSSGLSYNEQFF	Han; 2013; dietary	beta	
CASSSGTLETQYF	Han; 2013; dietary	beta	
CASSSSSGSTYEQYF	Han; 2013; dietary	beta	
CASSSWTGTNTEAFF	Han; 2013; dietary	beta	
CASSWLAGGPAGELFF	Han; 2013; dietary	beta	
CASSYATGTPSSYNEQFF	Han; 2013; dietary	beta	
CASSYGSDSYNEQFF	Han; 2013; dietary	beta	
CSARVSGSLYEQYF	Han; 2013; dietary	beta	
CSVDGNYLTDTQYF	Han; 2013; dietary	beta	
CSVEMSGGDYEQYF	Han; 2013; dietary	beta	
CASSYDVRSGNYEQYF	Han; 2013; dietary	beta	
CASSPSDPSDTQYF	Han; 2013; dietary	beta	
CASSPGTGSGDEQFF	Han; 2013; dietary	beta	
CASSVGGVQPQHF	Han; 2013; dietary	beta	
CASNLAGGSNEQFF	Han; 2013; dietary	beta	
CASSFGQVTYEQYF	Han; 2013; dietary	beta	
CASSLGGAETQYF	Han; 2013; dietary	beta	
CASSQDWGDYGYTF	Han; 2013; dietary	beta	
CASSSEQDRGSENTIYF	Han; 2013; dietary	beta	
CASSWSGYEQYF	Han; 2013; dietary	beta	
CASSWTGNEQFF	Han; 2013; dietary	beta	
CAIRATGLAGVDTGELFF	Han; 2013; dietary	beta	
CAISEQEYGTEAFF	Han; 2013; dietary	beta	
CAISFGTGEAPRGYTF	Han; 2013; dietary	beta	
CAISPDRVTFEVFF	Han; 2013; dietary	beta	
CALWGGAYEQYF	Han; 2013; dietary	beta	
CASCFTSLGTGELFF	Han; 2013; dietary	beta	
CASGSSEQFF	Han; 2013; dietary	beta	
CASGTSQAYEQYF	Han; 2013; dietary	beta	
CASIQGHEQYF	Han; 2013; dietary	beta	
CASNAGAGFGYEQYF	Han; 2013; dietary	beta	
CASRGTVRGGYEQYF	Han; 2013; dietary	beta	

CASRLGTAPAFF	Han; 2013; dietary	beta	
CASRRTAATNEKLFF	Han; 2013; dietary	beta	
CASSARNSNQPQHF	Han; 2013; dietary	beta	
CASSARTGAYGYTF	Han; 2013; dietary	beta	
CASSDEAGYGYTF	Han; 2013; dietary	beta	
CASSDLPSGAPQETQYF	Han; 2013; dietary	beta	
CASSETASSTDTQYF	Han; 2013; dietary	beta	
CASSFAETQYF	Han; 2013; dietary	beta	
CASSFDPRGEKLFF	Han; 2013; dietary	beta	
CASSFGTGSSETQYF	Han; 2013; dietary	beta	
CASSFGVYNEQFF	Han; 2013; dietary	beta	
CASSFPPSGDTDTQYF	Han; 2013; dietary	beta	
CASSFSGSDTGELFF	Han; 2013; dietary	beta	
CASSFTGTPTYEQYF	Han; 2013; dietary	beta	
CASSHGQGNQPQHF	Han; 2013; dietary	beta	
CASSHPTRSYNEQFF	Han; 2013; dietary	beta	
CASSHRRGIPPPPLYNEQFF	Han; 2013; dietary	beta	
CASSIAARAGELFF	Han; 2013; dietary	beta	
CASSIGVLNTEAFF	Han; 2013; dietary	beta	
CASSKLDSGYTF	Han; 2013; dietary	beta	
CASSKPPETQYF	Han; 2013; dietary	beta	
CASSLAGGIAKNIQYF	Han; 2013; dietary	beta	
CASSLAPWMDYGYTF	Han; 2013; dietary	beta	
CASSLFTGGTYEQYF	Han; 2013; dietary	beta	
CASSLGGLELGFEQFF	Han; 2013; dietary	beta	
CASSLGGSNQPQHF	Han; 2013; dietary	beta	
CASSLGLGTGELFF	Han; 2013; dietary	beta	
CASSLGRVEAFF	Han; 2013; dietary	beta	
CASSLGTSQETQYF	Han; 2013; dietary	beta	
CASSLLRLAGETTYNEQFF	Han; 2013; dietary	beta	
CASSLPGTGTSPLHF	Han; 2013; dietary	beta	
CASSLRGYTDTQYF	Han; 2013; dietary	beta	
CASSLRQGGYEQYF	Han; 2013; dietary	beta	
CASSLSADRDGGYTF	Han; 2013; dietary	beta	
CASSLSQGGHNEQFF	Han; 2013; dietary	beta	
CASSLVPPGGFSYEQYF	Han; 2013; dietary	beta	
CASSMGQGNSGETQYF	Han; 2013; dietary	beta	
CASSPGGAGYTF	Han; 2013; dietary	beta	
CASSPGGWSYEQYF	Han; 2013; dietary	beta	
CASSPGQGNNSPLHF	Han; 2013; dietary	beta	
CASSPISRDRNTGELFF	Han; 2013; dietary	beta	

CASSPLGSGTEAFF	Han; 2013; dietary	beta	
CASSPNPNTGELFF	Han; 2013; dietary	beta	
CASSPPDRGYDNEQFF	Han; 2013; dietary	beta	
CASSPPLTEAFF	Han; 2013; dietary	beta	
CASSPRLAGAKDTQYF	Han; 2013; dietary	beta	
CASSPRSAGGPYEQYF	Han; 2013; dietary	beta	
CASSPTSGRTTSYEQYF	Han; 2013; dietary	beta	
CASSQDGGTYNEQFF	Han; 2013; dietary	beta	
CASSQGDRDYEQFF	Han; 2013; dietary	beta	
CASSSGTAQSEKLFF	Han; 2013; dietary	beta	
CASSSLGTEVYEQYF	Han; 2013; dietary	beta	
CASSSPGDSYEQYF	Han; 2013; dietary	beta	
CASSSSGRAQTDTQYF	Han; 2013; dietary	beta	
CASSSTGGISWNTEAFF	Han; 2013; dietary	beta	
CASSSTGPPFNYGYTF	Han; 2013; dietary	beta	
CASSSTPGGLWYGYTF	Han; 2013; dietary	beta	
CASSSTYEQYF	Han; 2013; dietary	beta	
CASSTRTTHTYSNQPQHF	Han; 2013; dietary	beta	
CASSVKRLNTEAFF	Han; 2013; dietary	beta	
CASSYGQSFEQYF	Han; 2013; dietary	beta	
CASSYKQGIHEQYF	Han; 2013; dietary	beta	
CASSYSQGNYGYTF	Han; 2013; dietary	beta	
CASSYTPGGNTDTQYF	Han; 2013; dietary	beta	
CASSYWAGDYGYTF	Han; 2013; dietary	beta	
CASTAGFNQPQHF	Han; 2013; dietary	beta	
CASTDVTSGQETQYF	Han; 2013; dietary	beta	
CASTGIAGPTDTQYF	Han; 2013; dietary	beta	
CATIGPAGDTQYF	Han; 2013; dietary	beta	
CATPSGNTIYF	Han; 2013; dietary	beta	
CATSDLGLGVNEQFF	Han; 2013; dietary	beta	
CATSRAQGQPQHF	Han; 2013; dietary	beta	
CATSRDHSSGASQGNIQYF	Han; 2013; dietary	beta	
CATSRDNTGYTGELFF	Han; 2013; dietary	beta	
CATSRDQGSDTQYF	Han; 2013; dietary	beta	
CAWDSTGISYNEQFF	Han; 2013; dietary	beta	
CAWRATEGQETQYF	Han; 2013; dietary	beta	
CAWSDSGSSYEQYF	Han; 2013; dietary	beta	
CAWSVTGTRLYEQYF	Han; 2013; dietary	beta	
CSALDSYSNQPQHF	Han; 2013; dietary	beta	
CSARAAIGTMNTEAFF	Han; 2013; dietary	beta	
CSARDADGYESEKLFF	Han; 2013; dietary	beta	

CSARDRQGYSNQPQHF	Han; 2013; dietary	beta	
CSARPPNGRYNEQFF	Han; 2013; dietary	beta	
CSARVKGLAGIRSYEQYF	Han; 2013; dietary	beta	
CSDTVRRGPGGYTF	Han; 2013; dietary	beta	
CSVLGQGPSYEQYF	Han; 2013; dietary	beta	
CSVVEGGEQYF	Han; 2013; dietary	beta	
CAWSGVSADTQYF	Han; 2013; dietary	beta	
CASSLGARTGELFF	Han; 2013; dietary	beta	
CASSPRSRTDTQYF	Han; 2013; dietary	beta	
CASSVGGVQPQHF	Han; 2013; dietary	beta	
CSAREGDTQYF	Han; 2013; dietary	beta	
CASSSGELFF	Han; 2013; dietary	beta	
CASDRGETQYF	Han; 2013; dietary	beta	
CASGESNEQYF	Han; 2013; dietary	beta	
CASKPLQGYNEQFF	Han; 2013; dietary	beta	
CASNIRASNQPQHF	Han; 2013; dietary	beta	
CASNLAGGSNEQFF	Han; 2013; dietary	beta	
CASQRQSYEQYF	Han; 2013; dietary	beta	
CASRFGQGGNSNQPQHF	Han; 2013; dietary	beta	
CASRSRGTIYEQYF	Han; 2013; dietary	beta	
CASSDGLAYEQYF	Han; 2013; dietary	beta	
CASSDTGSINQPQHF	Han; 2013; dietary	beta	
CASSFASSSGNTIYF	Han; 2013; dietary	beta	
CASSFIVLSGSSYEQYF	Han; 2013; dietary	beta	
CASSFSGRTYEQYF	Han; 2013; dietary	beta	
CASSFSQVDEQFF	Han; 2013; dietary	beta	
CASSGQQGGSYGYTF	Han; 2013; dietary	beta	
CASSGSATGELFF	Han; 2013; dietary	beta	
CASSKLDSGYTF	Han; 2013; dietary	beta	
CASSLDDGYTF	Han; 2013; dietary	beta	
CASSLDRGDTQYF	Han; 2013; dietary	beta	
CASSLEQSAMNTEAFF	Han; 2013; dietary	beta	
CASSLGGELFF	Han; 2013; dietary	beta	
CASSLGGSGSYEQYF	Han; 2013; dietary	beta	
CASSLGNTEAFF	Han; 2013; dietary	beta	
CASSLGQGAQTQYF	Han; 2013; dietary	beta	
CASSLGRVEAFF	Han; 2013; dietary	beta	
CASSLLGGVQETQYF	Han; 2013; dietary	beta	
CASSLRGTSSYNSPLHF	Han; 2013; dietary	beta	
CASSLSGGSWTEAFF	Han; 2013; dietary	beta	
CASSLSGWTEAFF	Han; 2013; dietary	beta	

CASSLSQGGHNEQFF	Han; 2013; dietary	beta	
CASSLTASNQPQHF	Han; 2013; dietary	beta	
CASSPAGYNEQFF	Han; 2013; dietary	beta	
CASSPGLVVDEQYF	Han; 2013; dietary	beta	
CASSPPGQGITDTQYF	Han; 2013; dietary	beta	
CASSPRNKRKADQPQHF	Han; 2013; dietary	beta	
CASSPRVDRGLHEQYF	Han; 2013; dietary	beta	
CASSPTSGRTTSYEQYF	Han; 2013; dietary	beta	
CASSPWDGSSYEQYF	Han; 2013; dietary	beta	
CASSQDGGDYGYTF	Han; 2013; dietary	beta	
CASSQDGGTYNEQFF	Han; 2013; dietary	beta	
CASSQGRGKVYEQYF	Han; 2013; dietary	beta	
CASSQTGLTNYGYTF	Han; 2013; dietary	beta	
CASSRGVEQYF	Han; 2013; dietary	beta	
CASSRSGNTEAFF	Han; 2013; dietary	beta	
CASSSPLGGYGYTF	Han; 2013; dietary	beta	
CASSSPVRSGANVLTF	Han; 2013; dietary	beta	
CASSSTPGGLWYGYTF	Han; 2013; dietary	beta	
CASSWMTRIYNEQFF	Han; 2013; dietary	beta	
CASSYDSAYEQYF	Han; 2013; dietary	beta	
CASSYDVRSGNYEQYF	Han; 2013; dietary	beta	
CASSYSVAGAFF	Han; 2013; dietary	beta	
CASTAGFNQPQHF	Han; 2013; dietary	beta	
CASTFSYAGTDTQYF	Han; 2013; dietary	beta	
CATSTLQGGPRDEQFF	Han; 2013; dietary	beta	
CATSVTGSYGYTF	Han; 2013; dietary	beta	
CATTGSSYEQYF	Han; 2013; dietary	beta	
CAWNRGGSSPLHF	Han; 2013; dietary	beta	
CAWRRQGEEKLFF	Han; 2013; dietary	beta	
CAYSPGKNTEAFF	Han; 2013; dietary	beta	
CSAPGQRNTIYF	Han; 2013; dietary	beta	
CSARDPDSPPGGYTF	Han; 2013; dietary	beta	
CSVGGGEDYTF	Han; 2013; dietary	beta	
CASSQERGGKWAYEQYF	Han; 2013; dietary	beta	
CATSDLLTGNAFF	Han; 2013; dietary	beta	
CASSLNGGSYEQYF	Han; 2013; dietary	beta	
CASRPIPGQEKSSGANVLTF	Han; 2013; dietary	beta	
CASSFFPRTGSNEQFF	Han; 2013; dietary	beta	
CASSQEGVGGNYGYTF	Han; 2013; dietary	beta	
CASSQEQGTNYGYTF	Han; 2013; dietary	beta	
CASSQLTLGPAKNIQYF	Han; 2013; dietary	beta	

CASSQQLNYNSPLHF	Han; 2013; dietary	beta	
CASSVEGGGGPSTDTQYF	Han; 2013; dietary	beta	
CASSVPKGGFNEQFF	Han; 2013; dietary	beta	
CASGSGVTEAFF	Han; 2013; dietary	beta	
CASRPGATNYGYTF	Han; 2013; dietary	beta	
CASSLDGGVNGYTF	Han; 2013; dietary	beta	
CASSLDGVQIYGYTF	Han; 2013; dietary	beta	
CASSLGNTEAFF	Han; 2013; dietary	beta	
CASSLGQGVGTEAFF	Han; 2013; dietary	beta	
CASSLGYEQYF	Han; 2013; dietary	beta	
CASSLPVAGGQETQYF	Han; 2013; dietary	beta	
CASSLTGGSYGYTF	Han; 2013; dietary	beta	
CASSLVWNTDTQYF	Han; 2013; dietary	beta	
CASSPDGTGIEQFF	Han; 2013; dietary	beta	
CASSPGAFTNTEAFF	Han; 2013; dietary	beta	
CASSPWGSYEQYF	Han; 2013; dietary	beta	
CASSPYIGEVGNTEAFF	Han; 2013; dietary	beta	
CASSQEESADTQYF	Han; 2013; dietary	beta	
CASSQELGQSSYNSPLHF	Han; 2013; dietary	beta	
CASSQERGTAYGYTF	Han; 2013; dietary	beta	
CASSQERSADTQYF	Han; 2013; dietary	beta	
CASSQPQGWGNTEAFF	Han; 2013; dietary	beta	
CASSSYTGELFF	Han; 2013; dietary	beta	
CASSYIQGNQPQHF	Han; 2013; dietary	beta	
CASSYQGGGTDTQYF	Han; 2013; dietary	beta	
CASTRTGTGPATNEKLFF	Han; 2013; dietary	beta	
CSASLQENTEAFF	Han; 2013; dietary	beta	
CSVQERDSANYGYTF	Han; 2013; dietary	beta	
CASRTGNQPQHF	Han; 2013; dietary	beta	
CASRGGNTEAFF	Han; 2013; dietary	beta	
CASSFRVGYNEQFF	Han; 2013; dietary	beta	
CASIAGNTEAFF	Han; 2013; dietary	beta	
CASSSSYEQYF	Han; 2013; dietary	beta	
CASSLGNRPEAFF	Han; 2013; dietary	beta	
CASSFGSDTQYF	Han; 2013; dietary	beta	
CAWRSGGASPLHF	Han; 2013; dietary	beta	
CASKAGEFEFF	Han; 2013; dietary	beta	
CASSLEYEQYF	Han; 2013; dietary	beta	
CASSRLGGRAGETQYF	Han; 2013; dietary	beta	
CATTGGLGTEAFF	Han; 2013; dietary	beta	
CAGARGNQPQHF	Han; 2013; dietary	beta	

CAGTSGNQPQHF	Han; 2013; dietary	beta	
CASEPLAGTNEQFF	Han; 2013; dietary	beta	
CASGQDWSSYNEQFF	Han; 2013; dietary	beta	
CASMTGNSNQPQHF	Han; 2013; dietary	beta	
CASRPANQETQYF	Han; 2013; dietary	beta	
CASRSMDTYEQYF	Han; 2013; dietary	beta	
CASRTGTFFNQPQHF	Han; 2013; dietary	beta	
CASSAPEGGGFTDTQYF	Han; 2013; dietary	beta	
CASSATTGFSEKLFF	Han; 2013; dietary	beta	
CASSFGTAGTQYF	Han; 2013; dietary	beta	
CASSGGNQPQHF	Han; 2013; dietary	beta	
CASSGQATSYEQYF	Han; 2013; dietary	beta	
CASSHSRDRVGEKLFF	Han; 2013; dietary	beta	
CASSIAEGTIYNEQFF	Han; 2013; dietary	beta	
CASSIPGRRETQYF	Han; 2013; dietary	beta	
CASSISSDGYTF	Han; 2013; dietary	beta	
CASSLDEGYTGELFF	Han; 2013; dietary	beta	
CASSLDSSNQPQHF	Han; 2013; dietary	beta	
CASSLGAGANVLTF	Han; 2013; dietary	beta	
CASSLGGLAGEQYF	Han; 2013; dietary	beta	
CASSLGGQLFF	Han; 2013; dietary	beta	
CASSLGLAGEQYF	Han; 2013; dietary	beta	
CASSLGSKSTQYF	Han; 2013; dietary	beta	
CASSLGSNEQFF	Han; 2013; dietary	beta	
CASSLGSRETQYF	Han; 2013; dietary	beta	
CASSLGTSEQYF	Han; 2013; dietary	beta	
CASSLGTTSNEQYF	Han; 2013; dietary	beta	
CASSLLNTEAFF	Han; 2013; dietary	beta	
CASSLSRDNYNEQFF	Han; 2013; dietary	beta	
CASSLVGVADTQYF	Han; 2013; dietary	beta	
CASSPEPTGSNEQFF	Han; 2013; dietary	beta	
CASSPGQGVTEAFF	Han; 2013; dietary	beta	
CASSPGTVYGYTF	Han; 2013; dietary	beta	
CASSPQGVGADYGYTF	Han; 2013; dietary	beta	
CASSPRAEDWTYYGYTF	Han; 2013; dietary	beta	
CASSQDGISGSGEQYF	Han; 2013; dietary	beta	
CASSQGATSNQPQHF	Han; 2013; dietary	beta	
CASSRTSGGTGETQYF	Han; 2013; dietary	beta	
CASSSDSHYSNQPQHF	Han; 2013; dietary	beta	
CASSSLGGRAGETQYF	Han; 2013; dietary	beta	
CASSTERLGDGYTF	Han; 2013; dietary	beta	

CASSTGNQETQYF	Han; 2013; dietary	beta	
CASSTGNQPQHF	Han; 2013; dietary	beta	
CASSTRQGTTNTGELFF	Han; 2013; dietary	beta	
CASSTTGYGELFF	Han; 2013; dietary	beta	
CASSWGTDYEQYF	Han; 2013; dietary	beta	
CASSYGTLDPYGYTF	Han; 2013; dietary	beta	
CASSYSANNYGYTF	Han; 2013; dietary	beta	
CASSYTGLEQYF	Han; 2013; dietary	beta	
CASSYVGGAEAFF	Han; 2013; dietary	beta	
CASSYVREDYGYTF	Han; 2013; dietary	beta	
CASTGGYNSPLHF	Han; 2013; dietary	beta	
CASTLGGEQYF	Han; 2013; dietary	beta	
CASVQGNTEAFF	Han; 2013; dietary	beta	
CATSTYEGADQPQHF	Han; 2013; dietary	beta	
CAWSVGGIQPQHF	Han; 2013; dietary	beta	
CSAPGHLNYGYTF	Han; 2013; dietary	beta	
CSARDGGGDWEKLFF	Han; 2013; dietary	beta	
CSAREGQFSGNTIYF	Han; 2013; dietary	beta	
CASSYFGGPGNTIYF	Han; 2013; dietary	beta	
CASSQDRRSSYNSPLHF	Han; 2013; dietary	beta	
CASSQDLQTTFYEQYF	Han; 2013; dietary	beta	
CASSAVGGAYEQYF	Han; 2013; dietary	beta	
CSVTTGGQEAFF	Han; 2013; dietary	beta	
CSVALGAVRSTDTQYF	Han; 2013; dietary	beta	
CSARDIVNANTGELFF	Han; 2013; dietary	beta	
CSAPIQAGTEAFF	Han; 2013; dietary	beta	
CATSRDRSWDSPLHF	Han; 2013; dietary	beta	
CASTNGGMNTEAFF	Han; 2013; dietary	beta	
CASSYSVGNTGELFF	Han; 2013; dietary	beta	
CASSVGVVYEQYF	Han; 2013; dietary	beta	
CASSSSTVSGNTIYF	Han; 2013; dietary	beta	
CASSQQAPTSSYNSPLHF	Han; 2013; dietary	beta	
CASSQDLSWESPLHF	Han; 2013; dietary	beta	
CASSPVLGAFFGYGYTF	Han; 2013; dietary	beta	
CASSPTWTGGNEQFF	Han; 2013; dietary	beta	
CASSPDRPPIYNEQFF	Han; 2013; dietary	beta	
CASSPAVAGGRDTQYF	Han; 2013; dietary	beta	
CASSNPGLQETQYF	Han; 2013; dietary	beta	
CASSNDRARAKNIQYF	Han; 2013; dietary	beta	
CASSLVQDVGDEAFF	Han; 2013; dietary	beta	
CASSLTSGPLYEQFF	Han; 2013; dietary	beta	

CASSLRGGGETQYF	Han; 2013; dietary	beta	
CASSLNLGNTEAFF	Han; 2013; dietary	beta	
CASSLGETQYF	Han; 2013; dietary	beta	
CASSLFMGWEQYF	Han; 2013; dietary	beta	
CASSLEAGTLDYGYTF	Han; 2013; dietary	beta	
CASSIGTGGPYEQYF	Han; 2013; dietary	beta	
CASSHPSSPHEKLFF	Han; 2013; dietary	beta	
CASSFSGGAGEQFF	Han; 2013; dietary	beta	
CASSESGTGIGSQPQHF	Han; 2013; dietary	beta	
CASSELTRGTDTQYF	Han; 2013; dietary	beta	
CASSDGLAGFSTDTQYF	Han; 2013; dietary	beta	
CASRLTDTQYF	Han; 2013; dietary	beta	
CASQQTGGFNEQYF	Han; 2013; dietary	beta	
CASKTGVSYNEQFF	Han; 2013; dietary	beta	
CASGGGMGGQPQHF	Han; 2013; dietary	beta	
CAGTTGYEQYF	Han; 2013; dietary	beta	
CALGGLPTLGDTPTDKLIF	Han; 2013; dietary	delta	
CALCLLADWGYTDKLIF	Han; 2013; dietary	delta	
CALAPLPTLGDTGPDKLIF	Han; 2013; dietary	delta	
CALGDGGGFYTSRVLGGYAFVTTDKLIF	Han; 2013; dietary	delta	
CALGELPYWALRGADKLIF	Han; 2013; dietary	delta	
CALGGYADKLIF	Han; 2013; dietary	delta	
CASSPSYGGYAVDKLIF	Han; 2013; dietary	delta	
CACDTLLGDTLLTAQLFF	Han; 2013; dietary	delta	
CACDTLLGENKLIF	Han; 2013; dietary	delta	
CAFNRGLLYTDKLIF	Han; 2013; dietary	delta	
CALGAFLPRYWGPRHTDKLIF	Han; 2013; dietary	delta	
CALGDPSLPLNWGIRGHGIQLIF	Han; 2013; dietary	delta	
CALGEARPSYWGIRTTDKLIF	Han; 2013; dietary	delta	
CALGEFFPRYWGTTYTDKLIF	Han; 2013; dietary	delta	
CALGEKPPFLSKVLGDTHYTDKLIF	Han; 2013; dietary	delta	
CALGELIGWGPKDADKLIF	Han; 2013; dietary	delta	
CALGELQPRYWGRRFDKTKLFF	Han; 2013; dietary	delta	
CALGELRSLLHLHWGIRTDKLIF	Han; 2013; dietary	delta	
CALGERFRGYWGIQYTDKLIF	Han; 2013; dietary	delta	
CALGERHPSYWGNKGHTDKLIF	Han; 2013; dietary	delta	
CALGFPPVLGDPYTDKLIF	Han; 2013; dietary	delta	
CALGGSGISYVGILGKLIF	Han; 2013; dietary	delta	
CALGGTSYVPWGIVRRDKLIF	Han; 2013; dietary	delta	
CALGKGGNGVYWGSTRPLIF	Han; 2013; dietary	delta	
CALGNEAFRLVLGETDKLIF	Han; 2013; dietary	delta	

CALGPLSTPPYWGILGTDKLIF	Han; 2013; dietary	delta	
CALGPRFLRGVVGIRYTDKLIF	Han; 2013; dietary	delta	
CALGVLPTLGDQGPTDKLIF	Han; 2013; dietary	delta	
CALRGGRSPVLGDTLKRRTDKLIF	Han; 2013; dietary	delta	
CALGGLPTLGDTPTDKLIF	Han; 2013; dietary	delta	
CALGELRSLLHLHWGIRTDKLIF	Han; 2013; dietary	delta	
CALGERSPSYWGPHFTDKLIF	Han; 2013; dietary	delta	
CAASAGGPQTTDKLIF	Han; 2013; dietary	delta	
CALGDGGGFYTSRVLGGYAFVTTDKLIF	Han; 2013; dietary	delta	
CALGELVRSYFGIRGGKLIF	Han; 2013; dietary	delta	
CALGERLPNYWGTLYTDKLIF	Han; 2013; dietary	delta	
CALGERRPSYWGIRRGPLIF	Han; 2013; dietary	delta	
CALGGSGISYVGILGKLIF	Han; 2013; dietary	delta	
CAASPLVGNTDKLIF	Han; 2013; dietary	delta	
CACDTVGIQSDKLIF	Han; 2013; dietary	delta	
CALCLLADWGYTDKLIF	Han; 2013; dietary	delta	
CALGDPPSRGARPDKLIF	Han; 2013; dietary	delta	
CALGDPTGPYWGKYYLSYTDKLIF	Han; 2013; dietary	delta	
CALGECHPSYWGRPINTDKLIF	Han; 2013; dietary	delta	
CALGECPTRHPTGGYIPYTDKLIF	Han; 2013; dietary	delta	
CALGEFFPRYWGTTYTDKLIF	Han; 2013; dietary	delta	
CALGELPYWALRGADKLIF	Han; 2013; dietary	delta	
CALGELQPRYWGRRFDKTKLFF	Han; 2013; dietary	delta	
CALGELRPSYVFGGYAYKLIF	Han; 2013; dietary	delta	
CALGELSPRYWGIGYTDKLIF	Han; 2013; dietary	delta	
CALGELSRPADWGILIYTDKLIF	Han; 2013; dietary	delta	
CALGEPTGAFLLTGGFTDKLIF	Han; 2013; dietary	delta	
CALGEQNPRYWGASYTDKLIF	Han; 2013; dietary	delta	
CALGERLPSYWGISYTDKLIF	Han; 2013; dietary	delta	
CALGESYVSYWGGYLYTDKLIF	Han; 2013; dietary	delta	
CALGEYLPRYWGIHGTDKLIF	Han; 2013; dietary	delta	
CALGFPPVLGDPYTDKLIF	Han; 2013; dietary	delta	
CALGELPLLGDTLRSYTDKLIF	Han; 2013; dietary	delta	
CALGVPLLQVKLGDTKGLLGDTDKLIF	Han; 2013; dietary	delta	
CALGNIWGVTDKLIF	Han; 2013; dietary	delta	
CALGELVLRYWGGRMDKLIF	Han; 2013; dietary	delta	
CALGEGAGILTGDKLIF	Han; 2013; dietary	delta	
CAFLRIRPDKLIF	Han; 2013; dietary	delta	
CALVSNPPPRYPGVRDTDKLIF	Han; 2013; dietary	delta	
CALVADYWGIGTDKLIF	Han; 2013; dietary	delta	
CALGPRFLRRGIRADKLIF	Han; 2013; dietary	delta	

CALGPRAHQRTGDRVTAQLFF	Han; 2013; dietary	delta	
CALGNPKGTSYGLRGIPPYTDKLIF	Han; 2013; dietary	delta	
CALGKGGSYVVHFYWGIESTDKLIF	Han; 2013; dietary	delta	
CALGISLLRLGDMISDKLIF	Han; 2013; dietary	delta	
CALGEPPFLRRYRYTDKLIF	Han; 2013; dietary	delta	
CALGELPTWTYWGIDKLIF	Han; 2013; dietary	delta	
CALGELIRGYWGIRYTDKLIF	Han; 2013; dietary	delta	
CALGDPPPHRSLLYRYKLIF	Han; 2013; dietary	delta	
CALGALPTLGDRGVDKLIF	Han; 2013; dietary	delta	
CALDTPKHSSGGYFKRTDKLIF	Han; 2013; dietary	delta	
CASHFLRAGYAKLIF	Han; 2013; dietary	delta	
CASCPGVGDNDKLIF	Han; 2013; dietary	delta	
CAQTTYWGMGGQYTDKLIF	Han; 2013; dietary	delta	
CAPFSWPDKLIF	Han; 2013; dietary	delta	
CALVSGGFPSYADKLIF	Han; 2013; dietary	delta	
CALRWGIRGADKLIF	Han; 2013; dietary	delta	
CALRVFTYWGDTDKLIF	Han; 2013; dietary	delta	
CALGVRIFPPSLLGDTGYGGVLIF	Han; 2013; dietary	delta	
CALGSLLINWGIVTDKLIF	Han; 2013; dietary	delta	
CALGSGAYPYRTGGRELIF	Han; 2013; dietary	delta	
CALGPPPFLIGSWDTRQMFF	Han; 2013; dietary	delta	
CALGPGAFLRSWGQKLIF	Han; 2013; dietary	delta	
CALGNSYWGIPYTDKLIF	Han; 2013; dietary	delta	
CALGNHWADKLIF	Han; 2013; dietary	delta	
CALGLPIGLGDSYLYKLIF	Han; 2013; dietary	delta	
CALGKRPYPLYWGIRGYTDKLIF	Han; 2013; dietary	delta	
CALGFYWGEYTDKLIF	Han; 2013; dietary	delta	
CALGEYSRLTGVYTDKLIF	Han; 2013; dietary	delta	
CALGEWFPGYFLTKFRNTDKLIF	Han; 2013; dietary	delta	
CALGESVRWVFGEYTDKLIF	Han; 2013; dietary	delta	
CALGERYPKYWGAPGTDKLIF	Han; 2013; dietary	delta	
CALGERSYVPYWGTGRGTDKLIF	Han; 2013; dietary	delta	
CALGERPRPRYWGNALTAQLFF	Han; 2013; dietary	delta	
CALGERGPRYWGIAYTDKLIF	Han; 2013; dietary	delta	
CALGERFPWPHTDKLIF	Han; 2013; dietary	delta	
CALGEPSDSAYWGIRGNTDKLIF	Han; 2013; dietary	delta	
CALGEPRAVLGDTLGDKLIF	Han; 2013; dietary	delta	
CALGEPLPSYWGPRGSDKLIF	Han; 2013; dietary	delta	
CALGEPDSTFVRGGYAGNTDKLIF	Han; 2013; dietary	delta	
CALGEPDLPTTWYTDKLIF	Han; 2013; dietary	delta	
CALGEPALQLGVNKLIF	Han; 2013; dietary	delta	

CALGENPPPYLGGYPYTDKLIF	Han; 2013; dietary	delta	
CALGENFPSSWGIHRYTDKLIF	Han; 2013; dietary	delta	
CALGELVYPGGYYGRETAQLFF	Han; 2013; dietary	delta	
CALGELVFLLRAGLIF	Han; 2013; dietary	delta	
CALGELSDLQCVLGDRPTRPLIF	Han; 2013; dietary	delta	
CALGELRRIYWGIRIDKLIF	Han; 2013; dietary	delta	
CALGELLPRYWGIGGTDKLIF	Han; 2013; dietary	delta	
CALGELLASETYTGGSVVLYRARKTDKLIF	Han; 2013; dietary	delta	
CALGEKGNMPLGDIIDKLIF	Han; 2013; dietary	delta	
CALGEHEVHPGGYWYTDKLIF	Han; 2013; dietary	delta	
CALGEGTGDFGRWGILVYTDKLIF	Han; 2013; dietary	delta	
CALGEGPFLRTGGLYTDKLIF	Han; 2013; dietary	delta	
CALGEGPAPIWGIRRRSYTDKLIF	Han; 2013; dietary	delta	
CALGEFYWGILSDKLIF	Han; 2013; dietary	delta	
CALGEEIPTGGYPDKLIF	Han; 2013; dietary	delta	
CALGEDPSFLRLGIRYTDKLIF	Han; 2013; dietary	delta	
CALGDWRSSYFNWGISSPDKLIF	Han; 2013; dietary	delta	
CALGDPSEEAHTGGYNTDKLIF	Han; 2013; dietary	delta	
CALGDLLGLPRGPTDKLIF	Han; 2013; dietary	delta	
CALGDFPTWGGVPDKLIF	Han; 2013; dietary	delta	
CALGASLGDNSPDKLIF	Han; 2013; dietary	delta	
CALGALGSLPTHWGIRATDKLIF	Han; 2013; dietary	delta	
CALGALGLRGSLGVYRKLIF	Han; 2013; dietary	delta	
CALEAPYTDKLIF	Han; 2013; dietary	delta	
CALAQPSSNLLIHWGILDKLIF	Han; 2013; dietary	delta	
CAITGSKGTDKLIF	Han; 2013; dietary	delta	
CAFRHGPNYPLIYWGISKLIF	Han; 2013; dietary	delta	
CAFRGLWGYTDKLIF	Han; 2013; dietary	delta	
CAFLALPMYTDKLIF	Han; 2013; dietary	delta	
CACVKAFLKRGDTPYTDKLIF	Han; 2013; dietary	delta	
CACTFLGLGGSNTDKLIF	Han; 2013; dietary	delta	
CACETWGIKGTDKLIF	Han; 2013; dietary	delta	
CACERGGYAFTDKLIF	Han; 2013; dietary	delta	
CACDTVYGGYWATDKLIF	Han; 2013; dietary	delta	
CACDSRTSTWGIRMADKLIF	Han; 2013; dietary	delta	
CALGELPPPGGYFDKLIF	Han; 2013; dietary	delta	
CAFKGLLGGSVGLIF	Han; 2013; dietary	delta	
CALGDSSLGGWGILSSTDKLIF	Han; 2013; dietary	delta	
CALGVLHWGNSLTAQLFF	Han; 2013; dietary	delta	
CALPFSYWGIRLVGTDKLIF	Han; 2013; dietary	delta	
CASTGAVGKSPKLIF	Han; 2013; dietary	delta	

CAASAGLPGGLGYTDKLIF	Han; 2013; dietary	delta	
CAASALRGSFDKLIF	Han; 2013; dietary	delta	
CACDHDYGTGGVRKLIF	Han; 2013; dietary	delta	
CACRLPTRWGIGYTDKLIF	Han; 2013; dietary	delta	
CACRPSYGGIVKLIF	Han; 2013; dietary	delta	
CAFILTIYGPGGITDKLIF	Han; 2013; dietary	delta	
CAFPTGGLLGDTDKLIF	Han; 2013; dietary	delta	
CAFVGGPYTDKLIF	Han; 2013; dietary	delta	
CALADLRPGGYSAQLFF	Han; 2013; dietary	delta	
CALEVVHHPIRYTDKLIF	Han; 2013; dietary	delta	
CALGAHLRNYWGPLYTDKLIF	Han; 2013; dietary	delta	
CALGAYPPGGTGRYTDKLIF	Han; 2013; dietary	delta	
CALGDFLPSYWGIRGTDKLIF	Han; 2013; dietary	delta	
CALGDPFQNYQGPYTDKLIF	Han; 2013; dietary	delta	
CALGEAFLSYWGTNHDKLIF	Han; 2013; dietary	delta	
CALGEGGGVLRNPYTDKLIF	Han; 2013; dietary	delta	
CALGEHGAAFLPYWGIRRGKLIF	Han; 2013; dietary	delta	
CALGEIYRGYWGIRAGDKLIF	Han; 2013; dietary	delta	
CALGELHWGTRYTDKLIF	Han; 2013; dietary	delta	
CALGELLRTGGLAQLFF	Han; 2013; dietary	delta	
CALGELMLGRWGEYTDKLIF	Han; 2013; dietary	delta	
CALGELNLPQYWGPLVGTDKLIF	Han; 2013; dietary	delta	
CALGELPPWGIPYTDKLIF	Han; 2013; dietary	delta	
CALGELRLRWMGDTLFLQLTDKLIF	Han; 2013; dietary	delta	
CALGELRRGIRGQRIGTDKLIF	Han; 2013; dietary	delta	
CALGELSRPSYYYDPSYTDKLIF	Han; 2013; dietary	delta	
CALGELSSPHTGGYYTDKLIF	Han; 2013; dietary	delta	
CALGELSYRGGWGIRADKLIF	Han; 2013; dietary	delta	
CALGENKFVFGGLIVLTAQLFF	Han; 2013; dietary	delta	
CALGEPIGPPLLGVYTDKLIF	Han; 2013; dietary	delta	
CALGEPQTFLPRYWGGTYTDKLIF	Han; 2013; dietary	delta	
CALGEPSTGGSDKLIF	Han; 2013; dietary	delta	
CALGEQWILRGDTDKLIF	Han; 2013; dietary	delta	
CALGERLRGYALKTDKLIF	Han; 2013; dietary	delta	
CALGERLSPYYTDKLIF	Han; 2013; dietary	delta	
CALGERPSYGWGFGWTDKLIF	Han; 2013; dietary	delta	
CALGETTLSYWGIRYTDKLIF	Han; 2013; dietary	delta	
CALGGGLPTSGGYRSYTDKLIF	Han; 2013; dietary	delta	
CALGHRAPSRAQPYWGILAYTDKLIF	Han; 2013; dietary	delta	
CALGKPAKSYWGMRYTDKLIF	Han; 2013; dietary	delta	
CALGPLPGGYSSWDTRQMFF	Han; 2013; dietary	delta	

CALGQRIPSYWGIAGSTDKLIF	Han; 2013; dietary	delta	
CALGVISPSYWGPQYTDKLIF	Han; 2013; dietary	delta	
CALGVSSSAGDLLTDKLIF	Han; 2013; dietary	delta	
CALKPGGYSLTDKLIF	Han; 2013; dietary	delta	
CALMAGPYTDKLIF	Han; 2013; dietary	delta	
CASVCYGNGHISRLDKLIF	Han; 2013; dietary	delta	
LVGGSGGYNKLI	Christophersen; 2022; Pheno	alpha	
AVEGGSNYKLT	Christophersen; 2022; Pheno	alpha	
ASSIRATDTQY	Christophersen; 2022; Pheno	beta	
ASSIRSTDTQY	Christophersen; 2022; Pheno	beta	
SASRTSGRAGDEQF	Christophersen; 2022; Pheno	beta	
ALSDQGSSASKII	Christophersen; 2022; Pheno	alpha	
ALSDPTGTASKLT	Christophersen; 2022; Pheno	alpha	
VVSGGYYGGSQGNLI	Christophersen; 2022; Pheno	alpha	
VVNPGGGNKLT	Christophersen; 2022; Pheno	alpha	
AVPNAGGTSYGKLT	Christophersen; 2022; Pheno	alpha	
AVEREGAQKLV	Christophersen; 2022; Pheno	alpha	
AVGQGAQKLV	Christophersen; 2022; Pheno	alpha	
AYRSEQGAQKLV	Christophersen; 2022; Pheno	alpha	
ASSQGQDTEAF	Christophersen; 2022; Pheno	beta	
ASSLTVTDTQY	Christophersen; 2022; Pheno	beta	
SASDSLNTEAF	Christophersen; 2022; Pheno	beta	
SASRQVADTQY	Christophersen; 2022; Pheno	beta	

Supplementary Table S2: All published gluten specific TCR sequences.
